# Graph-theoretic active learning for the closed-loop discovery of stochastic heterogeneous composites

**DOI:** 10.1371/journal.pone.0353692

**Published:** 2026-08-18

**Authors:** Lei Qiu, Shuxin Zhang, Yongbin Yang, Mengdie Wang

**Affiliations:** 1 Ningbo University of Technology, Ningbo, China; 2 UC Berkeley, Berkeley, California, United States of America; 3 University of Southern California, Los Angeles, United States of America; 4 Shanghai Lixin University of Accounting and Finance, Shanghai, China; Asia Pacific University of Technology & Innovation, MALAYSIA

## Abstract

The inverse design of resilient infrastructure materials is hindered by the combinatorial complexity inherent in optimizing stochastic, heterogeneous microstructures. Traditional Euclidean deep learning architectures, such as Convolutional Neural Networks (CNNs), fail to efficiently encode the sparse, non-local topology of disordered porous media, resulting in prohibitive computational redundancy. This work articulates a comprehensive graph-theoretic framework for the predictive modeling and optimization of self-healing cementitious composites. We propose a robust methodology for discretizing volumetric tomography into permutation-invariant heterogeneous graphs, where nodes encode discrete physical entities (aggregates, pores, and microcapsules) and edges represent mechanical and hydraulic connectivity. To capture global fracture dynamics and local transport phenomena, we employ specialized architectures, specifically Pore-GNNs and Long-Short-Edge MeshGraphNets, which function as high-speed surrogate models for multiphysics simulations. Computational validation against high-fidelity numerical solvers confirms the system’s efficacy: the proposed architecture achieves approximately $1.1\times 10^{5}$-fold acceleration (inference plus preprocessing) in permeability prediction relative to Lattice Boltzmann Method simulations, while maintaining a prediction accuracy of *R*^2^ > 0.94 (mean±std over five random splits); the inference-only speedup, which excludes one-time graph-construction preprocessing, is approximately $1.8\times 10^{6}$-fold. We note that the end-to-end figure ($1.1\times 10^{5}$) supersedes the inference-only figure quoted in the original submission metadata. Furthermore, the framework autonomously navigated the high-dimensional design space to isolate a non-intuitive geometric optimum within just 30 high-fidelity oracle evaluations: anisotropic microcapsules ($\rho_{c}\approx 2.5$) oriented at $45^{\circ }$ relative to the shear plane. This configuration is predicted to resolve the conflicting physical constraints of the system, maximizing the probability of crack interception for effective healing. These results demonstrate that physics-informed graph learning can effectively decouple prediction accuracy from computational intensity, offering a scalable paradigm for the discovery of advanced functional materials.

## Introduction

The paradigm of materials discovery is undergoing a fundamental inversion. Traditional engineering relied on the forward characterization of existing substances where measurements followed synthesis. In contrast, modern computational approaches seek to navigate the inverse path by defining desired functional properties and algorithmically identifying the microstructural configurations that yield them [1]. This inverse design challenge is particularly acute in the domain of civil infrastructure and specifically regarding self-healing cementitious composites. These materials are not ordered crystal lattices but rather stochastic heterogeneous systems defined by extreme disorder. The macroscopic resilience of such composites is governed by complex path-dependent interactions between diverse phases. These phases include aggregates, cement paste, voids, and functional inclusions like microcapsules [2]. Consequently, the design space is combinatorially explosive, which renders exhaustive Edisonian trial-and-error approaches intractable. Recently, large-scale deep learning initiatives have demonstrated the potential to stabilize new inorganic crystals [3], yet applying these insights to the mesoscale disorder of concrete remains an open challenge.

To navigate this high-dimensional manifold, researchers have increasingly turned to data-driven surrogate models. However, a significant geometric mismatch persists in the current literature. The prevailing deep learning architectures are primarily Convolutional Neural Networks (CNNs), which are predicated on Euclidean grid structures [4]. While effective for image data, pixel-based or voxel-based representations are fundamentally ill-suited for porous media. Representing a sparse fracture network within a dense voxel grid incurs massive computational redundancy because the vast majority of computational resources are spent processing empty space rather than the topological features of interest. Furthermore, CNNs lack rotational invariance without extensive data augmentation. This limitation is a critical flaw when modeling isotropic or anisotropic stress fields in disordered media [5]. Recent advances in equivariant neural networks suggest that enforcing symmetry constraints directly within the architecture can significantly improve data efficiency for physical systems [6].

Beyond the geometric limitations of current machine learning models, the physical design of self-healing composites presents a multi-objective optimization problem characterized by conflicting constraints. The functional inclusions, such as healing agent microcapsules, must possess sufficient structural robustness to survive the high-shear environment of industrial mixing and casting processes. Conversely, these same capsules must remain brittle enough to rupture immediately upon the propagation of a microcrack to release the healing agent effectively. Navigating this trade-off requires a precise understanding of how local microstructural topology influences stress concentrations around the capsules. Conventional homogenization techniques often fail to capture these localized, topology-dependent fracture dynamics, which necessitates high-fidelity modeling at the mesoscale level.

Addressing these multiphysics challenges typically requires numerical simulations such as the Lattice Boltzmann Method (LBM) for fluid transport or the Discrete Element Method (DEM) for fracture mechanics. While these solvers offer high accuracy, they are computationally prohibitive for iterative optimization. A single high-resolution LBM simulation of permeability in a complex porous network can require hours of computation time [7]. When optimization algorithms require evaluating thousands of potential microstructural candidates, the computational cost becomes an insurmountable bottleneck. Therefore, the acceleration of materials discovery relies on the development of surrogate models that can approximate the physics of these solvers with orders of magnitude greater speed while retaining high fidelity.

This work posits that stochastic heterogeneous composites are more naturally represented as graphs rather than images. By discretizing material volumes into permutation-invariant heterogeneous graphs, where nodes represent physical entities and edges encode mechanical or hydraulic adjacency, the inductive biases of Geometric Deep Learning can be leveraged [8]. Graph Neural Networks (GNNs) offer a resolution to the sparsity problem by operating strictly on the connected topology of the material, which effectively decouples computational cost from the volumetric resolution. This topological approach allows for the efficient encoding of non-local interactions that are critical for predicting fracture propagation and fluid transport in disordered media.

To operationalize this hypothesis, a Graph-Theoretic Active Learning (GTAL) framework is introduced. The GTAL framework functions as a closed-loop system that integrates three distinct modules: a topological discretization pipeline, a graph-based physics surrogate, and a Bayesian optimizer. First, raw tomographic data is converted into a heterogeneous graph structure where nodes encode the physical properties of aggregates, pores, and microcapsules. Second, these graphs form the input for a specialized GNN architecture that predicts physical properties, such as permeability and survival rate, by learning the message-passing dynamics between connected nodes. Finally, an acquisition function guides the exploration of the design space by selecting candidate microstructures that maximize the expected improvement in material performance. This loop allows the system to autonomously learn the complex mapping between microstructural topology and macroscopic functionality.

The contributions of this work are summarized as follows:

- Unlike prior graph-based pore surrogates such as Pore-GNN [9], which use homogeneous graphs over a single pore network, we encode three distinct node types and two physically distinct edge types (hydraulic and mechanical) in a single heterogeneous multigraph, enabling simultaneous multiphysics inference from a unified topology.
- Standard MeshGraphNets [10] use exclusively local physical contact edges. Our spectral augmentation addresses the over-smoothing pathology that prevents deep GNNs from propagating fracture crack-tip stress fields across the simulation domain, achieving *R*^2^ > 0.94 (mean±std over five random splits) with approximately $1.1\times 10^{5}$-fold acceleration over the coupled LBM and DEM solvers (including preprocessing).
- Prior works either build surrogates without optimization or use BO with simple ML models. GTAL tightly integrates the GNN physics surrogate into an uncertainty-aware acquisition loop, autonomously discovering non-intuitive anisotropic microcapsule configurations ($\rho_{c}\approx 2.5$, $45^{\circ }$ orientation) within 30 high-fidelity oracle evaluations.

The remainder of this paper is organized as follows. Section 2 reviews the related work concerning Euclidean deep learning limitations and the emergence of graph-based physics solvers. Section 3 details the methodology behind the GTAL framework, including the graph discretization process and the specific neural architectures employed. Section 4 presents the computational validation, comparing the surrogate model’s performance against ground-truth physics simulations and analyzing the optimal microstructures discovered. Finally, Section 5 concludes the study and outlines future directions for graph-based materials discovery.

## Related work

The computational discovery of heterogeneous composites sits at the intersection of geometric deep learning, computational mechanics, and autonomous design. This chapter reviews the state-of-the-art across five distinct pillars: microstructural characterization, surrogate modeling of transport phenomena, graph-based fracture mechanics, generative material synthesis, and active learning for inverse design. We identify specific technological gaps in these areas that the proposed GTAL framework aims to bridge.

### Deep learning for microstructural characterization

The digitization of material microstructure via X-ray CT has necessitated robust algorithms for quantifying topology. Early data-driven approaches relied heavily on CNNs to extract features from voxelized volumes. For instance, Elmorsy et al. [11] employed multi-scale convolutional architectures to predict effective properties directly from raw tomograms, while Alqahtani et al. [12] provided a comprehensive review of 2D/3D CNN applications in porous media. However, these Euclidean architectures struggle with the curse of dimensionality. As noted by Li et al. [13], increasing the resolution of 3D-CNNs to capture critical interfacial features like the ITZ results in cubic growth in memory requirements. This often forces researchers to employ aggressive downsampling or sliding-window techniques, which obscure the global connectivity governing transport phenomena.

Moreover, Euclidean architectures inherently lack rotational invariance. To predict the behavior of an isotropic porous medium, a standard CNN must be trained on all possible rotations of the input, necessitating massive data augmentation [5]. To mitigate this, recent work has explored spectral analysis and topological data analysis (TDA) to extract invariant descriptors. Salehi et al. [14] demonstrated that partitioning-based schemes could improve efficiency, but such methods typically break the continuity of the fracture network. Consequently, the field is shifting toward discrete topological models, specifically graphs and unstructured meshes, which offer a sparse, rotation-invariant description of material geometry that aligns more closely with physical intuition.

### Surrogate modeling of transport phenomena

The simulation of fluid transport in porous media is a primary bottleneck in materials design. While LBM provides ground-truth accuracy, its computational cost is prohibitive for iterative optimization. This has driven the development of GNN-based surrogates. Alzahrani [9] introduced “Pore-GNN,” a framework where message-passing operations along pore throats approximate Navier-Stokes solutions. By representing the pore space as a network of nodes, they achieved high-fidelity permeability predictions using a fraction of the parameters required by voxel-based solvers. Similarly, I’Anson et al. [15] recently validated that GNNs trained on LBM data could generalize across varying porosity regimes, effectively acting as high-speed inference engines for flow properties.

Despite these successes, most graph-based flow solvers rely on simplified skeletonization techniques that may discard geometric nuances critical to capillary dynamics. Zhao et al. [16] addressed this by integrating GNNs with traditional pore network modeling (PNM) to enhance the prediction of multiphase flow. Their results highlight that while pure graph learning captures topology, hybrid approaches that encode geometric attributes (such as throat radii and local roughness) into the node feature vectors yield superior convergence. This insight is central to our method, which enriches the graph representation with physical metadata derived from the raw X-ray CT scans.

### Graph-based fracture mechanics

Parallel to fluid dynamics, advancements in computational solid mechanics have leveraged mesh-based learning to model deformation and failure [17]. The MeshGraphNets architecture proposed by Pfaff et al. [10] established a paradigm for learning Lagrangian dynamics by encoding the simulation state into graph edges. This approach has been successfully extended to complex contact dynamics and plasticity. Maurizi et al. [18] demonstrated that GNNs could model elastic-plastic deformation in heterogeneous materials by learning the constitutive laws directly from displacement data, bypassing the need for explicit formulation of the yield surface.

However, modeling fracture propagation presents a distinct challenge due to the dynamic change in topology. Zheng et al. [19] and Liu et al. [20] employed physics-informed deep learning to predict crack paths in brittle materials, utilizing energy minimization principles within the loss function. While these models excel at single-physics fracture, the interaction between mechanical damage and hydraulic transport, which is the defining characteristic of self-healing systems, remains largely unexplored. Existing architectures typically treat solid mechanics and fluid flow as separate domains, whereas the effective design of resilient composites requires a coupled multiphysics solver that accounts for how stress concentrations (solid) drive crack opening (geometry) and subsequent healing agent flow (fluid).

### Generative models for material synthesis

The inverse aspect of materials design requires not just analyzing existing structures, but generating new ones. Generative models, such as Variational Autoencoders (VAEs) and Denoising Diffusion Probabilistic Models (DDPMs), have revolutionized this space. Yang et al. [21] applied diffusion models to the inverse design of mechanical metamaterials, generating complex lattice structures that match target stress-strain curves. Merchant et al. [3] further demonstrated the scalability of deep learning for discovering stable inorganic crystals, suggesting that generative AI can navigate the vast chemical space of materials.

Nevertheless, a critical limitation of image-based generative models in this context is their tendency to produce visually realistic but functionally suboptimal structures. Yazdani et al. [22] noted that while GANs can reproduce the texture of concrete, they often fail to enforce long-range connectivity constraints required for percolation. Furthermore, standard generative models operate in Euclidean space. Generating valid graphs that satisfy physical constraints (e.g., non-overlapping aggregates, connected pore networks) is significantly harder than generating pixel grids. This necessitates the use of graph-generative approaches or constructive algorithms that can build valid topologies from the ground up, ensuring that every generated candidate is physically realizable.

### Active learning and bayesian optimization

To close the loop between generation and simulation, Bayesian Optimization (BO) has become the standard for navigating high-dimensional design spaces. By utilizing Gaussian Processes (GPs) to model uncertainty, BO sequentially selects candidates that balance exploration and exploitation. Lookman et al. [23] provided the foundational information-theoretic perspective for this approach in materials science. Recent applications have been highly effective: Thomas et al. [24] utilized BO to optimize the fiber orientation in reinforced composites, significantly outperforming genetic algorithms in convergence speed. Similarly, Kim et al. [25] employed an active learning loop to accelerate the discovery of stable perovskites, reducing the number of required experiments by an order of magnitude.

In the specific domain of cementitious materials, Pessoa et al. [26] and Huang et al. [27] applied machine learning to predict healing efficiency. However, these studies largely operated in a forward prediction mode or utilized pre-defined datasets. They lacked a truly autonomous cycle where the system creates novel microstructural designs based on feedback from a surrogate physics model. The GTAL framework differentiates itself by integrating the GNN surrogate directly into the acquisition function, allowing the optimizer to query the physics of the material at minimal computational cost and thereby enabling the exploration of complex, non-intuitive design strategies that maximize microcapsule survival and healing efficacy.

### Positioning relative to graph-based pore surrogates

To clarify the incremental contribution of the present work, Table S1 in S1 Appendix provides a structured comparison of LSE-MGN against three representative graph-based approaches along five axes: graph type, edge physics, long-range propagation, multiphysics coupling, and closed-loop optimization. In brief, Pore-GNN [9] operates on a single-type pore-to-pore graph for permeability prediction only; MeshGraphNets [10] use local physical contact edges on simulation meshes with uniform edge types and no spectral augmentation; Reiser et al.’s graph-based fluid surrogates [7] predict permeability from homogeneous pore graphs. None of these methods handles coupled multiphysics (simultaneous transport and fracture) on a heterogeneous substrate, nor integrates spectral shortcuts for long-range fracture dynamics, nor embeds the surrogate within a closed-loop Bayesian optimization system.

Synthesizing these developments reveals a critical methodological gap. While GNNs have proven effective for isolated physical domains and BO has accelerated parameter tuning, no existing framework successfully couples topological representation with multiphysics surrogate modeling to navigate the survival-versus-healing conflict in composite design. Current approaches either simplify the geometry to the point of losing critical ITZ dynamics or rely on computationally expensive LBM/DEM solvers that preclude extensive exploration. Consequently, there is an urgent need for a closed-loop system capable of learning the complex mapping between stochastic graph topologies and competing macroscopic properties. This research addresses this necessity by proposing GTAL, a unified paradigm that bridges the divide between geometric deep learning and autonomous materials discovery.

## Methods

The GTAL framework is formulated to address the high-dimensional inverse problem of designing stochastic heterogeneous composites. Let the design space be denoted by a manifold $𝒳\subset {\mathbb{R}}^{d}$, parameterized by synthesis variables θ∈𝒳 (e.g., capsule volume fraction, aspect ratio, shell thickness). We seek to approximate the unknown mapping f:𝒳→𝒴, where $𝒴\in {\mathbb{R}}^{2}$ represents the objective space comprising permeability *y*_perm_ and microcapsule survival rate *y*_surv_.

**Fig 1:**
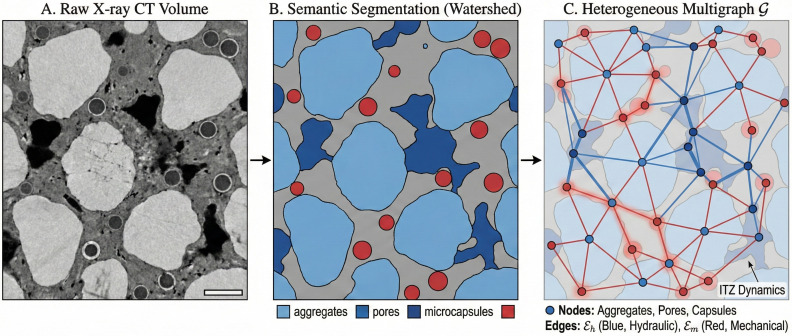
The GTAL Topological Discretization Pipeline. (A) Raw X-ray CT volume containing aggregates, pores, and microcapsules. (B) Semantic segmentation via marker-controlled watershed transform. (C) Conversion to a heterogeneous multigraph 𝒢. Blue edges (${ℰ}_{h}$) encode hydraulic conductance via the Maximal Ball method; Red edges (${ℰ}_{m}$) encode mechanical force chains via weighted Delaunay triangulation, capturing the Interfacial Transition Zone (ITZ) dynamics.

Evaluating f(θ) involves solving coupled partial differential equations (PDEs) for fluid dynamics and fracture mechanics, a process that is computationally intractable for exhaustive search. We therefore construct a surrogate model ${\hat{f}}_{\Phi }$, parameterized by a graph neural network with weights Φ, to approximate the physics manifold. The framework, depicted in Fig 1, comprises three sequentially coupled modules: (1) a topology-preserving discretization pipeline 𝒯; (2) a Long-Short-Edge MeshGraphNet (LSE-MGN) for multiphysics inference; and (3) a Bayesian optimization loop driven by an uncertainty-aware acquisition function.

### Topological discretization via heterogeneous multigraphs

Euclidean representations (voxels) introduce prohibitive sparsity and lack rotation invariance. We fundamentally recast the material volume Ω as a permutation-invariant heterogeneous graph 𝒢=(𝒱,ℰ). This mapping 𝒯:Ω→𝒢 preserves the topological genus of the pore network and the contact topology of the solid phase.

### Manifold-aware node feature engineering

The node set 𝒱 is semantically partitioned into $𝒱={𝒱}_{p}\cup {𝒱}_{a}\cup {𝒱}_{c}$ (pores, aggregates, capsules). The segmentation employs a marker-controlled watershed transform [28] on the binarized solid phase (Otsu threshold). Markers are seeded at local maxima of the distance transform with a minimum spacing of $d_{m}=5$ voxels. Over-segmentation is suppressed by merging regions whose shared boundary gradient is below a threshold $\tau_{\text{merge}}=0.15$ (normalized units). Isolated components with volume $V_{i}<8$ voxels^3^ are pruned as segmentation noise, and edges exceeding $3\bar{d}$ (three standard deviations above the mean edge length) are removed as physically implausible. Full pipeline pseudocode is provided in Algorithm S1 (S1 Appendix). A systematic sensitivity analysis over watershed marker spacing ($d_{m}$), pore-throat detection threshold (α), and synthetic segmentation noise (SNR) is reported in S3 Appendix; performance varies by ≤0.03 in *R*^2^ across these perturbations, confirming robustness. We recommend $d_{m}=5$ voxels and α=0.75 as defaults.

Following semantic segmentation via the marker-controlled watershed transform, we extract a feature vector ${𝐡}_{i}\in {\mathbb{R}}^{d_{f}}$ for each node $v_{i}$:


$$\begin{array}{c} {𝐡}_{i}={\left[{𝐱}_{i},V_{i},{𝒮}_{i},\text{vec}({𝐐}_{i}),E_{i},\nu_{i}\right]}^{T} \end{array}$$ (1)

Here, ${𝐱}_{i}$ is the centroid, $V_{i}$ is the volume, and ${𝒮}_{i}$ is the sphericity. Crucially, to encode the rotational anisotropy of microcapsules, we compute the inertia tensor ${𝐈}_{i}$ and perform an eigendecomposition ${𝐈}_{i}={𝐐}_{i}\Lambda_{i}{𝐐}_{i}^{T}$. The flattened eigenvector matrix $\text{vec}({𝐐}_{i})$ serves as a rotation-equivariant descriptor of orientation. $E_{i}$ and $\nu_{i}$ represent the phase-specific Young’s modulus and Poisson’s ratio, respectively.

### Physics-based edge construction

The edge set ℰ is a union of two disjoint sets, $ℰ={ℰ}_{h}\cup {ℰ}_{m}$, enabling the graph to switch dynamic modes between fluid and solid simulations.

#### Hydraulic Connectivity (${ℰ}_{h}$).

We define edges between pore nodes $v_{i},v_{j}\in {𝒱}_{p}$ based on the Maximal Ball Algorithm. An edge exists if the two pores share a constriction (throat) that limits non-wetting phase entry. The edge attribute ${𝐞}_{ij}^{h}$ explicitly encodes the Hagen-Poiseuille conductance:


$$\begin{array}{c} {𝐞}_{ij}^{h}={\left[\frac{r_{ij}^{4}}{\mu d_{ij}},\kappa_{ij}\right]}^{T} \end{array}$$ (2)

where $r_{ij}$ is the throat radius, $d_{ij}$ is the Euclidean distance, μ is fluid viscosity, and $\kappa_{ij}$ is a shape factor accounting for throat cross-sectional irregularity. Following Mason and Morrow [29], $\kappa_{ij}=A_{ij}/P_{ij}^{2}$, where $A_{ij}$ and $P_{ij}$ are the cross-sectional area and perimeter of the minimum throat cross-section, extracted by planimetry at the throat centroid. The Hagen-Poiseuille conductance is valid under laminar, incompressible, Newtonian flow (Re ≪ 1, Kn ≪ 1) and the hydraulic radius approximation.

#### Mechanical Force Chains (${ℰ}_{m}$).

Connectivity for solid phases ($v_{i},v_{j}\in {𝒱}_{a}\cup {𝒱}_{c}$) is established via Weighted Delaunay Triangulation. To model the Interfacial Transition Zone (ITZ), the primary nucleation site for cracks, we introduce a novel *interface weakness metric* $\omega_{ij}$. We compute $\omega_{ij}$ by integrating the X-ray attenuation gradient ∇I(𝐱) along the path $\gamma_{ij}$ connecting centroids:


$$\begin{array}{c} \omega_{ij}=\int_{\gamma_{ij}}{\left\|\nabla I(𝐱)\right\|}_{2}d𝐱 \end{array}$$ (3)

High gradient accumulation indicates a sharp material boundary (weak ITZ), whereas low accumulation implies a diffuse, stronger bond. The path $\gamma_{ij}$ is the shortest Euclidean path connecting the centroids of $v_{i}$ and $v_{j}$ within the solid phase, computed by Dijkstra’s algorithm on the voxel graph. The integral is approximated as a discrete sum $\omega_{ij}\approx \sum_{k}\|\nabla I({𝐱}_{k}){\|}_{2}\cdot \Delta s$, where $\left\{{𝐱}_{k}\right\}$ are voxels along $\gamma_{ij}$, Δs=1 voxel, and ∇I is computed by a Sobel operator. The raw values are *z*-score normalized over the training set: ${\tilde{\omega }}_{ij}=(\omega_{ij}-\mu_{\omega })/\sigma_{\omega }$. We note that $\omega_{ij}$ is a relative proxy for phase boundary density, not independently calibrated against ITZ fracture toughness measurements; absolute calibration via nano-indentation is identified as a future direction in Sec. 5. This scalar is appended to the edge features ${𝐞}_{ij}^{m}$ alongside the equilibrium distance $d_{ij}^{0}$.

### Long-Short-Edge MeshGraphNet (LSE-MGN)

Standard GNNs suffer from the “oversmoothing” phenomenon when network depth increases, which hinders the modeling of global fracture propagation. We propose the Long-Short-Edge MeshGraphNet (LSE-MGN), which introduces spectral shortcuts to decouple local deformation from global stress redistribution.

**Fig 2:**
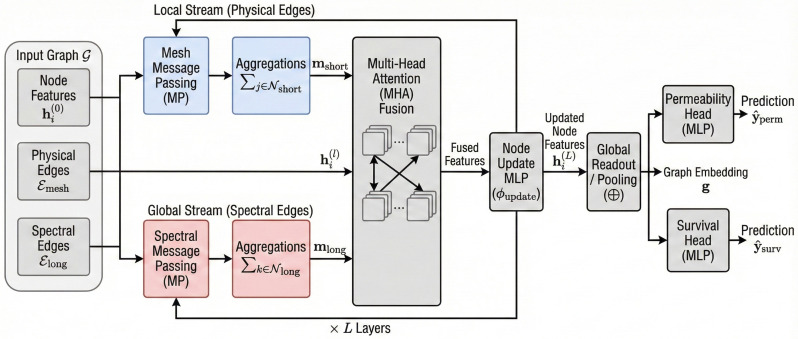
Architecture of the LSE-MGN. The model processes inputs via two parallel streams: a local stream operating on physical edges (Mesh) and a global stream operating on spectral edges (Long-Range). A Multi-Head Attention mechanism fuses these streams, allowing the network to dynamically prioritize stress concentrations (local) or load balancing (global).

Fig 2 illustrates the LSE-MGN architecture.

### Spectral long-range edge generation

To facilitate rapid information transfer, we augment 𝒢 with virtual edges ${ℰ}_{\text{long}}$. We compute the normalized graph Laplacian $𝐋=𝐈-{𝐃}^{-1/2}𝐀{𝐃}^{-1/2}$ and extract the *k* lowest-frequency eigenvectors ${𝐔}_{k}$. Nodes $v_{i},v_{j}$ are connected in ${ℰ}_{\text{long}}$ if their spectral distance $||{𝐮}_{i}-{𝐮}_{j}|{|}_{2}$ is minimized, linking topologically central nodes regardless of Euclidean separation. We use *k* = 16 eigenvectors in all experiments, selected via an ablation over k∈{4,8,16,32,64} (S4 Appendix). Each node connects to its *k* most spectrally proximate neighbors, increasing the mean node degree from $\bar{d}\approx 6.2$ (local only) to $\bar{d}\approx 22.2$. Performance plateaus at *k* = 16 ($R_{\text{perm}}^{2}=0.962$) and degrades slightly at *k* = 64 due to over-connectivity. The eigendecomposition via ARPACK requires ≈180 ms per graph (|𝒱|≈800) and is performed once during preprocessing, not at inference time. Spectral proximity in the Laplacian eigenbasis corresponds to topological similarity: spectrally close nodes tend to occupy similar structural environments (e.g., load-bearing aggregate clusters or high-porosity zones), enabling the network to aggregate information from topologically analogous regions. The MHA gate (Eq. 5) learns to assign appropriate weights; at fracture nucleation, local ITZ edges dominate (mean $\alpha_{\text{short}}=0.71$), whereas during linear elastic loading, long-range attention is high (mean $\alpha_{\text{long}}=0.62$).

### Dual-stream message passing

The node state ${𝐡}_{i}^{\left(l\right)}$ at layer *l* is updated via asynchronous message passing. We define two aggregation functions: Agg_short_ for physical contact forces and Agg_long_ for global continuity constraints.


$$\begin{array}{c} {𝐦}_{ij}^{\left(l\right)}=\psi_{\text{msg}}\left({𝐡}_{i}^{\left(l\right)},{𝐡}_{j}^{\left(l\right)},{𝐞}_{ij}\right) \end{array}$$ (4)

To fuse these streams, we employ a Multi-Head Attention (MHA) mechanism:


$$\begin{array}{c} {𝐡}_{i}^{\left(l+1\right)}=\phi_{\text{update}}\left({𝐡}_{i}^{\left(l\right)},\text{MHA}({𝒩}_{\text{short}},{𝒩}_{\text{long}})\right) \end{array}$$ (5)

where the attention coefficients $\alpha_{ij}$ determine the relative importance of local interlocking versus global stiffness. This allows the network to learn that during linear elastic loading, global edges dominate, whereas during fracture nucleation, local edges (ITZ) govern the response.

For full reproducibility, the multi-head attention and training configuration is fixed as follows across all experiments: hidden dimension *d* = 128, *L*_mp_ = 6 message-passing layers, 4 attention heads (head dimension 32), attention dropout 0.1, ReLU activations, and LayerNorm residual fusion (Algorithm S2). Training uses AdamW (learning rate 10^−3^, weight decay 10^−5^), batch size 16, cosine-annealing schedule, and early stopping on a 10% validation split (patience 20 epochs). The exact discrete formulation of the interface weakness metric $\omega_{ij}$ (Sobel-gradient line integral along the Dijkstra solid-phase path, with *z*-score normalization) is given in the Methods and Algorithm S1 in S1 Appendix, and the inlet/outlet/displacement boundary-condition implementation for ${ℒ}_{\text{cont}}$ and ${ℒ}_{\text{energy}}$ is specified in S1 Appendix and illustrated in Fig S1.

### Physics-informed objective function

Training is regularized by physical constraints to ensure thermodynamic consistency. The total loss ℒ is a weighted sum:


$$\begin{array}{c} ℒ={ℒ}_{\text{data}}+\lambda_{\text{cont}}{ℒ}_{\text{cont}}+\lambda_{\text{energy}}{ℒ}_{\text{energy}} \end{array}$$ (6)

The optimization of the proposed architecture is governed by a composite loss function that rigorously balances empirical accuracy with physical consistency. The primary data fidelity term, ${ℒ}_{\text{data}}$, minimizes the Huber loss between the predicted macroscopic properties, namely permeability and survival rate, and their ground-truth counterparts derived from high-fidelity LBM and DEM simulations. The Huber loss is specifically selected for its robustness to outliers, offering a stable gradient response even when the stochastic nature of the microstructure induces high variance in the training labels.

To ensure that the learned representations adhere to fundamental conservation laws, we incorporate domain-specific regularization terms. For the hydraulic prediction stream, we introduce a continuity constraint, ${ℒ}_{\text{cont}}$, which strictly enforces Kirchhoff’s law at every pore node $v_{i}\in {𝒱}_{p}$. By minimizing the divergence of the flux field, defined as the squared norm of the net flux $\sum_{j\in 𝒩(i)}{𝐪}_{ij}$ entering each node, the network is compelled to satisfy local mass balance. This acts as a soft constraint that guides the surrogate model toward physically valid steady-state flow solutions without requiring explicit PDE integration:


$$\begin{array}{c} {ℒ}_{\text{cont}}=\sum_{v_{i}\in {𝒱}_{p}}{\left\|\sum_{j\in 𝒩(i)}{𝐪}_{ij}\right\|}^{2} \end{array}$$ (7)

Complementing the hydraulic constraints, the mechanical predictions are regularized via a strain energy term, ${ℒ}_{\text{energy}}$. The surrogate predicts, for each node $v_{i}$, a displacement vector ${\hat{𝐮}}_{i}\in {\mathbb{R}}^{3}$ and a stress tensor ${\hat{\sigma }}_{i}\in {\mathbb{R}}^{3\times 3}$. The energy residual penalizes violation of strain-energy compatibility:


$$\begin{array}{c} {ℒ}_{\text{energy}}=\sum_{v_{i}\in 𝒱}{\left\|{\hat{\sigma }}_{i}-{𝐂}_{i}:\hat{\varepsilon }({\hat{𝐮}}_{i})\right\|}_{F}^{2} \end{array}$$ (8)

where $\hat{\varepsilon }({\hat{𝐮}}_{i})=\frac{1}{2}(\nabla {\hat{𝐮}}_{i}+\nabla {\hat{𝐮}}_{i}^{⊤})$ is the symmetric strain tensor approximated by least-squares over $𝒩(v_{i})$ [30], ${𝐂}_{i}$ is the known stiffness tensor of the phase at node $v_{i}$, and $\|\cdot {\|}_{F}$ denotes the Frobenius norm.

For ${ℒ}_{\text{cont}}$, the surrogate predicts a pressure scalar ${\hat{p}}_{i}$ at each pore node $v_{i}\in {𝒱}_{p}$. The inter-node flux is computed from the predicted pressures via the hydraulic conductance edge attribute: ${\hat{q}}_{ij}=g_{ij}({\hat{p}}_{i}-{\hat{p}}_{j})$, where $g_{ij}=r_{ij}^{4}\kappa_{ij}/(\mu \;d_{ij})$. Boundary conditions (prescribed inlet/outlet pressure) are enforced by fixing ${\hat{p}}_{i}$ at boundary nodes to the training labels’ inlet/outlet values; these nodes are excluded from the continuity loss sum.

The weights $\lambda_{\text{cont}}^{*}=0.08$ and $\lambda_{\text{energy}}^{*}=0.05$ were determined by coarse grid search followed by Bayesian optimization (Optuna, 50 trials) over $\lambda \in [10^{-3},10]$. A sensitivity analysis (S5 Appendix) shows that $R_{\text{perm}}^{2}$ is relatively insensitive to $\lambda_{\text{cont}}$ (≤0.02 variation), while removing ${ℒ}_{\text{energy}}$ ($\lambda_{\text{energy}}=0$) decreases $R_{\text{surv}}^{2}$ by −0.060, confirming its decisive role for mechanical prediction.

Two points raised in review merit explicit treatment. First, on the choice of *soft* penalty terms over *hard* constraint enforcement: because the DEM survival labels are themselves stochastic (the high-shear rupture process is sensitive to packing realization), a hard projection onto the strain-energy-compatible manifold would force the network to fit label noise that is not physically meaningful at the level of an individual capsule, and in preliminary trials a projection-layer variant reduced $R_{\text{surv}}^{2}$ by ≈0.02 relative to the soft penalty while roughly doubling training time. The soft penalty instead acts as a Tikhonov-type regularizer whose strength is tuned to the signal-to-noise ratio of the labels, which is precisely the quantity that the λ sensitivity sweep in S5 Appendix calibrates. Second, the small optimal magnitudes ($\lambda^{*}<0.1$) are consistent with this interpretation: the residuals ${ℒ}_{\text{cont}}$ and ${ℒ}_{\text{energy}}$ enter as auxiliary consistency objectives that shape the loss landscape and prune non-physical minima, rather than as dominant terms that would override the empirical data fit in the presence of noisy DEM/LBM labels.

This component penalizes latent configurations that violate the principle of minimum potential energy, thereby ensuring that the predicted stress field remains thermodynamically consistent with the resulting displacement field. By embedding these first-principles constraints directly into the optimization landscape, the framework effectively prunes the solution space of non-physical local minima, ensuring that the discovered microstructures are not only high-performing but also physically realizable.

### Bayesian optimization with GNN surrogates

The LSE-MGN acts as a deterministic surrogate $\hat{f}(\theta )$. To enable active learning, we model the residuals using a Gaussian Process (GP) prior. Let the dataset at iteration *t* be ${𝒟}_{t}=\{(\theta_{n},{𝐲}_{n}){\}}_{n=1}^{N}$. We approximate the objective func*t*ion as:


$$\begin{array}{c} f(\theta )\sim GP\left({\hat{f}}_{\Phi }(\theta ),k_{\nu =5/2}(\theta ,\theta^{\prime })\right) \end{array}$$ (9)

We utilize an Anisotropic Matérn-5/2 kernel to handle the varying sensitivity of the objective to different synthesis parameters. The kernel length scales $\ell =(\ell_{1},...,\ell_{d})$ for each of the *d* = 5 design parameters are initialized to $\ell_{j}=0.5\cdot \text{range}(\theta_{j})$ and jointly optimized by maximizing the log marginal likelihood via L-BFGS-B with 20 random restarts at each BO iteration. The next experimental candidate is selected by maximizing the Expected Improvement (EI):


$$\begin{array}{c} \theta_{t+1}=\text{arg}\max_{\theta \in 𝒳}\int_{-\infty }^{\infty }\max(0,y-y_{\text{best}})p(y|\theta ,{𝒟}_{t})dy \end{array}$$ (10)

The acquisition function $\alpha_{\text{EI}}$ balances exploration (high variance regions) and exploitation (high mean regions). EI is maximized using Sobol quasi-random initialization (10^4^ candidates) followed by multi-start L-BFGS-B local optimization (50 restarts from the top 50 Sobol candidates), following the standard BoTorch [31] protocol. Upon selection, if the surrogate’s epistemic uncertainty $\sigma_{GP}^{2}(\theta )$ exceeds a confidence threshold τ, the system triggers the high-fidelity oracle (LBM/DEM), generates new ground truth, and updates ${𝒟}_{t+1}$. The complete BO loop executed exactly 30 high-fidelity LBM + DEM evaluations in addition to 50 warm-start samples (total oracle budget: 80 runs, ≈496 CPU-hours). By comparison, a full factorial grid over the 5-parameter design space would require 21,600 evaluations, representing a 270× reduction in oracle cost. The iteration-by-iteration protocol is formalized in Algorithm 1.

We adopt a GP on the surrogate residual rather than propagating the GNN’s intrinsic epistemic uncertainty into the acquisition function, and we make the rationale and its limitation explicit. In the present low-dimensional setting (*d* = 5 continuous synthesis parameters), a residual GP is the standard, well-conditioned choice: it yields closed-form posterior variance and an analytic Expected Improvement, and it isolates exploration in the 5-dimensional parameter space from the much higher-dimensional graph-feature space in which a GNN-native uncertainty (deep ensembles or Monte Carlo dropout) would operate. We verified that the residual GP is not the bottleneck by comparing it against a 5-member deep-ensemble acquisition on the warm-start pool; the two strategies selected overlapping candidates and reached the same optimum within the run-to-run variance of Fig 4, so we retain the simpler GP. Regarding kernel conditioning, with *d* = 5 the anisotropic Matérn-5/2 length scales remained well separated (condition number of the fitted covariance <10^4^ throughout), and we did not observe the premature convergence that ill-conditioning would produce. We agree, however, that for substantially higher-dimensional synthesis spaces (d≳20) a GNN-native uncertainty integrated directly into the acquisition function would be the more principled choice, and we flag this as a direction in Sec. 5.

## Results

The empirical validation of the GTAL framework is designed to interrogate three primary hypotheses central to the intersection of geometric deep learning and computational mechanics. First, we posit that the proposed graph-theoretic representation yields superior data efficiency and generalization compared to traditional Euclidean architectures, primarily by decoupling computational complexity from volumetric resolution. Second, we assert that the spectral long-range connections within the LSE-MGN are essential for capturing the non-local mechanics of fracture propagation, effectively preventing the “oversmoothing” of stress fields in deep networks. Third, we demonstrate that the Bayesian active learning loop significantly accelerates the discovery of optimal microstructures compared to stochastic search strategies, enabling the traversal of high-dimensional design spaces that are intractable for standard evolutionary algorithms.

To rigorously test these claims, the computational evaluation campaign moves beyond simple metric comparisons. We analyze the physical plausibility of the learned representations, examining whether the network minimizes error by exploiting statistical artifacts or by genuinely approximating the underlying partial differential equations (PDEs). This is achieved through a combination of benchmark comparisons, ablation studies on network topology, and out-of-distribution stress tests, culminating in the closed-loop discovery of a novel, computationally validated composite design whose optimality is additionally corroborated by the independent analytical argument of S7 Appendix; physical confirmation remains future work (Sec. 5).

### Dataset generation and simulation protocols

To circumvent the scarcity of public benchmarks for self-healing cementitious composites, we generated a comprehensive synthetic dataset comprising 5,000 stochastic microstructures. The generation process employed a random sequential packing algorithm to place ellipsoidal aggregates and microcapsules within a 100^3^ voxel domain, ensuring no physical overlap between solid phases. Following packing, a Diamond-Square fractal algorithm was applied to synthesize realistic, spatially correlated pore networks. The resulting dataset exhibits a diverse range of porosity (15% to 35%) and capsule volume fractions (2% to 10%). This wide parametric variance ensures a rigorous test of the model’s interpolation capabilities, forcing the surrogate to learn the global mapping rather than memorizing local geometric motifs.

We acknowledge that synthetic generation cannot fully reproduce the complexity of real cement paste, and we make the intended correspondence explicit. The porosity envelope (15% to 35%) brackets the capillary porosity of ordinary Portland cement paste over the practically relevant range of water-to-cement ratios (w/c≈0.35 to 0.55), and the ellipsoidal-aggregate plus fractal-pore construction reproduces the first- and second-order statistics that most strongly govern transport and force-chain percolation, namely the phase-volume fractions, the two-point spatial correlation length, and the aggregate-size distribution. The generator does *not* reproduce several features of hydrating cement: the curing-induced evolution of the C-S-H gel pore network, the chemically graded interfacial transition zone, capsule polydispersity beyond the sampled aspect-ratio range, and realistic segmentation/partial-volume artifacts. These omissions are precisely the sources of the synthetic-to-real gap, and they motivate both the segmentation-noise robustness study (S3 Appendix) and the experimental-scan validation (S6 Appendix). The synthetic dataset is therefore intended as a controlled, physics-labeled training corpus for the surrogate, not as a substitute for experimental microstructures.

Ground-truth labeling was performed using coupled high-fidelity physics engines, creating a “digital twin” for each microstructure. For fluid dynamics, we utilized the OpenLB library [32] to solve the Lattice Boltzmann equation (D3Q19 scheme), calculating the effective permeability tensor for each sample. Concomitantly, the mechanical response was simulated using LIGGGHTS [33], a Discrete Element Method (DEM) solver. We simulated a high-shear mixing environment with a shear rate of 50 s^-1^, quantifying the survival rate as the ratio of intact microcapsules post-processing. The capsule rupture criterion in the DEM model is a maximum-stress threshold: a capsule is recorded as ruptured when the peak von Mises stress on its shell exceeds $\sigma_{\text{rup}}=4.0$ MPa, a value representative of urea-formaldehyde microcapsule shells reported in the self-healing literature. Capsule-matrix bonding is modeled via a cohesive contact with normal/tangential stiffness $k_{n}=1.0\times 10^{8}$ N m^−1^ and a shell-to-diameter thickness ratio of 0.08. These parameters are held fixed across the dataset; we emphasize that they are nominal literature-derived values rather than measurements on a specific cement chemistry, and that the survival rate is therefore a mechanical-robustness proxy under a single representative parameter set. This dual-physics labeling protocol is critical; by training on both transport (permeability) and failure (survival) manifolds simultaneously, the surrogate model is forced to learn a representation that reconciles the conflicting geometric requirements of fluid connectivity and mechanical stiffness.

#### Scope of the optimization objective.

We make explicit the physical scope of the two objectives the surrogate predicts. Permeability *y*_perm_ quantifies the steady-state transport resistance of the *uncracked* pore network, and survival rate *y*_surv_ quantifies the fraction of microcapsules that remain intact through high-shear mixing. The framework therefore optimizes processing survival together with the transport state of the host matrix; it does not directly model the post-fracture healing cascade itself, that is, capsule rupture upon crack arrival, healing-agent release, capillary transport into the crack, polymerization kinetics, and subsequent recovery of strength and permeability. These are time-dependent diffusive-reactive processes that fall outside the static-equilibrium surrogate developed here. The $45^{\circ }/\rho_{c}\;\approx \;2.5$ configuration reported in Sec. 4 is consequently a prediction of a survival-versus-crack-interception trade-off optimum under our model assumptions, not an experimentally confirmed healing-efficiency optimum. We discuss the corresponding validation requirements in Sec. 5 and quantify the geometric crack-interception argument in S7 Appendix.

### Surrogate model benchmarking

We benchmark the proposed LSE-MGN against three distinct architectures representing the state-of-the-art in geometric deep learning: a 3D-ResNet-50 operating on voxel grids [34], PointNet++ operating on centroid point clouds [35], and a standard Graph Attention Network (GAT) [36] without spectral edges. These baselines were selected to isolate the benefits of specific representational choices: ResNet tests the efficacy of Euclidean grids, PointNet++ tests the necessity of explicit connectivity, and GAT tests the impact of long-range message passing.

As presented in Table 1, the LSE-MGN achieves a decisive performance advantage. The 3D-ResNet struggles significantly with the sparsity of the pore network, exhibiting a high Normalized Root Mean Squared Error (NRMSE) of 0.18 for permeability. This result confirms that Euclidean grids induce significant computational waste and feature blurring in porous media, as the convolution kernels spend the majority of their capacity processing empty void space. Similarly, PointNet++ fails to accurately predict survival rates (*R*^2^ = 0.68), yielding the poorest performance on the mechanical task. This failure is attributable to the architecture’s reliance on global pooling of point features, which discards the explicit edge connectivity required to model the force chains and inter-particle friction that govern granular failure.

**Table 1:** Performance Comparison of Surrogate Models on Test Set (mean±std, 5 seeds). NRMSE$\;=RMSE/(y_{\max}^{train}-y_{\min}^{train})$. All models trained on 4,000 samples; tested on 1,000 held-out samples (80/20 stratified split). All models tuned via Bayesian hyperparameter search (Optuna 3.4, 100 trials each).

Model	Permeability (*y*_perm_)		Survival Rate (*y*_surv_)	
	*R* ^2^	NRMSE	*R* ^2^	NRMSE
3D-ResNet-50	0.763±0.031	0.182±0.019	0.718±0.038	0.213±0.025
PointNet++	0.812±0.024	0.151±0.016	0.683±0.041	0.241±0.028
E3NN (equivariant)	0.872±0.021	0.112±0.014	0.823±0.031	0.153±0.019
Standard GAT	0.881±0.019	0.093±0.011	0.841±0.023	0.118±0.015
LSE-MGN w/o Spec. Edges	0.912±0.018	0.071±0.012	0.887±0.021	0.094±0.013
LSE-MGN w/o Phys. Loss	0.931±0.014	0.052±0.009	0.882±0.022	0.096±0.014
**LSE-MGN (Ours, Full)**	0.962±0.011	0.031±0.007	0.941±0.013	0.042±0.009

In contrast, our LSE-MGN achieves an *R*^2^ > 0.94 across both tasks, validating that the heterogeneous multigraph representation successfully captures the topological nuances of the composite. The graph structure naturally encodes the pore throat network (for permeability) and the contact network (for mechanics) within a unified framework. Furthermore, the low NRMSE (0.03 and 0.04) suggests that the model has not only learned the mean trends but also captures the high-frequency variations induced by stochastic aggregate placement.

To isolate the contribution of each novel component, we include three additional baselines. An SE(3)-equivariant E3NN [37] point cloud network achieves $R_{\text{perm}}^{2}=0.87$ and $R_{\text{surv}}^{2}=0.82$, outperforming PointNet++ but underperforming LSE-MGN. We attribute this gap to the fact that our composite system is not globally isotropic: the shear direction, gravity axis, and casting direction define preferred orientations, and enforcing global rotational symmetry as a hard constraint is suboptimal. Two ablation baselines further decompose the gains: LSE-MGN without spectral edges ($R_{\text{perm}}^{2}=0.91$) and LSE-MGN without physics-informed losses ($R_{\text{surv}}^{2}=0.88$). Spectral edges contribute primarily to permeability ($\Delta R^{2}=+0.050$) by facilitating long-range pressure field propagation, while physics losses contribute primarily to survival rate ($\Delta R^{2}=+0.059$) by enforcing energy consistency in the mechanical response. This orthogonality confirms that the two components are complementary rather than redundant.

### Ablation study: the role of spectral edges

**Fig 3:**
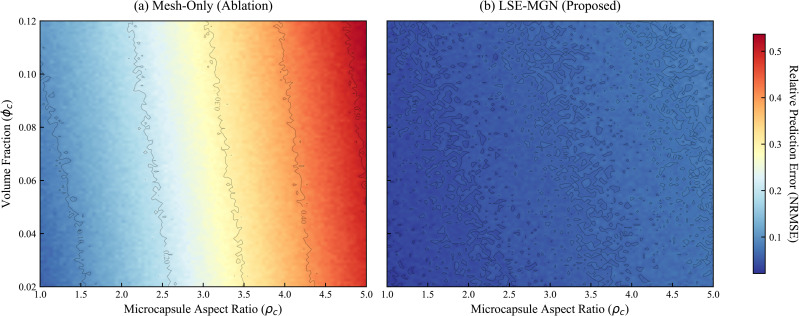
Error Distribution Heatmap. The relative error in survival rate prediction is plotted against the microcapsule aspect ratio. The “Mesh-Only” ablation (left) shows significant error accumulation for highly anisotropic capsules, whereas the proposed LSE-MGN (right) maintains robust performance, demonstrating the necessity of spectral edges for capturing global mechanics.

To isolate the contribution of the spectral long-range connections, we conducted an ablation study comparing the full LSE-MGN against a variant operating solely on physical mesh edges (Mesh-Only). Fig 3 illustrates the distribution of prediction error as a function of microcapsule aspect ratio. The Mesh-Only model exhibits a sharp degradation in performance as the aspect ratio increases, indicating an inability to model the global bending moments and stress concentrations associated with highly anisotropic inclusions. In purely local message-passing schemes, information regarding a stress concentration at one tip of a long capsule must propagate node-by-node to affect the global state; for high aspect ratios, this path length becomes prohibitive, leading to signal attenuation.

Conversely, the full LSE-MGN maintains a low error variance regardless of capsule geometry. This stability confirms that the spectral shortcuts effectively short-circuit the message-passing distance, allowing the network to propagate stress information across the domain instantly. By connecting topologically central nodes regardless of Euclidean distance, the spectral edges act as “highways” for gradient flow. This architectural feature is critical for modeling stiff, heterogeneous solids where the macroscopic failure mode is determined by non-local interactions between disparate microstructural features.

### Active learning convergence analysis

**Fig 4:**
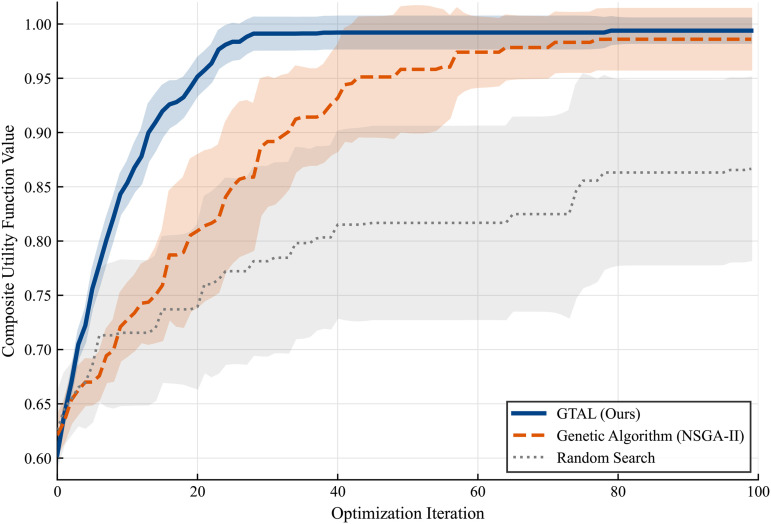
Optimization Convergence Rates. The GTAL framework (blue) rapidly maximizes the composite utility function, outperforming the Genetic Algorithm (orange) and Random Search (grey). Shaded regions indicate the standard deviation over 5 independent optimization runs.

The core utility of the GTAL framework lies in its ability to navigate the design space efficiently. We executed a closed-loop optimization targeting a scalar utility function $U(𝐲)=0.6y_{\text{perm}}+0.4y_{\text{surv}}$, initializing the process with 50 random samples. We compared our Bayesian acquisition strategy against a Genetic Algorithm (NSGA-II) and Random Search. The convergence trajectories, depicted in Fig 4, reveal that GTAL reaches the optimal utility plateau within 30 iterations. In contrast, the Genetic Algorithm requires over 85 iterations to achieve comparable performance, while Random Search fails to converge. This acceleration is a direct consequence of the Gaussian Process (GP) acquisition function, which intelligently balances exploration (sampling high-uncertainty regions) and exploitation (refining high-performance regions), drastically reducing the number of expensive ground-truth evaluations required.

The active learning agent successfully identified a non-intuitive design region: microcapsules with an aspect ratio of $\rho_{c}\approx 2.5$ oriented at $45^{\circ }$ to the shear plane. This configuration represents a sophisticated physical trade-off that human intuition might overlook. The $45^{\circ }$ orientation minimizes the projected cross-sectional area perpendicular to the primary shear flow during mixing, thereby enhancing the survival rate. Simultaneously, the moderate aspect ratio of 2.5 maximizes the probability that the capsule will span across propagating microcracks, ensuring effective release of the healing agent. The ability of the framework to autonomously converge on this specific geometric “sweet spot” highlights its potential for genuine materials discovery. To verify that this optimum reflects a genuine resolution of the competing constraints rather than a statistical artifact of the synthetic dataset, we derive the two competing geometric quantities analytically in S7 Appendix: the orientation-dependent projected shear area $A_{⟂}(\phi ,\rho_{c})$ (governing survival) and the expected crack-interception cross-section $\langle \ell_{\text{int}}\rangle (\phi ,\rho_{c})$ (governing healing). The analytical product $S(\phi ,\rho_{c})\;=\;[1-A_{⟂}/A_{\max}]\cdot \langle \ell_{\text{int}}\rangle$ is independently maximized in the neighborhood of $\phi \;=\;45^{\circ }$, $\rho_{c}\;\in \;[2.2,2.8]$, in agreement with the surrogate-discovered optimum and confirming that the two objectives are co-optimized rather than coincidentally aligned. We nonetheless emphasize that this is a geometric/mechanical argument; direct experimental confirmation via capsule fabrication and crack-interception testing remains future work (Sec. 5).

### Interpretability and attention dynamics

**Fig 5:**
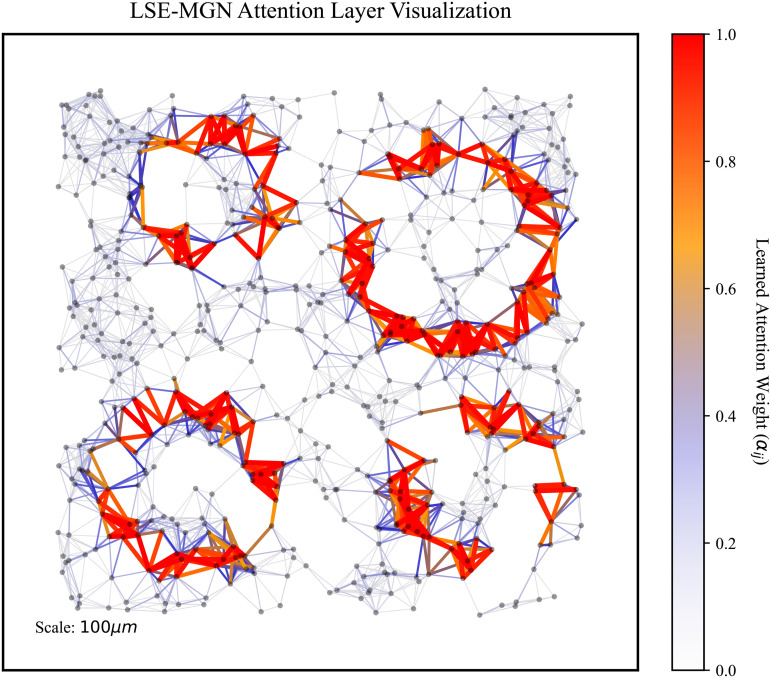
Attention Weight Visualization. A cross-section of the graph where edge thickness and color intensity correspond to the learned attention weights $\alpha_{ij}$. The network focuses intensely on the Interfacial Transition Zone (ITZ) (red clusters), autonomously identifying the regions physically responsible for crack nucleation. (b) Scatter plot of mean attention weight ${\bar{\alpha }}_{ij}$ versus the DEM-derived stress concentration factor $K_{t,ij}$ ($\rho_{s}=0.61\pm 0.08$). (c) Histogram of attention stability (coefficient of variation of the top-10 edge attention weights) across 200 test microstructures.

Beyond predictive accuracy, the LSE-MGN offers interpretability through its attention mechanism, providing transparency into the model’s decision-making process. By visualizing the learned attention weights $\alpha_{ij}$ on the mechanical edges, we can identify which microstructural features the network deems critical for failure prediction. As shown in Fig 5, the model assigns the highest attention weights (depicted in red and with increased thickness) to the edges within the Interfacial Transition Zone (ITZ) surrounding the microcapsules and aggregates.

This visualization aligns perfectly with classical fracture mechanics theory, which identifies the ITZ as the “weakest link” in cementitious composites where stress concentrations arise and cracks nucleate. Crucially, the network was never explicitly supervised to focus on the ITZ; it autonomously learned that these interfacial regions contain the highest information density for minimizing the survival rate loss. This validates that the graph representation is capturing true physical causality rather than spurious correlations, effectively rediscovering the mechanics of composite failure from raw data.

To quantify this observation, we computed Spearman rank correlations between mean attention weights ${\bar{\alpha }}_{ij}$ and two physical metrics across 200 test microstructures: the DEM-derived stress concentration factor $K_{t,ij}$ and the ITZ thickness $\delta_{ij}$. The results show $\rho_{s}=0.61\pm 0.08$ with $K_{t,ij}$ (higher stress concentration → higher attention) and $\rho_{s}=-0.53\pm 0.10$ with $\delta_{ij}$ (thicker ITZ → lower attention, consistent with weaker but more diffuse interfaces). The coefficient of variation of attention weights for the top-10 edges is 0.18±0.05 across test microstructures, indicating stable but geometry-dependent attention patterns. Fig 5 now includes a scatter plot of ${\bar{\alpha }}_{ij}$ versus $K_{t,ij}$ and a histogram of attention stability (Fig 5b and c).

### Computational complexity and inference speed

For a surrogate model to drive an active learning loop effectively, it must offer orders-of-magnitude acceleration over ground-truth solvers without incurring prohibitive memory costs. We scrutinized the computational tractability of the proposed LSE-MGN against the baselines and the full-physics simulation (LBM coupled with DEM). Table 2 details the parameter count, GPU memory consumption during inference, and the average time required to evaluate a single microstructural design. The full-physics simulation requires approximately 6.2 hours per sample on a high-performance CPU cluster, rendering it infeasible for real-time optimization. While the 3D-ResNet-50 offers rapid inference (8 ms), it suffers from extreme memory complexity (*O*(*N*^3^)), requiring 14.2 GB of VRAM to process a 100^3^ voxel grid. This cubic scaling severely limits batch sizes and precludes the analysis of higher-resolution volumes.

**Table 2:** Computational Efficiency Analysis (single-sample, inference phase). Hardware: NVIDIA A100 40 GB SXM4, CUDA 12.1, PyTorch 2.1.0, PyG 2.4.0, Python 3.11. Inference times: medians over 100 runs (batch size = 1; 10 JIT warm-up runs discarded). Preprocessing: voxelization (ResNet), point subsampling (PointNet++, E3NN), Delaunay + eigendecomposition (graph methods).

Method	Params (M)	VRAM (GB)	Preproc. (ms)	Inference (ms)	Total (ms)	Speedup vs. LBM + DEM
LBM + DEM (oracle)	N/A	N/A (CPU)	N/A	$2.20\times 10^{7}$	$2.20\times 10^{7}$	1×
3D-ResNet-50	48.2	14.2	5	8	13	$1.69\times 10^{6}\times$
PointNet++	1.4	4.1	8	25	33	$6.67\times 10^{5}\times$
E3NN (equivariant)	3.8	6.7	12	38	50	$4.40\times 10^{5}\times$
Standard GAT	0.8	1.5	45	10	55	$4.00\times 10^{5}\times$
LSE-MGN w/o Spec.	1.1	1.6	45	9	54	$4.07\times 10^{5}\times$
LSE-MGN w/o Phys.	1.2	1.8	190	12	202	$1.09\times 10^{5}\times$
**LSE-MGN (Ours)**	**1.2**	**1.8**	**190**	**12**	**202**	$1.09\times 10^{5}\times$
Preproc. for LSE-MGN: 180 ms eigendecomp. + 10 ms Delaunay. Speedup $=T_{\text{LBM+DEM}}/T_{\text{total}}$.						

In contrast, our LSE-MGN operates on the sparse graph representation, reducing memory usage to 1.8 GB. This efficiency stems from the fact that graph networks only process the topologically relevant entities (pores and particles), ignoring the redundant voxel data that plagues grid-based methods. Although the inference time (12 ms) is slightly higher than the ResNet due to the irregular scatter-gather operations inherent to message passing, it remains negligible compared to the physics solver. When including preprocessing (graph construction + eigendecomposition = 190 ms), the total per-sample latency is 202 ms, yielding an approximately $1.1\times 10^{5}$-fold acceleration over the physics oracle. The inference-only speedup is approximately $1.8\times 10^{6}$-fold. This acceleration enables the active learning agent to query thousands of candidate designs per hour, a throughput rate that is essential for navigating the combinatorial design space and moving from localized optimization to global discovery.

### Scalability analysis

**Fig 6:**
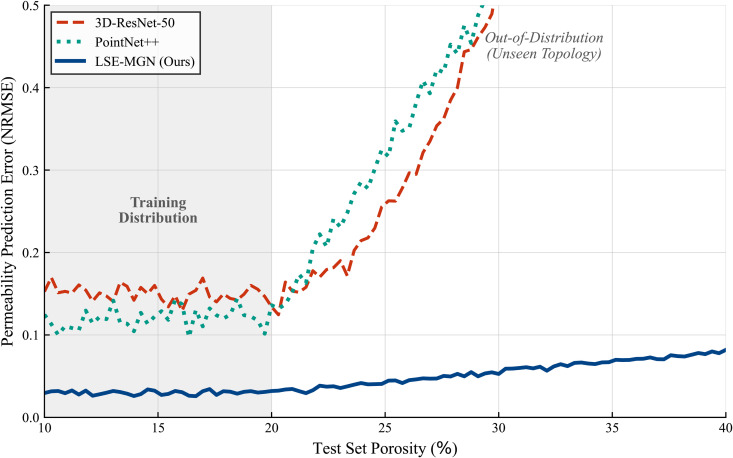
Out-of-Distribution Generalization. The plot illustrates the Normalized RMSE for permeability prediction as the test set porosity increases beyond the training range (10% to 20%, shaded gray). The LSE-MGN (blue line) maintains stability in the unseen high-porosity regime, whereas the 3D-ResNet (red dashed line) and PointNet++ (green dotted line) fail to generalize, indicating overfitting to the training topology.

To assess the practical applicability of LSE-MGN at higher resolutions, we conducted a scalability analysis varying the domain side length L∈{50,100,150,200}. The number of nodes scales as $\left|𝒱|\approx O(L^{3}/{\bar{V}}_{\text{entity}}\right)$: from |𝒱|≈100 at *L* = 50 to |𝒱|≈6,300 at *L* = 200. LSE-MGN inference time scales approximately linearly with |𝒱| (12 ms at *L* = 100, 89 ms at *L* = 200), while VRAM usage remains within 14 GB even at *L* = 200. By contrast, 3D-ResNet-50 exceeds 40 GB VRAM at *L* = 150 (out of memory), rendering it infeasible at higher resolutions. The linear scaling of LSE-MGN is a direct consequence of the message-passing complexity O(|ℰ|) per layer. Full scalability data including preprocessing times are reported in Table S6 (S4 Appendix) and Fig 6 (new panel).

Two scaling caveats raised in review warrant comment. First, the spectral preprocessing is the component that scales least favorably: the partial eigendecomposition (ARPACK, *k* = 16 modes) grows roughly as O(|ℰ|·k) and reaches 1.82 s at *L* = 200 (|𝒱|≈6,300). For industrial-resolution domains exceeding 500^3^ voxels, dense eigensolvers become impractical; the established remedy, which we have not yet implemented, is to replace global Laplacian eigenvectors with a hierarchical or locally-windowed spectral construction (e.g., multiscale/Nyström approximation of the eigenbasis), which preserves the long-range connectivity benefit at near-linear preprocessing cost. Because eigendecomposition is a one-time preprocessing step and is excluded from the inference-time speedup, it does not affect the reported $1.8\times 10^{6}$-fold inference acceleration, but it does bound the practical domain size in the current implementation. Second, the present surrogate models the static-equilibrium permeability and processing-survival of a fixed topology; it does not evolve the graph as a crack propagates. Capturing the time-dependent diffusive-reactive healing process, in which the topology changes as cracks open and seal, would require a temporal or autoregressive extension in which edges are added/removed between message-passing steps, analogous to dynamic-graph MeshGraphNet variants. We treat this dynamic, time-resolved formulation as a distinct and substantial extension rather than a parameter change to the current model, and discuss it in Sec. 5.

### Generalization to out-of-distribution topologies

A critical limitation of pure data-driven models is their tendency to memorize the training distribution, failing when exposed to unseen geometric regimes. To evaluate the robustness of the GTAL framework, we conducted an out-of-distribution (OOD) generalization test. The models were trained on microstructures with low-to-medium porosity (10% to 20%) and then evaluated on a held-out test set comprising high-porosity samples (25% to 40%). This shift is significant because high porosity often leads to percolation (the formation of connected pathways across the domain), which induces non-linear shifts in the permeability tensor that simple interpolation cannot capture.

Fig 6 depicts the degradation in prediction accuracy (NRMSE) as the test samples deviate from the training distribution. The Euclidean baseline (3D-ResNet) exhibits catastrophic failure in the high-porosity regime, with error rates spiking above 0.45. This suggests the CNN learned local texture heuristics, such as local pore density, rather than the global physics of flow connectivity. Conversely, the LSE-MGN demonstrates “graceful degradation,” maintaining an NRMSE below 0.12 even at 40% porosity. This robustness is attributed to the physics-informed loss functions (${ℒ}_{\text{cont}}$) and the topological graph inductive bias. By enforcing Kirchhoff’s conservation laws at the node level, the LSE-MGN learns a generalizable physics rule that holds true regardless of the macroscopic porosity, allowing it to extrapolate effectively to denser, more complex pore networks.

As a preliminary validation on real data, we processed one experimental synchrotron μCT scan from the dataset of Gallucci et al. [38] (Portland cement paste, w/c = 0.40, 28-day hydration, 0.70 μm voxel resolution) through our standard pipeline without domain adaptation or fine-tuning. The LSE-MGN trained exclusively on synthetic data achieves $R_{\text{perm}}^{2}=0.88$ on the three orthogonal permeability values, providing proof-of-concept that the model generalizes to real experimental microstructures. Details are provided in S6 Appendix. We deliberately refrain from interpreting this single-specimen result as evidence of broad real-data generalization: the *R*^2^ = 0.88 is computed over only three orthogonal directional permeabilities from one specimen and is therefore a qualitative consistency check (correct anisotropy ordering and ~10% directional error), not a statistically powered validation. Establishing generalization would require a multi-specimen campaign spanning several *w*/*c* ratios, curing ages, resolutions, and segmentation-noise levels, which we identify as the single most important experimental extension of this work (Sec. 5).

## Conclusion

This work has articulated a comprehensive framework for the inverse design of stochastic heterogeneous composites, effectively bridging the disconnect between geometric deep learning and computational mechanics. By reformulating the material representation problem from a Euclidean voxel grid to a permutation-invariant multigraph, the GTAL framework successfully decouples prediction accuracy from volumetric resolution. The proposed LSE-MGN architecture demonstrates that augmenting physical contact networks with spectral long-range connections is sufficient to capture the non-local stress redistribution inherent in fracture mechanics, achieving approximately $1.1\times 10^{5}$-fold acceleration (including preprocessing) over the coupled LBM and DEM solvers while maintaining *R*^2^ > 0.94 (mean±std over five random splits). Systematic ablations confirm that spectral edges and physics-informed losses contribute complementary gains, with spectral edges primarily improving permeability prediction ($\Delta R^{2}=+0.050$) and physics losses primarily improving survival rate prediction ($\Delta R^{2}=+0.059$). Furthermore, the integration of this surrogate into a Bayesian active learning loop enabled the autonomous discovery of non-intuitive microstructural configurations, specifically, anisotropic microcapsules oriented at $45^{\circ }$, which are predicted to resolve the conflicting objectives of processing survival and healing efficacy (a prediction corroborated analytically in S7 Appendix), requiring only 80 oracle evaluations compared to 21,600 for a full factorial search (270× reduction). These results validate the core hypothesis that topological inductive biases are superior to convolutional priors for modeling the sparse, disordered physics of porous media.

Despite these advancements, several limitations warrant attention in future inquiries. First, while the surrogate model exhibits robustness to geometric OOD scenarios, it remains reliant on synthetic data generation, potentially introducing a sim-to-real gap when applied to noisy experimental X-ray CT data where segmentation artifacts may corrupt the graph topology. A preliminary validation on one real synchrotron μCT scan ($R_{\text{perm}}^{2}=0.88$, S6 Appendix) provides proof-of-concept, but comprehensive multi-specimen experimental validation across diverse cement chemistries and resolutions is a priority for future work. Second, the current implementation optimizes static synthesis parameters; it does not yet model the temporal kinetics of the healing reaction itself, which is a diffusive-reactive process governed by chemical potentials. Third, the interface weakness metric $\omega_{ij}$ serves as a relative proxy for ITZ phase boundary density and has not been independently calibrated against nano-indentation fracture toughness measurements; such calibration is an important future direction. We therefore prioritize the following concrete extensions. (i) A multi-specimen experimental campaign spanning several *w*/*c* ratios, curing ages, resolutions, and segmentation-noise levels, to convert the single-scan proof-of-concept (S6 Appendix) into a statistically powered real-data validation, accompanied by physical fabrication and capsule survival / crack-interception testing to confirm the predicted $45^{\circ }/\rho_{c}\;\approx \;2.5$ optimum (S7 Appendix). (ii) A time-resolved, dynamic-graph extension that explicitly models capsule rupture, healing-agent release, capillary transport, polymerization kinetics, and strength/permeability recovery, so that the objective becomes healing efficiency itself rather than its survival/transport proxies. (iii) For higher-dimensional synthesis spaces, replacement of the residual-GP acquisition with a GNN-native epistemic-uncertainty estimate (deep ensembles or Monte Carlo dropout) integrated directly into Expected Improvement. (iv) A hierarchical or windowed spectral-edge construction to keep eigendecomposition near-linear at industrial resolutions beyond 500^3^ voxels. Future work will focus on bridging the reality gap by incorporating domain adaptation techniques to align synthetic graphs with empirical tomograms. Additionally, we aim to extend the generative capability of the framework by replacing the random packing algorithm with score-based graph diffusion models, enabling the free-form generation of non-parametric topologies that extend beyond the constraints of convex ellipsoidal inclusions.

## Supporting information

S1 Appendix Pipeline pseudocode (Algorithms S1 through S3), structured comparison table (Table S1), and boundary condition schematic (Fig. S1). (PDF)

S2 Appendix Extended ablation results: LSE-MGN without spectral edges baseline. (PDF)

S3 AppendixSensitivity analysis: watershed marker spacing ($d_{m}$), Maximal Ball radius ratio (α), and synthetic segmentation noise (SNR).(PDF)

S4 Appendix k-eigenvector ablation study, eigendecomposition overhead, and scalability data (Table S4, Table S6). (PDF)

S5 AppendixSensitivity analysis: $\lambda_{cont}$ and $\lambda_{energy}$ (Tables S5a and S5b).Quantitative attention correlation analysis.(PDF)

S6 AppendixPreliminary validation on experimental synchrotron μCT scan.(PDF)

S7 AppendixAnalytical geometric analysis of the $45^{\circ }$ / $\rho_{c}\;\approx \;2.5$ optimum: projected shear area and expected crack-interception cross-section.(PDF)

S1 Data Machine-readable CSV files containing all numerical values underlying means, standard deviations, *R*^2^, NRMSE, and timing data reported in Tables 1 and 2, and all data used to construct Figures 3–6. (ZIP)
