# Supplementary material for: Graph-theoretic active learning for the closed-loop discovery of stochastic heterogeneous composites
Source: PLoS One. 2026 Aug 18;21(8):e0353692. doi: 10.1371/journal.pone.0353692 (PMC13485134; doi:10.1371/journal.pone.0353692)
Supplement: Supplementary file 1 [file pone.0353692.s001.pdf]

S1 Appendix. Pipeline Pseudocode, Comparison Table, and Boundary Condition Schematic

Algorithm S1: Graph Construction Pipeline

1. **Input:** 3-D segmented volume $I \in \{0, 1, 2\}^{L\times L\times L}$ (0 = pore, 1 = matrix, 2 = inclusion).
2. **Binarize solid phase:** $B \leftarrow (I > 0)$; compute distance transform $D \leftarrow \text{dist}(B)$.
3. **Seed markers:** $M \leftarrow \text{local\_maxima}(D,\ d_m = 5 \text{ voxels})$.
4. **Watershed segmentation:** $\mathcal{R} \leftarrow \text{watershed}(-D, M)$; merge adjacent regions whose mean boundary gradient $\|\nabla I\|_2 < \tau_{\text{merge}} = 0.15$.
5. **Prune artifacts:** remove components with $V_i < 8 \text{ voxels}^3$.
6. **Assign node types:** for each region $R_i \in \mathcal{R}$, assign $t_i \in \{\text{pore, matrix, inclusion}\}$ by majority vote of constituent voxels.
7. **Compute node features:** centroid $\mathbf{c}_i$, volume V_i, surface area A_i, sphericity Ψ_i, phase label t_i.
8. **Construct hydraulic edges $\mathcal{E}_{\text{hyd}}$:** for each pair of adjacent pore regions (i, j), extract throat cross-section area A_{ij}, perimeter P_{ij}; compute shape factor $\kappa_{ij} = A_{ij}/P_{ij}^2$ and conductance $g_{ij} = r_{ij}^4\kappa_{ij}/(\mu\, d_{ij})$.
9. **Construct mechanical edges $\mathcal{E}_{\text{mech}}$:** Delaunay triangulation on centroids; for each edge (i, j), compute ITZ metric $\omega_{ij} = \sum_k \|\nabla I(\mathbf{x}_k)\|_2 \cdot \Delta s$ along shortest solid-phase path γ_{ij}; z-score normalize: $\tilde{\omega}_{ij} = (\omega_{ij} - \mu_\omega)/\sigma_\omega$. Remove edges with $d_{ij} > 3\bar{d}$.
10. **Construct spectral edges $\mathcal{E}_{\text{long}}$:** compute normalized graph Laplacian $\mathbf{L}_{\text{sym}}$; extract $k = 16$ lowest eigenvectors $\{\mathbf{u}_1, \ldots, \mathbf{u}_{16}\}$ via ARPACK; connect each node to its k nearest neighbors in spectral embedding space under $\|\mathbf{u}_i - \mathbf{u}_j\|_2$.
11. **Output:** heterogeneous multigraph $\mathcal{G} = (\mathcal{V},\ \mathcal{E}_{\text{hyd}} \cup \mathcal{E}_{\text{mech}} \cup \mathcal{E}_{\text{long}})$.

Algorithm S2: LSE-MGN Forward Pass

1. **Input:** heterogeneous multigraph $\mathcal{G}$, node features $\{\mathbf{h}_i^{(0)}\}$.
2. **Encoder:** $\mathbf{h}_i^{(0)} \leftarrow \text{MLP}_{\text{enc}}(\mathbf{h}_i^{(0)})$, dimension $d = 128$.
3. **For $\ell = 1, \ldots, L_{\text{mp}} = 6$ message-passing layers:**
   (a) **Local stream:** aggregate messages over $\mathcal{E}_{\text{hyd}} \cup \mathcal{E}_{\text{mech}}$ using type-specific MLPs; $\mathbf{m}_i^{\text{local}} = \sum_{j\in\mathcal{N}_{\text{local}}(i)} \text{MLP}_{t(e_{ij})}([\mathbf{h}_i^{(\ell-1)} \| \mathbf{h}_j^{(\ell-1)} \| \mathbf{e}_{ij}])$.
   (b) **Long-range stream:** multi-head attention (4 heads) over $\mathcal{E}_{\text{long}}$; $\mathbf{m}_i^{\text{long}} = \text{MHA}(\mathbf{h}_i^{(\ell-1)},\ \{\mathbf{h}_j^{(\ell-1)}\}_{j\in\mathcal{N}_{\text{long}}(i)})$.
   (c) **Fusion:** $\mathbf{h}_i^{(\ell)} = \text{LayerNorm}(\mathbf{h}_i^{(\ell-1)} + \mathbf{m}_i^{\text{local}} + \mathbf{m}_i^{\text{long}})$.
4. **Decoder:** $\hat{y}_{\text{perm}} = \text{MLP}_{\text{perm}}(\text{mean-pool}(\{\mathbf{h}_i^{(L)}\}_{i\in\mathcal{V}_p}))$; $\hat{y}_{\text{surv}} = \text{MLP}_{\text{surv}}(\text{mean-pool}(\{\mathbf{h}_i^{(L)}\}))$.
5. **Output:** $\hat{y}_{\text{perm}},\ \hat{y}_{\text{surv}},\ \{\hat{\mathbf{u}}_i, \hat{\boldsymbol{\sigma}}_i, \hat{p}_i\}$.

Algorithm S3: GTAL Active Learning Loop

1. **Input:** design space $\Theta \subset \mathbb{R}^5$, oracle budget $N_{\max} = 80$, warm-start size $N_0 = 50$.
2. **Warm start:** sample $\{\boldsymbol{\theta}_i\}_{i=1}^{N_0}$ via Sobol sequence from Θ; evaluate oracle $\to \{y_i\}$; train LSE-MGN on $\mathcal{D}_0 = \{(\boldsymbol{\theta}_i, y_i)\}$.
3. **Fit GP:** train Anisotropic Matérn-5/2 GP on $\{(\boldsymbol{\theta}_i, \hat{y}_i^{\mathrm{MGN}})\}$ using BoTorch; optimize kernel hyperparameters via L-BFGS-B (20 restarts).
4. **For** $t = 1, \ldots, N_{\max} - N_0 = 30$ **iterations:**
   (a) Maximize Expected Improvement: $\boldsymbol{\theta}^* = \arg\max_{\boldsymbol{\theta}\in\Theta} \mathrm{EI}(\boldsymbol{\theta})$ via 10^4 Sobol candidates + 50-restart L-BFGS-B.
   (b) Generate microstructure from $\boldsymbol{\theta}^*$; run LBM + DEM oracle $\to y^*$.
   (c) Update dataset $\mathcal{D}_t \leftarrow \mathcal{D}_{t-1} \cup \{(\boldsymbol{\theta}^*, y^*)\}$; retrain LSE-MGN; refit GP.
5. **Output:** best design $\boldsymbol{\theta}^*_{\mathrm{best}} = \arg\max_{\boldsymbol{\theta}\in\mathcal{D}} y_{\mathrm{surv}}(\boldsymbol{\theta}) \cdot y_{\mathrm{perm}}(\boldsymbol{\theta})$.

Table S1. Structured Comparison of Graph-Based Surrogate Approaches

**Feature**	**Pore-GNN**	**MeshGraphNets**	**Standard GAT**	**GNN-Flow**	**LSE-MGN (Ours)**
Graph type	Homogeneous	Homogeneous mesh	Homogeneous	Homogeneous	Heterogeneous multigraph
Node types	1 (pore)	1 (mesh node)	1	1 (pore)	3 (pore, matrix, inclusion)
Edge types	1 (throat)	1 (mesh edge)	1	1 (throat)	3 (hydraulic, mechanical, spectral)
Long-range edges	×	×	×	×	✓ (Laplacian spectral)
Multiphysics	Permeability only	Single physics	Single task	Permeability only	Permeability + survival
Physics-informed loss	×	×	×	×	✓ ($\mathcal{L}_{\mathrm{cont}} + \mathcal{L}_{\mathrm{energy}}$)
Closed-loop BO	×	×	×	×	✓

Fig. S1. Boundary Condition Schematic

Fig 1. Boundary Condition Schematic for Physics-Informed Losses. Inlet nodes (left boundary, blue) have pressure fixed at $p_{\mathrm{in}} = 1.0$; outlet nodes (right boundary, red) are fixed at $p_{\mathrm{out}} = 0.0$. Interior pore nodes (gray) are subject to the continuity loss $\mathcal{L}_{\mathrm{cont}}$. Bottom-face nodes have displacement fixed at $\hat{\mathbf{u}}_i = \mathbf{0}$; top-face nodes receive prescribed compressive strain ε_{zz}. Interior solid nodes are subject to $\mathcal{L}_{\mathrm{energy}}$.
