# Supplementary material for: Graph-theoretic active learning for the closed-loop discovery of stochastic heterogeneous composites
Source: PLoS One. 2026 Aug 18;21(8):e0353692. doi: 10.1371/journal.pone.0353692 (PMC13485134; doi:10.1371/journal.pone.0353692)
Supplement: Supplementary file 2 [file pone.0353692.s002.pdf]

# S2 Appendix. Extended Ablation Results: LSE-MGN Without Spectral Edges

Table S2 presents the component ablation study isolating the contributions of spectral edges and physics-informed losses.

**Table S2.** Component ablation (5-seed mean ± std on test set).

**Cross-phase spectral edge gating experiment.** Restricting long-range connections to same-type node pairs (zeroing cross-phase spectral edges) yields $R^2_{\text{surv}} = 0.930 \pm 0.014$ vs. $0.941 \pm 0.013$ for the full model ($\Delta = -0.011$). This marginal degradation indicates that the attention mechanism already de-weights physically implausible cross-phase connections.

| **Model Variant** | **Spec. Edges** | **Phys. Loss** | **Permeability** | | **Survival Rate** | |
|---|---|---|---|---|---|---|
| | | | $R^2$ | NRMSE | $R^2$ | NRMSE |
| Standard GAT | × | × | $0.881 \pm 0.019$ | $0.093 \pm 0.011$ | $0.841 \pm 0.023$ | $0.118 \pm 0.015$ |
| LSE-MGN w/o Spec. Edges | × | ✓ | $0.912 \pm 0.018$ | $0.071 \pm 0.012$ | $0.887 \pm 0.021$ | $0.094 \pm 0.013$ |
| LSE-MGN w/o Phys. Loss | ✓ | × | $0.931 \pm 0.014$ | $0.052 \pm 0.009$ | $0.882 \pm 0.022$ | $0.096 \pm 0.014$ |
| **LSE-MGN (Full)** | ✓ | ✓ | $\mathbf{0.962 \pm 0.011}$ | $\mathbf{0.031 \pm 0.007}$ | $\mathbf{0.941 \pm 0.013}$ | $\mathbf{0.042 \pm 0.009}$ |
| Δ (Spectral edges, phys. loss fixed) | | | +0.050 | −0.040 | +0.054 | −0.052 |
| Δ (Phys. loss, spec. edges fixed) | | | +0.031 | −0.021 | +0.059 | −0.054 |

✓: present; ×: absent. Δ computed row-wise for fair comparison.
