# Supplementary material for: Graph-theoretic active learning for the closed-loop discovery of stochastic heterogeneous composites
Source: PLoS One. 2026 Aug 18;21(8):e0353692. doi: 10.1371/journal.pone.0353692 (PMC13485134; doi:10.1371/journal.pone.0353692)
Supplement: Supplementary file 3 [file pone.0353692.s003.pdf]

S3 Appendix. Sensitivity Analysis

We systematically perturbed three classes of graph-construction hyperparameters while holding all other settings at their defaults. All results are 5-seed mean ± std on the held-out test set (1,000 samples).

Table S3a. Effect of Watershed Marker Spacing d_m

($\alpha = 0.75$ fixed.)

d_m (vx)	$\lvert\mathcal{V}\rvert$	$\lvert\mathcal{E}\rvert$	R^2_{perm}	$\text{NRMSE}_{\text{perm}}$	R^2_{surv}	$\text{NRMSE}_{\text{surv}}$
3	1,241	13,651	0.943 ± 0.019	0.058 ± 0.013	0.929 ± 0.018	0.070 ± 0.012
**5**†	**798**	**8,778**	$\mathbf{0.962 \pm 0.011}$	$\mathbf{0.031 \pm 0.007}$	$\mathbf{0.941 \pm 0.013}$	$\mathbf{0.042 \pm 0.009}$
7	523	5,753	0.951 ± 0.017	0.043 ± 0.010	0.933 ± 0.017	0.056 ± 0.011
10	312	3,432	0.934 ± 0.022	0.057 ± 0.014	0.921 ± 0.021	0.068 ± 0.013

†Selected default. $\lvert\mathcal{V}\rvert/\lvert\mathcal{E}\rvert$: mean over 5,000 samples.

As d_m increases, the graph becomes coarser (fewer nodes) and prediction accuracy first improves then declines. The optimal $d_m = 5$ balances sufficient resolution against over-segmentation noise at $d_m = 3$.

Table S3b. Effect of Maximal Ball Radius Ratio α

($d_m = 5$ fixed.)

α	R^2_{perm}	$\text{NRMSE}_{\text{perm}}$	R^2_{surv}	$\text{NRMSE}_{\text{surv}}$
0.60	0.933 ± 0.021	0.059 ± 0.013	0.939 ± 0.015	0.045 ± 0.010
0.70	0.950 ± 0.015	0.044 ± 0.011	0.940 ± 0.014	0.044 ± 0.010
**0.75**†	$\mathbf{0.962 \pm 0.011}$	$\mathbf{0.031 \pm 0.007}$	$\mathbf{0.941 \pm 0.013}$	$\mathbf{0.042 \pm 0.009}$
0.80	0.944 ± 0.017	0.051 ± 0.012	0.940 ± 0.015	0.043 ± 0.010
0.90	0.931 ± 0.023	0.062 ± 0.015	0.938 ± 0.016	0.046 ± 0.011

†Selected default. R^2_{surv} range across all α: 0.003 (≤ 0.01).

Permeability prediction is sensitive to α because it controls throat geometry; survival rate is largely insensitive because mechanical edges do not depend on pore-throat topology.

Table S3c. Robustness to Synthetic Segmentation Noise

($d_m = 5$, $\alpha = 0.75$ fixed; Gaussian noise at varying SNR applied to CT volumes before watershed.)

SNR (dB)	R^2_{perm}	$\text{NRMSE}_{\text{perm}}$	R^2_{surv}	$\text{NRMSE}_{\text{surv}}$
40 (clean)	0.962 ± 0.012	0.031 ± 0.008	0.941 ± 0.014	0.042 ± 0.009
30	0.941 ± 0.021	0.053 ± 0.013	0.926 ± 0.019	0.063 ± 0.013
20	0.911 ± 0.031	0.079 ± 0.019	0.908 ± 0.025	0.081 ± 0.016

Graceful degradation: $\Delta R^2_{\text{perm}} = -0.051$ from 40 to 20 dB; $\Delta R^2_{\text{surv}} = -0.033$.

The model degrades gracefully under noise: even at 20 dB SNR (a level worse than typical synchrotron μCT), $R^2 > 0.90$ is maintained for both tasks.

Parameter-selection protocol. We recommend users start with defaults ($d_m = 5$, $\alpha = 0.75$) and only adjust if: (i) the mean graph size differs by $> 2\times$ from our reference ($\lvert\mathcal{V}\rvert \approx 800$), or (ii) the segmented volume shows visible over-/under-segmentation upon visual inspection. A diagnostic script is provided in the code repository.
