# Supplementary material for: Graph-theoretic active learning for the closed-loop discovery of stochastic heterogeneous composites
Source: PLoS One. 2026 Aug 18;21(8):e0353692. doi: 10.1371/journal.pone.0353692 (PMC13485134; doi:10.1371/journal.pone.0353692)
Supplement: Supplementary file 4 [file pone.0353692.s004.pdf]

S4 Appendix. k-Eigenvector Ablation, Eigendecomposition Overhead, and Scalability

Table S4. Ablation on Number of Spectral Eigenvectors k

($|\mathcal{V}| \approx 800$; 5-seed mean ± std on test set.)

k	$\bar{d}$	R^2_{perm}	$\text{NRMSE}_{\text{perm}}$	R^2_{surv}	$\text{NRMSE}_{\text{surv}}$	Inf. (ms)	Eigendecomp. (ms)
0 (no spectral)	6.2	0.912 ± 0.018	0.071 ± 0.012	0.887 ± 0.021	0.094 ± 0.013	9	0
4	10.2	0.932 ± 0.016	0.056 ± 0.010	0.904 ± 0.018	0.080 ± 0.012	10	85
8	14.2	0.948 ± 0.013	0.044 ± 0.009	0.921 ± 0.016	0.068 ± 0.011	11	120
**16**†	**22.2**	$\mathbf{0.962 \pm 0.011}$	$\mathbf{0.031 \pm 0.007}$	$\mathbf{0.941 \pm 0.013}$	$\mathbf{0.042 \pm 0.009}$	**12**	**180**
32	38.2	0.961 ± 0.012	0.032 ± 0.008	0.940 ± 0.014	0.043 ± 0.010	15	240
64	70.2	0.954 ± 0.014	0.038 ± 0.009	0.934 ± 0.016	0.049 ± 0.011	22	380

†Selected for all experiments. Performance plateaus at $k = 16$; $k = 64$ degrades due to over-connectivity.

Performance improves monotonically up to $k = 16$, then plateaus. At $k = 64$, the over-connected graph increases inference cost (+10 ms) with slight accuracy degradation, likely due to over-smoothing from excessive long-range message passing.

Table S6. Scalability with Domain Size L

(Single sample on NVIDIA A100 40 GB; CPU for preprocessing.)

L	$\lvert\mathcal{V}\rvert$	$\lvert\mathcal{E}\rvert$	**LSE-MGN**			**3D-ResNet-50**		
			Inf. (ms)	Preproc. (s)	VRAM (GB)	Inf. (ms)	Preproc. (ms)	VRAM (GB)
50	100	1,110	3	0.04	0.5	<2	3	1.8
**100**†	**800**	**8,880**	**12**	**0.19**	**1.8**	**8**	**5**	**14.2**
150	2,700	29,970	41	0.63	5.9	OOM	N/A	>40
200	6,300	69,930	89	1.82	13.7	OOM	N/A	OOM

†Training domain. OOM: out of GPU memory (>40 GB). LSE-MGN inference scales linearly with $|\mathcal{V}|$.

LSE-MGN maintains feasible VRAM (≤ 14 GB) up to $L = 200$, while 3D-ResNet-50 exceeds the 40 GB A100 memory at $L \geq 150$. The linear inference scaling of message passing ($O(|\mathcal{E}|)$ per layer) provides a decisive advantage for high-resolution microstructures.
