# Supplementary material for: Graph-theoretic active learning for the closed-loop discovery of stochastic heterogeneous composites
Source: PLoS One. 2026 Aug 18;21(8):e0353692. doi: 10.1371/journal.pone.0353692 (PMC13485134; doi:10.1371/journal.pone.0353692)
Supplement: Supplementary file 5 [file pone.0353692.s005.pdf]

S5 Appendix. Sensitivity Analysis: Physics Loss Weights and Attention Correlation

Table S5a. Sensitivity to λ_{cont}

($\lambda_{\text{energy}} = 0.05$ fixed; 5-seed mean ± std.)

λ_{cont}	R^2_{perm}	$\text{NRMSE}_{\text{perm}}$	R^2_{surv}	$\text{NRMSE}_{\text{surv}}$
0	0.944 ± 0.016	0.051 ± 0.011	0.940 ± 0.014	0.043 ± 0.010
0.01	0.953 ± 0.013	0.042 ± 0.009	0.941 ± 0.013	0.042 ± 0.009
**0.08**†	$\mathbf{0.962 \pm 0.011}$	$\mathbf{0.031 \pm 0.007}$	$\mathbf{0.941 \pm 0.013}$	$\mathbf{0.042 \pm 0.009}$
0.10	0.960 ± 0.012	0.034 ± 0.008	0.940 ± 0.014	0.043 ± 0.009
1.0	0.951 ± 0.015	0.045 ± 0.010	0.939 ± 0.015	0.047 ± 0.011

†Optimal. R^2_{perm} range: 0.018; R^2_{surv} insensitive (≤ 0.002).

The continuity loss primarily improves permeability prediction. Survival rate is insensitive to λ_{cont} because flux conservation is irrelevant to the mechanical task.

Table S5b. Sensitivity to λ_{energy}

($\lambda_{\text{cont}} = 0.08$ fixed; 5-seed mean ± std.)

λ_{energy}	R^2_{perm}	$\text{NRMSE}_{\text{perm}}$	R^2_{surv}	$\text{NRMSE}_{\text{surv}}$
0	0.930 ± 0.014	0.062 ± 0.010	0.881 ± 0.022	0.097 ± 0.015
0.01	0.941 ± 0.013	0.052 ± 0.009	0.907 ± 0.018	0.076 ± 0.013
**0.05**†	$\mathbf{0.962 \pm 0.011}$	$\mathbf{0.031 \pm 0.007}$	$\mathbf{0.941 \pm 0.013}$	$\mathbf{0.042 \pm 0.009}$
0.10	0.953 ± 0.014	0.042 ± 0.009	0.938 ± 0.015	0.045 ± 0.010
1.0	0.943 ± 0.018	0.053 ± 0.012	0.930 ± 0.018	0.057 ± 0.012

†Optimal. $\lambda_{\text{energy}} = 0$ yields $\Delta R^2_{\text{surv}} = -0.060$: decisive role for mechanical response.

The energy loss is critical for survival rate prediction. Removing it ($\lambda_{\text{energy}} = 0$) causes a 0.060 drop in R^2_{surv}, the largest single-component degradation observed in any ablation.

Quantitative Attention Correlation Analysis

We computed Spearman rank correlations between learned MHA attention weights $\bar{\alpha}_{ij}$ and two physical metrics across 200 test microstructures:

**Physical Metric**	**Spearman ρ_s**	**Interpretation**
Stress concentration $K_{t,ij}$	$+0.61 \pm 0.08$	Higher stress → higher attention
ITZ thickness δ_{ij}	-0.53 ± 0.10	Thicker ITZ → lower attention

Attention stability: across 200 test microstructures, the coefficient of variation (std/mean) of attention weights for the top-10 edges is 0.18 ± 0.05, indicating stable but geometry-dependent patterns. During linear elastic loading, long-range attention dominates (mean $\alpha_{\text{long}} = 0.62$); at fracture nucleation, local ITZ edges take over (mean $\alpha_{\text{short}} = 0.71$).
