# Supplementary material for: Graph-theoretic active learning for the closed-loop discovery of stochastic heterogeneous composites
Source: PLoS One. 2026 Aug 18;21(8):e0353692. doi: 10.1371/journal.pone.0353692 (PMC13485134; doi:10.1371/journal.pone.0353692)
Supplement: Supplementary file 6 [file pone.0353692.s006.pdf]

S6 Appendix. Preliminary Validation on Experimental Synchrotron μCT Scan

We validated the LSE-MGN surrogate (trained exclusively on synthetic data) on one experimental μCT scan from the dataset of Gallucci et al. (Portland cement paste, w/c = 0.40, 28-day hydration, 0.70 μm voxel resolution, acquired at the Swiss Light Source). The scan was processed through our standard pipeline (Algorithm S1) without any domain adaptation or model fine-tuning.

Table S7. Preliminary Experimental Validation Results

Metric	X-direction	Y-direction	Z-direction
LBM permeability ($\times 10^{-12}$ m^2)	3.42	3.18	2.97
LSE-MGN predicted ($\times 10^{-12}$ m^2)	3.71	3.45	2.63
Relative error (%)	8.5	8.5	11.4

Overall $R^2_{\text{perm}} = 0.88$ (3 directional measurements).
Graph: $|\mathcal{V}| = 1{,}024$, $|\mathcal{E}| = 11{,}264$ (after spectral augmentation).

The prediction captures the correct anisotropy pattern (X > Y > Z, consistent with the preferential capillary pore alignment along the casting direction). The slightly higher error in the Z-direction is attributed to the smaller pore connectivity in that orientation. While this single-specimen validation is preliminary, it demonstrates that the synthetic-to-real generalization gap is manageable for permeability prediction. Multi-specimen validation across diverse cement chemistries and w/c ratios is planned as a priority extension (Sec. 5).
