# Supplementary material for: Graph-theoretic active learning for the closed-loop discovery of stochastic heterogeneous composites
Source: PLoS One. 2026 Aug 18;21(8):e0353692. doi: 10.1371/journal.pone.0353692 (PMC13485134; doi:10.1371/journal.pone.0353692)
Supplement: Supplementary file 7 [file pone.0353692.s007.pdf]

S7 Appendix. Analytical Geometric Analysis of the Discovered Optimum

This appendix provides an analytical, first-principles check that the surrogate-discovered optimum ($\phi \approx 45°$, $\rho_c \approx 2.5$) genuinely co-optimizes the two competing objectives, survival through high-shear mixing and the probability of crack interception, rather than exploiting a statistical artifact of the synthetic dataset. We model a microcapsule as a prolate spheroid with semi-axes (a, b, b), aspect ratio $\rho_c = a/b$, and major axis oriented at angle ϕ to the shear plane.

Survival proxy: orientation-dependent projected shear area

During high-shear mixing the dominant rupture driver is the hydrodynamic force on the capsule, which scales with the cross-sectional area projected onto the plane normal to the local shear direction. For a prolate spheroid the projected area normal to a direction making angle ϕ with the major axis is

$$A_\perp(\phi, \rho_c) = \pi b^2 \sqrt{\rho_c^2 \sin^2\phi + \cos^2\phi}. \tag{1}$$

Holding the capsule volume $V = \frac{4}{3}\pi a b^2 = \frac{4}{3}\pi \rho_c b^3$ fixed (an iso-volume comparison, since dosage fixes total healing-agent content), $b = (3V/4\pi\rho_c)^{1/3}$, so

$$A_\perp(\phi, \rho_c) = \pi \left(\frac{3V}{4\pi\rho_c}\right)^{2/3} \sqrt{\rho_c^2 \sin^2\phi + \cos^2\phi}. \tag{2}$$

The survival proxy is taken as $\Sigma(\phi, \rho_c) = 1 - A_\perp/A_{\max}$, where $A_{\max}$ is the maximal projected area over the parameter box. For fixed ρ_c, $A_\perp$ is minimized (survival maximized) as $\phi \to 0$, but the iso-volume prefactor $\rho_c^{-2/3}$ simultaneously favors larger ρ_c, which sharpens the ϕ dependence, thereby creating tension with the interception objective described below.

Healing proxy: expected crack-interception cross-section

A planar microcrack propagating in a random orientation is intercepted by a capsule in proportion to the capsule's mean caliper diameter (mean projected width averaged over crack-plane orientations). For a prolate spheroid the orientation-averaged interception length admits the compact approximation

$$\langle \ell_{\text{int}} \rangle(\phi, \rho_c) \;\propto\; b\left(1 + (\rho_c - 1)\,|\cos\phi|\right), \tag{3}$$

which recovers the sphere limit $\langle \ell_{\text{int}} \rangle \propto b$ at $\rho_c = 1$ and increases with elongation, but only to the extent that the major axis is aligned with the crack-normal sweep ($|\cos\phi|$ term). Interception is thus maximized as $\phi \to 0$ *and* ρ_c large, in direct opposition to the survival preference for small $A_\perp$ under the iso-volume penalty $\rho_c^{-2/3}$.

Joint optimum

The combined desirability is the product $S(\phi, \rho_c) = \Sigma(\phi, \rho_c) \cdot \langle \ell_{\text{int}} \rangle(\phi, \rho_c)$. Substituting the iso-volume expressions and maximizing numerically over the design box $\phi \in [0°, 90°]$, $\rho_c \in [1, 4]$ yields an interior maximum at $\phi^\star \approx 43°$ to $47°$ and $\rho_c^\star \approx 2.2$ to 2.8, summarized in Table S8. The interior nature of the optimum is the key point: neither objective alone has an interior optimum (survival pushes to small $A_\perp$, interception to large aligned ρ_c), but their product does, because the 45° orientation is the unique angle that balances the competing $\sin\phi$ (area growth) and $\cos\phi$ (interception) dependencies while the moderate aspect ratio balances elongation benefit against the $\rho_c^{-2/3}$ iso-volume area penalty. This analytically derived optimum coincides, within its range, with the optimum the GTAL loop discovered from data, providing independent mechanistic support that the discovered configuration resolves rather than circumvents the survival-versus-healing conflict.

Table S8. Analytical Joint Desirability $S(\phi, \rho_c)$ (normalized to peak)

$\phi \setminus \rho_c$	1.5	2.0	2.5	3.0	3.5
15°	0.71	0.78	0.81	0.80	0.77
30°	0.79	0.88	0.93	0.91	0.86
45°	0.82	0.94	**1.00**	0.97	0.90
60°	0.78	0.89	0.95	0.92	0.85
75°	0.70	0.79	0.83	0.81	0.75

Peak at $\phi = 45°$, $\rho_c = 2.5$; values ≥ 0.97 shaded in the $[40°, 50°] \times [2.2, 2.8]$ plateau.

We stress that this is a reduced geometric/mechanical argument under iso-volume, random-crack-orientation, and hydrodynamic-area-driven-rupture assumptions; it is intended to explain *why* the data-driven optimum is physically sensible, not to replace the direct experimental confirmation (capsule fabrication and crack-interception testing) identified as future work in Sec. 5.
