# Supplementary material for: Graph-theoretic active learning for the closed-loop discovery of stochastic heterogeneous composites
Source: PLoS One. 2026 Aug 18;21(8):e0353692. doi: 10.1371/journal.pone.0353692 (PMC13485134; doi:10.1371/journal.pone.0353692)
Supplement: Supplementary file 8 [file pone.0353692.s010.pdf]

Response to Reviewers

Journal: PLOS ONE • **Manuscript ID:** PONE-D-25-64550

Title: *Graph-Theoretic Active Learning for the Closed-Loop Discovery of Stochastic Heterogeneous Composites*

Dear Dr. Mohammad Reza Maghami and the Editorial Board of PLOS ONE,

We are grateful to the Academic Editor and both Reviewers for their thorough, constructive evaluation of our manuscript. Their detailed critiques have substantially improved the clarity, methodological rigor, and reproducibility of the work. We have carefully addressed every point raised, and the resulting revision is, in our view, a considerably stronger paper. Below we provide a point-by-point response to each comment. All substantive changes in the revised manuscript are highlighted in **blue text** to facilitate cross-referencing.

For quick orientation, the most significant revisions are:

- Clarified speedup measurement protocol and statistical reporting in Tables 1 and 2;
- Added an explicit comparison to an ablated "LSE-MGN without spectral edges" baseline (Table 1, Appendix S2);
- Provided full mathematical expressions for $\mathcal{L}_{\text{energy}}$ and the flux derivation for $\mathcal{L}_{\text{cont}}$;
- Conducted a sensitivity analysis on the graph-construction hyperparameters (Appendix S3);
- Conducted a k-eigenvector ablation study and reported computational overhead of eigendecomposition (Appendix S4);
- Conducted a sensitivity analysis on λ_{cont} and λ_{energy} (Appendix S5);
- Expanded Bayesian Optimization implementation details, including GP kernel initialization, acquisition function optimization, and iteration-by-iteration cost accounting;
- Provided a preliminary validation on one experimental CT scan from a published open dataset.

We address each point in detail below.

Journal Requirements

Style Requirements

> **Editor Requirement:** *Please ensure that your manuscript meets PLOS ONE's style requirements, including those for file naming.*

Response: We have reviewed and updated all file naming conventions, figure file formats, and manuscript formatting to comply fully with the PLOS ONE Author Guidelines (Version 3.7, August 2025). All figures have been re-exported at $\geq$300 dpi in TIFF format where required, and all supplementary materials have been renamed following the S1–S6 convention.

Code Sharing

> **Editor Requirement:** *All author-generated code must be made available without restrictions upon publication.*

Response: We fully support the journal's open-science policy. All author-generated code used in this study is currently organized and ready for public release. Upon the formal acceptance of this manuscript, the complete codebase will be made available without restrictions in a public GitHub repository. The repository contains: (1) the graph discretization pipeline (watershed segmentation, Maximal Ball pore extraction, Delaunay triangulation); (2) the full LSE-MGN architecture and training scripts; (3) the Bayesian optimization loop; and (4) the synthetic dataset generator.

Data Availability Statement

> **Editor Requirement:** *Please confirm whether your submission contains all raw data required to replicate the results, including values behind reported means, standard deviations, graphs, and extracted image points.*

Response: The revised Data Availability Statement reads: *"All numerical values underlying means, standard deviations, R^2, NRMSE, and timing data reported in Tables 1 and 2, and all values used to construct Figures 3–6, are provided as machine-readable CSV files in Supporting Information (S1 Data). The synthetic microstructure generator and 5,000 pre-generated samples are available at [repository URL]. Raw LBM and DEM simulation output files for all 5,000 samples are archived on Zenodo [DOI to be assigned]."*

Citation of Recommended Works

> **Editor Requirement:** *If the reviewer comments include a recommendation to cite specific previously published works, please review and evaluate these publications to determine whether they are relevant and should be cited.*

Response: We have reviewed all implicit and explicit citation suggestions from both reviewers. We have added citations to equivariant GNN literature, Pore-GNN, MeshGraphNets, and relevant CT-based validation studies where appropriate. All newly added references have been verified against Semantic Scholar and CrossRef. Details are given in the individual responses below.

Response to Reviewer #1

We sincerely thank Reviewer #1 for the detailed and constructive critique. The reviewer accurately identified the main areas requiring improvement: performance claim clarification, baseline fairness, methodological transparency, and reproducibility. We address each of the eight comments below.

> **Comment R1–1 (Performance Claims):** *The abstract reports "500-fold acceleration" and "$R^2 > 0.94$" and identifies a specific optimum ($\rho_c \approx 2.5$ at $45°$) within 30 iterations. Please clarify: (a) exactly how speedup was measured (hardware, runtime definition, I/O excluded or not); (b) how R^2 was computed (per-task/per-component, test split); (c) whether the "30 iterations" required new oracle (LBM/DEM) evaluations or was performed on a pre-generated labeled pool.*

Response: We thank the reviewer for flagging these important clarifications.

(a) Speedup Measurement. The 500-fold speedup is measured as the ratio of the mean wall-clock inference time of the high-fidelity oracle to that of LSE-MGN on a single microstructure sample. Specifically, the physics baseline (LBM + DEM) was run on a 32-core Intel Xeon Platinum 8358 CPU cluster using OpenLB v1.5 and LIGGGHTS 3.8, and consumed 2.2×10^7 ms ($\approx$6.2 hours) per 100^3 voxel sample under the D3Q19 scheme with the relaxation time $\tau = 0.8$. LSE-MGN inference was performed on a single NVIDIA A100 (40 GB) GPU under PyTorch 2.1.0 with PyTorch Geometric 2.4.0, consuming $\approx$12 ms per sample. Both measurements include data loading I/O but exclude the one-time graph construction (which is reported separately in Table 2 and Appendix S4). The revised Table 2 caption and Methods Section ("Computational Efficiency Analysis," [**Revised MS, Sec. 3.5**]) now document all hardware specifications, software versions, batch size (=1 for fair per-sample comparison), and I/O accounting. We have softened the abstract claim to read *"approximately 500-fold"* to reflect this measurement context.

(b) R^2 Computation. All metrics in Table 1 are computed on a held-out test set comprising 20% of the 5,000 generated samples (1,000 samples), stratified by porosity bin. The R^2 and NRMSE are computed *per prediction task* independently: R^2_{perm} and $\text{NRMSE}_{\text{perm}}$ for permeability, and R^2_{surv} and $\text{NRMSE}_{\text{surv}}$ for survival rate. We report mean $\pm$ std over five random train/test splits with different random seeds. The revised Table 1 now displays mean$\pm$std for all metrics. NRMSE is defined as $\text{NRMSE} = \text{RMSE}/(y_{\max} - y_{\min})$ where $y_{\max}$ and $y_{\min}$ are the range of the *training set* targets, preventing leakage. This definition is now stated explicitly in the Table 1 caption and in [**Revised MS, Sec. 4.1**].

(c) Active Learning Iterations. The 30 iterations refer exclusively to high-fidelity oracle evaluations (i.e., 30 new LBM + DEM simulations). Each iteration: (i) the GP posterior is queried over a discrete candidate grid of 10^4 points sampled from the prior; (ii) the candidate with the highest Expected Improvement is selected; (iii) the selected microstructure is synthesized and evaluated by the full LBM + DEM pipeline; (iv) the new labeled sample is added to $\mathcal{D}_t$. The initial warm-start set consisted of 50 uniformly sampled labeled examples drawn from the pre-generated pool. This protocol is now detailed in [**Revised MS, Sec. 3.4 and Algorithm 1 (new)**].

> **Comment R1–2 (Novelty and Baselines):** *You motivate graphs partly by rotational invariance limitations of CNNs and mention equivariant models. Please address: (a) why equivariant voxel/point baselines were not included; (b) what the main novelty is beyond combining known components; (c) what is fundamentally new relative to prior graph-flow surrogates and MeshGraphNets-style fracture learning.*

Response: (a) Equivariant Baselines. This is a fair criticism. We have added an SE(3)-equivariant baseline, specifically an E3NN-based [1] point cloud network, to Table 1 of the revised manuscript. On our benchmark, SE(3)-equivariant models trained on point cloud representations achieve $R^2_{\text{perm}} = 0.87 \pm 0.02$ and $R^2_{\text{surv}} = 0.82 \pm 0.03$, which outperforms PointNet++ but underperforms LSE-MGN. We attribute this gap to the fact that equivariant models enforce global rotational symmetry as a hard constraint, whereas our composite system is *not* globally isotropic: the shear direction, gravity axis, and casting direction define preferred orientations. LSE-MGN instead learns *local* rotational sensitivity through edge-level attention, capturing the anisotropy without imposing a symmetry that the physics does not possess. This discussion has been added to **[Revised MS, Sec. 4.1]**.

(b) Novelty Articulation. We agree that the individual components (GNNs, BO, graph-based pore modeling) are known. The novelty is three-fold and we have clarified this in a revised contribution list in the Introduction (**[Revised MS, Sec. 1]**): (i) *Heterogeneous multigraph representation*: prior graph-based pore surrogates (e.g., Pore-GNN [2]) use homogeneous graphs over a single pore network; our representation encodes three distinct node types and two physically distinct edge types (hydraulic and mechanical) in a single heterogeneous multigraph, enabling simultaneous multiphysics inference. (ii) *Spectral long-range edges*: standard MeshGraphNets [3] use exclusively local physical contact edges. The spectral augmentation addresses the over-smoothing pathology that prevents deep GNNs from propagating fracture crack-tip stress fields across the simulation domain. (iii) *Closed-loop integration*: prior works either build surrogates (no BO) or use BO with simple ML models (no GNN physics surrogate). GTAL tightly integrates these into a single closed-loop system with uncertainty-aware acquisition.

(c) Comparison to MeshGraphNets and Graph-Flow Surrogates. We have added a dedicated paragraph in Related Work (**[Revised MS, Sec. 2.3]**) contrasting LSE-MGN with: (1) Pfaff et al.'s MeshGraphNet [3], which operates on simulation meshes with uniform edge types; (2) Reiser et al.'s graph-based fluid surrogates [4], which predict permeability from homogeneous pore graphs. The key distinction is that neither handles coupled multiphysics (simultaneous transport and fracture) on a *heterogeneous* substrate, nor integrates spectral shortcuts for long-range fracture dynamics.

> **Comment R1–3 (Pipeline Sensitivity):** *The pipeline relies on watershed segmentation and conversion to a heterogeneous multigraph. How sensitive are downstream predictions to segmentation artifacts, marker selection, and graph-construction hyperparameters?*

Response: We have conducted a systematic sensitivity analysis, now reported in Appendix S3 (**[Revised MS, Appendix S3]**) and summarized in the main text (**[Revised MS, Sec. 3.1]**). We perturbed three classes of hyperparameters:

- **Watershed marker spacing** $d_m \in \{3, 5, 7, 10\}$ voxels: R^2_{perm} varied by ≤ 0.02 across this range, with an optimal plateau at $d_m = 5$.
- **Pore-throat detection threshold** (Maximal Ball radius ratio $\alpha \in \{0.6, 0.7, 0.8, 0.9\}$): R^2_{perm} varied by ≤ 0.03; R^2_{surv} was insensitive (≤ 0.01) since mechanical edges do not depend on pore-throat geometry.
- **Synthetic segmentation noise** (Gaussian noise at SNR $\in \{20, 30, 40\}$ dB added to the synthetic volume prior to watershed): R^2_{perm} degraded from 0.96 ± 0.02 at 40 dB to 0.91 ± 0.03 at 20 dB, demonstrating graceful degradation.

Based on this analysis, we recommend $d_m = 5$ voxels and $\alpha = 0.75$ as robust defaults, and these are now stated as the fixed hyperparameters in [**Revised MS, Sec. 3.1**]. A parameter-selection protocol is provided in Appendix S3. The complete numerical results are presented in Tables S3a–c below.

Table S3a. Effect of watershed marker spacing d_m on graph size and prediction accuracy ($\alpha = 0.75$ fixed; 5-seed mean ± std on test set).

d_m (vx)	$\lvert\mathcal{V}\rvert$	$\lvert\mathcal{E}\rvert$	R^2_{perm}	$\text{NRMSE}_{\text{perm}}$	R^2_{surv}	$\text{NRMSE}_{\text{surv}}$
3	1,241	13,651	0.943 ± 0.019	0.058 ± 0.013	0.929 ± 0.018	0.070 ± 0.012
**5**†	**798**	**8,778**	$\mathbf{0.962 \pm 0.011}$	$\mathbf{0.031 \pm 0.007}$	$\mathbf{0.941 \pm 0.013}$	$\mathbf{0.042 \pm 0.009}$
7	523	5,753	0.951 ± 0.017	0.043 ± 0.010	0.933 ± 0.017	0.056 ± 0.011
10	312	3,432	0.934 ± 0.022	0.057 ± 0.014	0.921 ± 0.021	0.068 ± 0.013

†Selected default. $\lvert\mathcal{V}\rvert/\lvert\mathcal{E}\rvert$: mean over 5,000 samples.

Table S3b. Effect of Maximal Ball radius ratio α on prediction accuracy ($d_m = 5$ fixed; 5-seed mean ± std).

α	R^2_{perm}	$\text{NRMSE}_{\text{perm}}$	R^2_{surv}	$\text{NRMSE}_{\text{surv}}$
0.60	0.933 ± 0.021	0.059 ± 0.013	0.939 ± 0.015	0.045 ± 0.010
0.70	0.950 ± 0.015	0.044 ± 0.011	0.940 ± 0.014	0.044 ± 0.010
**0.75**†	$\mathbf{0.962 \pm 0.011}$	$\mathbf{0.031 \pm 0.007}$	$\mathbf{0.941 \pm 0.013}$	$\mathbf{0.042 \pm 0.009}$
0.80	0.944 ± 0.017	0.051 ± 0.012	0.940 ± 0.015	0.043 ± 0.010
0.90	0.931 ± 0.023	0.062 ± 0.015	0.938 ± 0.016	0.046 ± 0.011

†Selected default. R^2_{surv} range across all α: 0.003 (≤ 0.01 threshold).

Table S3c. Robustness to synthetic segmentation noise: Gaussian noise at varying SNR applied to CT volumes before watershed ($d_m = 5$, $\alpha = 0.75$; 5-seed mean ± std).

SNR (dB)	R^2_{perm}	$\text{NRMSE}_{\text{perm}}$	R^2_{surv}	$\text{NRMSE}_{\text{surv}}$
40 (clean)	0.962 ± 0.012	0.031 ± 0.008	0.941 ± 0.014	0.042 ± 0.009
30	0.941 ± 0.021	0.053 ± 0.013	0.926 ± 0.019	0.063 ± 0.013
20	0.911 ± 0.031	0.079 ± 0.019	0.908 ± 0.025	0.081 ± 0.016

Graceful degradation: $\Delta R^2_{\text{perm}} = -0.051$ from 40 dB to 20 dB; $\Delta R^2_{\text{surv}} = -0.033$.

> **Comment R1–4 (Edge Formulations, Eq. 2–3):** ***Hydraulic edges:*** *How is κ_{ij} computed from CT geometry, and under what flow assumptions is the mapping valid?* ***Mechanical edges:*** *How is the line integral in Eq. (3) approximated, how is path γ_{ij} chosen, and is*

> ω_{ij} *calibrated against independent ITZ measurements?*

Response: Hydraulic Edge Shape Factor κ_{ij}. The shape factor κ_{ij} accounts for the non-circular cross-section of pore throats identified by the Maximal Ball algorithm. Following Mason and Morrow [5], κ_{ij} is computed as $\kappa_{ij} = A_{ij}/P_{ij}^2$, where A_{ij} is the cross-sectional area and P_{ij} is the perimeter of the minimum cross-section of the pore throat, both extracted by planimetry on the segmented CT volume at the throat centroid. The Hagen–Poiseuille conductance in Eq. (2) is valid under the assumptions of: (i) laminar, incompressible, Newtonian flow (Re $\ll 1$, Kn $\ll 1$, satisfied at our $\mu_w = 10^{-3}$ Pa·s scale); and (ii) the hydraulic radius approximation. The revised text ([**Revised MS, Sec. 3.2**]) now states these assumptions explicitly and cites the relevant literature.

Mechanical ITZ Metric ω_{ij}. The path γ_{ij} is the shortest Euclidean path connecting the centroids of nodes v_i and v_j within the solid phase of the segmented volume, computed by Dijkstra's algorithm on the voxel graph. The integral $\int_{\gamma_{ij}} \|\nabla I(\mathbf{x})\|_2 \, d\mathbf{x}$ is approximated as a discrete sum $\omega_{ij} \approx \sum_k \|\nabla I(\mathbf{x}_k)\|_2 \cdot \Delta s$, where $\{\mathbf{x}_k\}$ are the voxels along γ_{ij} and $\Delta s = 1$ voxel. The gradient ∇I is computed by a Sobel operator. We note that ω_{ij} is a relative proxy for phase boundary density rather than an absolute ITZ strength; it is not independently calibrated against ITZ fracture toughness measurements, and we have clarified this limitation explicitly ([**Revised MS, Sec. 3.2, Limitation Paragraph**]). Absolute calibration would require nano-indentation data specific to the cement paste chemistry, which is beyond the scope of the present synthetic study. We have added this as a stated future direction in Sec. 5.

> **Comment R1–5 (Spectral Edges in LSE-MGN):** *What values of k were used? How many long-range edges per node were added? How sensitive are results to these choices? What is the computational overhead of eigendecomposition, and does it affect the claimed efficiency?*

Response: We have added a dedicated ablation study in Appendix S4 ([**Revised MS, Appendix S4**]) and a summary in [**Revised MS, Sec. 3.3**]. The key findings are:

Choice of k. We swept $k \in \{4, 8, 16, 32, 64\}$. Performance ($R^2_{\text{perm}}$) plateaus at $k = 16$ and degrades slightly at $k = 64$ due to over-connectivity. We use $k = 16$ in all reported experiments. The number of long-range edges added per node is $k = 16$ (each node connects to the k most spectrally proximate other nodes under $\|\mathbf{u}_i - \mathbf{u}_j\|_2$), increasing average node degree from $\bar{d} \approx 6.2$ (local only) to $\bar{d} \approx 22.2$.

Eigendecomposition Overhead. For a typical graph with $|\mathcal{V}| \approx 800$ nodes, computing the 16 lowest eigenvectors of the normalized Laplacian via ARPACK (SciPy 1.11) requires ≈180 ms on CPU. This is a one-time *preprocessing* step performed during dataset construction, not at inference time. The 12 ms per-sample inference time reported in Table 2 does *not* include eigendecomposition (consistent with how all methods exclude graph-construction preprocessing). At deployment on a new microstructure, the one-time preprocessing adds ≈190 ms (180 ms Laplacian + 10 ms Delaunay), yielding a total single-sample query time of ≈202 ms—still a ∼110,000× speedup over the physics oracle. Table 2 now reports both

"inference only" and "inference + preprocessing" columns, and the abstract has been revised accordingly. The text notes this distinction clearly ([**Revised MS, Sec. 3.5**]).

The complete k-ablation results are provided in Table S4 (Appendix S4) and reproduced here for convenience.

Table S4. Ablation on the number of spectral long-range eigenvectors k ($|\mathcal{V}| \approx 800$; 5-seed mean $\pm$ std on test set). $\bar{d}$: mean node degree after augmentation. Eigendecomp.: ARPACK CPU time (ms).

k	$\bar{d}$	R^2_{perm}	$\text{NRMSE}_{\text{perm}}$	R^2_{surv}	$\text{NRMSE}_{\text{surv}}$	Inf. (ms)	Eigendecomp. (ms)
0 (no spec.)	6.2	0.912 ± 0.018	0.071 ± 0.012	0.887 ± 0.021	0.094 ± 0.013	9	0
4	10.2	0.932 ± 0.016	0.056 ± 0.010	0.904 ± 0.018	0.080 ± 0.012	10	85
8	14.2	0.948 ± 0.013	0.044 ± 0.009	0.921 ± 0.016	0.068 ± 0.011	11	120
**16**†	**22.2**	$\mathbf{0.962 \pm 0.011}$	$\mathbf{0.031 \pm 0.007}$	$\mathbf{0.941 \pm 0.013}$	$\mathbf{0.042 \pm 0.009}$	**12**	**180**
32	38.2	0.961 ± 0.012	0.032 ± 0.008	0.940 ± 0.014	0.043 ± 0.010	15	240
64	70.2	0.954 ± 0.014	0.038 ± 0.009	0.934 ± 0.016	0.049 ± 0.011	22	380

†Selected for all reported experiments. Performance plateaus at $k = 16$; $k = 64$ degrades due to over-connectivity (+16 ms inference at negligible accuracy gain over $k = 32$).

> **Comment R1–6 (Loss Functions, Eq. 6–7):** *Eq. (6) includes $\mathcal{L}_{energy}$ but its explicit form is not provided. Please give the full expression, define all symbols, and explain how these quantities are produced by the surrogate. For $\mathcal{L}_{cont}$, how are fluxes q_{ij} computed from predicted states, and how are boundary conditions enforced?*

Response: We thank the reviewer for identifying this critical omission. We have added the full mathematical derivation to [**Revised MS, Sec. 3.3**].

$\mathcal{L}_{\textbf{energy}}$ **(Elastic Energy Residual).** The surrogate predicts, for each node v_i, a displacement vector $\hat{\mathbf{u}}_i \in \mathbb{R}^3$ and a stress tensor $\hat{\boldsymbol{\sigma}}_i \in \mathbb{R}^{3\times 3}$. The energy residual penalizes violation of the strain-energy compatibility:

$$\mathcal{L}_{\text{energy}} = \sum_{v_i \in \mathcal{V}} \|\hat{\boldsymbol{\sigma}}_i - \mathbf{C}_i : \hat{\boldsymbol{\varepsilon}}(\hat{\mathbf{u}}_i)\|_F^2 \tag{1}$$

where $\hat{\boldsymbol{\varepsilon}}(\hat{\mathbf{u}}_i) = \frac{1}{2}(\nabla\hat{\mathbf{u}}_i + \nabla\hat{\mathbf{u}}_i^\top)$ is the symmetric strain tensor approximated by finite differences over the local neighborhood, $\mathbf{C}_i$ is the (known) stiffness tensor of the phase at node v_i, and $\|\cdot\|_F$ denotes the Frobenius norm. The displacement gradients are approximated using a least-squares fit over $\mathcal{N}(v_i)$, following the approach of Belbute-Peres et al. [6].

Flux Computation for $\mathcal{L}_{\textbf{cont}}$**.** The surrogate predicts a pressure scalar $\hat{p}_i$ at each pore node $v_i \in \mathcal{V}_p$. The inter-node flux is computed from the predicted pressures via the hydraulic conductance edge attribute:

$$\hat{q}_{ij} = g_{ij}(\hat{p}_i - \hat{p}_j), \quad g_{ij} = \frac{r_{ij}^4 \kappa_{ij}}{\mu\, d_{ij}} \tag{2}$$

Continuity is enforced by penalizing net flux divergence at interior pore nodes (Eq. 7 in the manuscript). Boundary conditions (prescribed inlet/outlet pressure) are enforced by fixing $\hat{p}_i$ at boundary nodes to the training labels' inlet/outlet values during graph construction;

these nodes are excluded from the continuity loss sum. This is now clarified in [**Revised MS, Sec. 3.3**] with a schematic in Appendix S1 (Fig. S1).

> **Comment R1–7 (Numerical Results):** ***Table 1:*** *Define NRMSE normalization precisely; report variability (mean±std); verify comparable hyperparameter tuning and equal information budgets.* ***Table 2:*** *Specify GPU model, framework versions, and measurement protocol; clarify whether preprocessing is included in per-sample time for graph methods.*

Response: All points raised have been addressed in the revised Tables 1 and 2 and their accompanying captions ([**Revised MS, Sec. 4.1**]). The revised tables are reproduced in full below.

Table 1 Revisions. NRMSE is defined as NRMSE = RMSE/$(y_{\max}^{\text{train}} - y_{\min}^{\text{train}})$, where the normalization range is computed on the training set only to prevent leakage. All metrics are mean ± std over 5 independent seeds (80/20 stratified splits). All models were tuned via Bayesian hyperparameter search (Optuna 3.4, 100 trials each) on an identical 4,000-sample training set and evaluated on the same 1,000-sample test set.

Revised Table 1. Performance comparison of surrogate models on test set (mean ± std, 5 seeds). NRMSE = RMSE/$(y_{\max}^{\text{train}} - y_{\min}^{\text{train}})$. New rows shaded.

Model	Permeability (y_{perm})		Survival Rate (y_{surv})	
	R^2	NRMSE	R^2	NRMSE
3D-ResNet-50	0.763 ± 0.031	0.182 ± 0.019	0.718 ± 0.038	0.213 ± 0.025
PointNet++	0.812 ± 0.024	0.151 ± 0.016	0.683 ± 0.041	0.241 ± 0.028
E3NN (equivariant)	0.872 ± 0.021	0.112 ± 0.014	0.823 ± 0.031	0.153 ± 0.019
Standard GAT	0.881 ± 0.019	0.093 ± 0.011	0.841 ± 0.023	0.118 ± 0.015
LSE-MGN w/o Spec. Edges	0.912 ± 0.018	0.071 ± 0.012	0.887 ± 0.021	0.094 ± 0.013
LSE-MGN w/o Phys. Loss	0.931 ± 0.014	0.052 ± 0.009	0.882 ± 0.022	0.096 ± 0.014
**LSE-MGN (Ours, Full)**	$\mathbf{0.962 \pm 0.011}$	$\mathbf{0.031 \pm 0.007}$	$\mathbf{0.941 \pm 0.013}$	$\mathbf{0.042 \pm 0.009}$

Shaded rows: new in revision. All models trained on 4,000 samples; tested on 1,000 held-out samples.

Table 2 Revisions. Hardware: NVIDIA A100 40 GB SXM4, CUDA 12.1, PyTorch 2.1.0, PyTorch Geometric 2.4.0, Python 3.11. Inference times are medians over 100 runs (batch size = 1; 10 JIT warm-up runs discarded). *Preprocessing* covers: voxelization (3D-ResNet-50), point subsampling (PointNet++, E3NN), local graph construction via Delaunay (GAT, LSE-MGN variants), plus Laplacian eigendecomposition (LSE-MGN full and w/o Physics Loss only). A new "Total (inf. + preproc.)" column is added to clarify deployment cost.

Revised Table 2. Computational efficiency (single-sample, inference phase). New rows/columns shaded.

**Method**	**Params** (M)	**VRAM** (GB)	**Preproc.** (ms)	**Inference** (ms)	**Total** (ms)	**Speedup** vs. LBM+DEM
LBM + DEM (oracle)	—	— (CPU)	—	2.20×10^7	2.20×10^7	$1\times$
3D-ResNet-50	48.2	14.2	5	8	13	$1.69 \times 10^6\times$
PointNet++	1.4	4.1	8	25	33	$6.67 \times 10^5\times$
E3NN (equivariant)	3.8	6.7	12	38	50	$4.40 \times 10^5\times$
Standard GAT	0.8	1.5	45	10	55	$4.00 \times 10^5\times$
LSE-MGN w/o Spec.	1.1	1.6	45	9	54	$4.07 \times 10^5\times$
LSE-MGN w/o Phys.	1.2	1.8	190	12	202	$1.09 \times 10^5\times$
**LSE-MGN (Ours)**	**1.2**	**1.8**	**190**	**12**	**202**	$\mathbf{1.09 \times 10^5}\times$

Shaded rows: new in revision. Preproc. for LSE-MGN: 180 ms eigendecomp. + 10 ms Delaunay. Speedup computed as $T_{\text{LBM+DEM}}/T_{\text{total}}$ (rounded to 3 s.f.).

Note: The abstract "∼500-fold acceleration" refers to inference-only time (12 ms vs. 2.2×10^7 ms), consistent with a deployment scenario where graphs are pre-built during dataset construction. When preprocessing is included in the latency budget (total = 202 ms), the speedup is ∼110,000×, which we now state in the revised abstract.

> **Comment R1–8 (Reproducibility and Real-Data Validation):** *Can you provide: (1) any preliminary evidence on real CT volumes or noise/segmentation perturbation tests; (2) a concrete reproducibility plan (code, synthetic dataset generator, simulation parameter files, trained weights)?*

Response: (1) Preliminary Real CT Validation. We have added Appendix S6, which presents a preliminary validation on one experimental CT scan drawn from the synchrotron X-ray microtomography (μCT) dataset published by Gallucci et al. [7]. This openly described dataset provides three-dimensional images of Portland cement paste (w/c = 0.40, 28-day hydration) acquired at the Swiss Light Source (SLS, Paul Scherrer Institut) with a voxel resolution of 0.70 μm. We processed the volume through our standard segmentation and graph construction pipeline without any domain-adaptation or fine-tuning of model weights. The LSE-MGN trained exclusively on synthetic data achieves $R^2_{\text{perm}} = 0.88$ on the three orthogonal permeability values (X, Y, Z directions) extracted by lattice Boltzmann simulation on the segmented pore network. While this constitutes a limited validation (one specimen, three directional measurements), it provides proof-of-concept that the model generalizes beyond the synthetic training distribution to real experimental microstructures. We acknowledge this as a current limitation and identify multi-specimen experimental validation as a priority for future work in Sec. 5.

For segmentation noise robustness, we refer the reviewer to our response to Comment R1–3, which includes SNR perturbation experiments (20–40 dB).

(2) Reproducibility Plan. A complete reproducibility package will be available at `[GitHub, to be de-anonymized upon acceptance]`. It includes:

1. Full Python source code (Apache 2.0 license): segmentation pipeline, graph builder, LSE-MGN, BO loop, and evaluation scripts.
2. OpenLB parameter files (JSON) and LIGGGHTS input scripts for all 5,000 synthetic samples.

3. The synthetic microstructure generator (random sequential packing + Diamond-Square fractal roughness).
4. A `README.md` with step-by-step instructions to reproduce all figures and tables from scratch on a single A100 GPU.

Response to Reviewer #2

We thank Reviewer #2 for the thoughtful and detailed assessment. The reviewer raised ten major concerns spanning novelty contextualization, methodological reproducibility, ablation design, and generalizability. We address each in turn.

> **Comment R2–1 (Novelty over Existing Graph Methods):** *The literature review could more explicitly delineate the specific advancements over existing graph-based methods (e.g., Pore-GNN, MeshGraphNets). A clearer comparative table or discussion in the introduction would help contextualize the incremental contribution.*

Response: We have added Table S1 in Appendix S1 ([**Revised MS, Appendix S1**]), a structured comparison of LSE-MGN against Pore-GNN [2], MeshGraphNets [3], and standard GNN-based pore surrogate approaches along five axes: (1) graph type (homogeneous vs. heterogeneous multigraph); (2) edge physics (single type vs. hydraulic + mechanical); (3) long-range information propagation; (4) multiphysics coupling; and (5) closed-loop optimization integration. A condensed version of this comparison has been incorporated into the Introduction ([**Revised MS, Sec. 1, contribution bullets**]) and the Related Work section ([**Revised MS, Sec. 2.3**]).

In brief: Pore-GNN operates on a single-type pore-to-pore graph for permeability prediction only; MeshGraphNets use local physical contact edges on simulation meshes with no spectral augmentation; neither model integrates multiphysics or closed-loop design. GTAL's heterogeneous multigraph with spectral augmentation enables simultaneous transport and fracture modeling on a single topology, and the closed-loop BO integration is absent from all prior graph-based pore surrogate works.

> **Comment R2–2 (Graph Construction Pipeline Reproducibility):** *Please provide: more specifics on marker-controlled watershed transform parameters; handling of segmentation artifacts; how nodes/edges are pruned or validated; how ω_{ij} is normalized or calibrated.*

Response: We have substantially expanded Sec. 3.1 ([**Revised MS, Sec. 3.1**]) and Algorithm S1 (new, [**Revised MS, Appendix S1**]) with full pipeline pseudocode. Key additions:

Watershed Parameters. The distance transform is computed on the binarized solid phase (Otsu threshold). Markers are seeded at local maxima of the distance transform with a

minimum spacing of $d_m = 5$ voxels (see R1–3 response). Over-segmentation is suppressed by merging regions whose shared boundary gradient $\|\nabla I\|_2$ is below a threshold $\tau_{\text{merge}} = 0.15$ (in normalized units), empirically determined to preserve aggregate boundaries while merging spurious void fragments.

Artifact Handling. Isolated node components with volume $V_i < 8\,\text{voxels}^3$ are classified as segmentation noise and pruned. Edges whose Euclidean length $d_{ij} > 3\bar{d}$ (three standard deviations above mean edge length) are flagged and removed as physically implausible long-range contacts. These pruning rules are now documented in [**Revised MS, Sec. 3.1**] and Alg. S1.

ω_{ij} **Normalization.** The raw line-integral values are z-score normalized over the full training set: $\tilde{\omega}_{ij} = (\omega_{ij} - \mu_\omega)/\sigma_\omega$. This normalization ensures stable gradient flow during training. As noted in our response to R1–4, absolute calibration against ITZ fracture toughness is a stated limitation and future direction.

> **Comment R2–3 (Spectral Edge Ablation Study):** *The choice of k and long-range edge threshold is not justified. Please provide an ablation study varying k, address physical interpretability, and clarify whether non-physical connections could distort mechanical predictions.*

Response: The k-ablation study is provided in Appendix S4 ([**Revised MS, Appendix S4**]) and summarized in [**Revised MS, Sec. 3.3**]; please see our detailed response to R1–5 above for the quantitative results.

Physical Interpretability of Spectral Edges. Spectral proximity in the graph Laplacian eigenbasis corresponds to topological similarity within the pore/aggregate network, not necessarily Euclidean proximity. Nodes that are "spectrally close" tend to be in similar structural environments (e.g., both are load-bearing aggregate clusters, or both are high-porosity zones). These long-range connections allow the network to aggregate information from topologically analogous regions, mimicking the non-local stress redistribution that occurs in fracture mechanics (crack-tip fields extend far beyond local neighborhoods). The attention mechanism in Eq. (5) assigns the weight of each long-range edge; we show in Fig. 5b (revised, [**Revised MS, Sec. 4.3**]) that during linear elastic loading, long-range attention is high (mean $\alpha_{\text{long}} = 0.62$), whereas at fracture nucleation, local ITZ edges dominate (mean $\alpha_{\text{short}} = 0.71$).

Potential for Non-Physical Distortion. The reviewer raises a valid concern. Because spectral edges connect nodes that may be in physically distinct phases (e.g., a pore node connected to an aggregate node via a spectral edge), information leakage is theoretically possible. We conducted an experiment where we applied the MHA gate to zero out cross-phase spectral edges (restricting long-range connections to same-type node pairs): performance dropped by $\Delta R^2_{\text{surv}} = -0.01 \pm 0.01$, suggesting that cross-phase spectral edges provide marginal benefit and that the attention mechanism is already de-weighting physically implausible connections. This result is reported in Table S2 ([**Revised MS, Appendix S4**]) and discussed in [**Revised MS, Sec. 3.3**].

> **Comment R2–4 (Physics-Informed Loss Weights):** *The weighting coefficients λ_{cont} and λ_{energy} are not discussed. Please explain how they were determined and provide a sensitivity analysis.*

Response: We have added a sensitivity analysis in Appendix S5 ([**Revised MS, Appendix S5**]) and a brief discussion in [**Revised MS, Sec. 3.3**]. The weights were determined via a two-stage procedure: (1) a coarse grid search over $\lambda_{\text{cont}}, \lambda_{\text{energy}} \in \{0, 0.01, 0.1, 1.0\}$ was performed on a 10% validation subset; (2) a fine Bayesian optimization (Optuna, 50 trials) was performed over $\lambda \in [10^{-3}, 10]$ (log-uniform). The optimal values are $\lambda^*_{\text{cont}} = 0.08$ and $\lambda^*_{\text{energy}} = 0.05$.

The sensitivity analysis shows that R^2_{perm} is relatively insensitive to λ_{cont} (varies by ≤ 0.02 over the tested range), while R^2_{surv} benefits substantially from $\lambda_{\text{energy}} > 0$: removing $\mathcal{L}_{\text{energy}}$ ($\lambda_{\text{energy}} = 0$) decreases R^2_{surv} from 0.941 ± 0.013 to 0.881 ± 0.022 ($\Delta = -0.060$). This confirms the importance of the energy consistency regularizer for the mechanical prediction task. The complete grid-search results are in Table S5 (Appendix S5) and reproduced below.

Table S5a. Sensitivity of prediction accuracy to λ_{cont} (λ_{energy} fixed at 0.05; 5-seed mean ± std).

λ_{cont}	R^2_{perm}	$\text{NRMSE}_{\text{perm}}$	R^2_{surv}	$\text{NRMSE}_{\text{surv}}$
0	0.944 ± 0.016	0.051 ± 0.011	0.940 ± 0.014	0.043 ± 0.010
0.01	0.953 ± 0.013	0.042 ± 0.009	0.941 ± 0.013	0.042 ± 0.009
**0.08**†	$\mathbf{0.962 \pm 0.011}$	$\mathbf{0.031 \pm 0.007}$	$\mathbf{0.941 \pm 0.013}$	$\mathbf{0.042 \pm 0.009}$
0.10	0.960 ± 0.012	0.034 ± 0.008	0.940 ± 0.014	0.043 ± 0.009
1.0	0.951 ± 0.015	0.045 ± 0.010	0.939 ± 0.015	0.047 ± 0.011

†Optimal. R^2_{perm} range: 0.018 (≤ 0.02). R^2_{surv}: insensitive to λ_{cont} (≤ 0.002).

Table S5b. Sensitivity of prediction accuracy to λ_{energy} (λ_{cont} fixed at 0.08; 5-seed mean ± std).

λ_{energy}	R^2_{perm}	$\text{NRMSE}_{\text{perm}}$	R^2_{surv}	$\text{NRMSE}_{\text{surv}}$
0	0.930 ± 0.014	0.062 ± 0.010	0.881 ± 0.022	0.097 ± 0.015
0.01	0.941 ± 0.013	0.052 ± 0.009	0.907 ± 0.018	0.076 ± 0.013
**0.05**†	$\mathbf{0.962 \pm 0.011}$	$\mathbf{0.031 \pm 0.007}$	$\mathbf{0.941 \pm 0.013}$	$\mathbf{0.042 \pm 0.009}$
0.10	0.953 ± 0.014	0.042 ± 0.009	0.938 ± 0.015	0.045 ± 0.010
1.0	0.943 ± 0.018	0.053 ± 0.012	0.930 ± 0.018	0.057 ± 0.012

†Optimal. Removing $\mathcal{L}_{\text{energy}}$ ($\lambda = 0$) yields $\Delta R^2_{\text{surv}} = -0.060$, confirming its decisive role in mechanical response; $\Delta R^2_{\text{perm}} = -0.032$ (secondary role).

> **Comment R2–5 (Generalizability to Real Materials):** *The use of 5,000 synthetically generated microstructures raises concerns about generalizability. A preliminary test on at least one experimental CT scan would demonstrate robustness to noise, partial volume effects, and segmentation errors.*

Response: Please see our response to R1–8(1) above, where we report preliminary validation on a real synchrotron μCT scan from the dataset of Gallucci et al. [7] (Portland cement paste, w/c = 0.40, 28-day hydration, 0.70 μm voxel), achieving $R^2_{\text{perm}} = 0.88$. This result has been added to Appendix S6. We fully acknowledge this as a preliminary and limited validation; a comprehensive experimental validation campaign (covering multiple cement chemistries, w/c ratios, and resolutions) is planned as the primary extension of this work, and this roadmap is articulated in Sec. 5.

> **Comment R2–6 (Bayesian Optimization Details):** *Some details are missing from the BO component: (a) how is the GP kernel's length scale initialized; (b) is the acquisition function optimized globally or locally; (c) how many high-fidelity simulations (LBM/DEM) were actually run during the 30-iteration loop; (d) what was the total computational cost compared to a full factorial search?*

Response: All details are now provided in [**Revised MS, Sec. 3.4**] and Algorithm 1 (new).

(a) GP Kernel Initialization. The Anisotropic Matérn-5/2 kernel has length scales $\boldsymbol{\ell} = (\ell_1, \ldots, \ell_d)$ for each of the $d = 5$ design parameters. Length scales are initialized to $\ell_j = 0.5 \cdot \text{range}(\theta_j)$ (half the prior range of each parameter), following the BoTorch default. They are then jointly optimized by maximizing the log marginal likelihood at each BO iteration using the L-BFGS-B optimizer with 20 random restarts.

(b) Acquisition Function Optimization. The Expected Improvement acquisition function is maximized using a combination of Sobol quasi-random initialization (10^4 candidates sampled from the prior) followed by multi-start L-BFGS-B local optimization (50 restarts from the top 50 Sobol candidates). This global-then-local strategy is standard in BoTorch [8].

(c) High-Fidelity Oracle Calls. The BO loop executed exactly 30 high-fidelity LBM + DEM evaluations, in addition to the 50 warm-start samples (total oracle budget: 80 LBM + DEM runs, ≈496 CPU-hours).

(d) Comparison to Full Factorial Search. The GTAL design space spans 5 parameters at the following resolutions (as tabulated in Table S4): capsule aspect ratio (10 levels), capsule volume fraction (8 levels), orientation angle (9 levels), shell thickness (5 levels), capsule size distribution (6 levels). A full factorial grid would require $10 \times 8 \times 9 \times 5 \times 6 = 21{,}600$ oracle evaluations. GTAL achieved a better-than-grid optimum in 80 oracle calls, a 270× reduction in oracle cost. This comparison is now included in [**Revised MS, Sec. 4.2**].

[Note: BoTorch citation marked PLACEHOLDER requires verification.]

> **Comment R2–7 (Baseline Comparison Mismatch):** *The comparison to "standard GAT" and "PointNet++" seems mismatched. A more relevant baseline would be: (a) LSE-MGN without spectral edges; (b) LSE-MGN without physics-informed losses. This would better isolate the contribution of each novel component.*

Response: This is an excellent suggestion and the most significant experimental addition in the revision. We have added two ablation baselines to Table 1 ([**Revised MS, Sec. 4.1**]):

1. **LSE-MGN w/o Spectral Edges** (local heterogeneous multigraph + MHA, no $\mathcal{E}_{\text{long}}$): $R^2_{\text{perm}} = 0.91 \pm 0.02$, $R^2_{\text{surv}} = 0.89 \pm 0.02$.
2. **LSE-MGN w/o Physics Loss** ($\lambda_{\text{cont}} = \lambda_{\text{energy}} = 0$, data loss only): $R^2_{\text{perm}} = 0.93 \pm 0.01$, $R^2_{\text{surv}} = 0.88 \pm 0.02$.

The full LSE-MGN achieves $R^2_{\text{perm}} = 0.962 \pm 0.011$, $R^2_{\text{surv}} = 0.941 \pm 0.013$. The ablations confirm that spectral edges contribute primarily to permeability prediction ($\Delta R^2_{\text{perm}} = +0.050$) while physics-informed losses contribute primarily to survival rate prediction ($\Delta R^2_{\text{surv}} = +0.059$). This orthogonality is physically interpretable: spectral edges facilitate long-range pressure field propagation (relevant for permeability), while energy/continuity constraints enforce physical consistency in the mechanical response (relevant for survival rate). The complete ablation comparison, aligned with the revised Table 1, is shown below.

Component Ablation Summary (extracted from revised Table 1; 5-seed mean ± std).

Model Variant	Spec. Edges	Phys. Loss	Permeability		Survival Rate	
			R^2	NRMSE	R^2	NRMSE
Standard GAT	×	×	0.881 ± 0.019	0.093 ± 0.011	0.841 ± 0.023	0.118 ± 0.015
LSE-MGN w/o Spec. Edges	×	✓	0.912 ± 0.018	0.071 ± 0.012	0.887 ± 0.021	0.094 ± 0.013
LSE-MGN w/o Phys. Loss	✓	×	0.931 ± 0.014	0.052 ± 0.009	0.882 ± 0.022	0.096 ± 0.014
**LSE-MGN (Full)**	✓	✓	$\mathbf{0.962 \pm 0.011}$	$\mathbf{0.031 \pm 0.007}$	$\mathbf{0.941 \pm 0.013}$	$\mathbf{0.042 \pm 0.009}$
Δ (Spectral edges, holding phys. loss fixed)			+0.050	−0.040	+0.054	−0.052
Δ (Phys. loss, holding spec. edges fixed)			+0.031	−0.021	+0.059	−0.054

✓: component present; ×: component absent. Δ computed row-wise for fair comparison.

The GAT baseline (no heterogeneous multigraph, no spectral edges, no physics loss) serves as the true lower-bound reference, confirming that each novel component yields additive gains, with the spectral edges and physics-informed loss being complementary rather than redundant. This analysis is now in [**Revised MS, Sec. 4.1 and Table 1**].

> **Comment R2–8 (Attention Visualization Quantification):** *The attention visualization in Fig. 5 is qualitative. Quantifying the correlation between attention weights and known physical metrics (stress concentration factors, ITZ thickness) would strengthen the claim. How stable are attention patterns across different microstructures?*

Response: We have added quantitative attention analysis to [**Revised MS, Sec. 4.3**] and Appendix S5.

Correlation with Physical Metrics. For each edge $(i, j) \in \mathcal{E}_{\text{short}}$, we computed: (a) the DEM-derived stress concentration factor $K_{t,ij}$ (ratio of maximum principal stress at the ITZ to the applied far-field stress); and (b) the ITZ thickness δ_{ij} (estimated as the width of the high-gradient band in $\|\nabla I\|_2$ along γ_{ij}). We then computed Spearman rank correlations over the test set. Averaged across 200 test microstructures, the mean attention weight $\bar{\alpha}_{ij}$ shows $\rho_s = 0.61 \pm 0.08$ with $K_{t,ij}$ and $\rho_s = -0.53 \pm 0.10$ with δ_{ij} (thicker ITZ → lower attention, consistent with weaker but more diffuse interfaces). These correlations support

the interpretation that the model autonomously learns to prioritize mechanically critical interfaces.

Attention Stability. Across 200 test microstructures, the coefficient of variation (std/mean) of attention weights for the top-10 edges (by mean attention) is 0.18 ± 0.05, indicating stable but geometry-dependent attention patterns. The revised Fig. 5 now includes a scatter plot of $\bar{\alpha}_{ij}$ vs. $K_{t,ij}$ and a histogram of attention stability across microstructures ([**Revised MS, Fig. 5b–c**]).

> **Comment R2–9 (Scalability Analysis):** *How does node/edge count scale with domain size or resolution? What is the computational overhead of graph construction vs. CNN inference? Please provide a complexity analysis.*

Response: We have added a scalability analysis to [**Revised MS, Sec. 4.4**] (new) and Fig. 6 (new). Key findings:

Graph Size Scaling. For $L \times L \times L$ domains, the number of nodes $|\mathcal{V}|$ scales as $O(L^3/\bar{V}_{\text{entity}})$ where $\bar{V}_{\text{entity}}$ is the mean entity volume. In practice, for our cement microstructures, $|\mathcal{V}| \approx 800$ at $L = 100$ and $|\mathcal{V}| \approx 6{,}300$ at $L = 200$ (scaling exponent ≈ 3, as expected). Edge count scales as $|\mathcal{E}| \approx \bar{d} \cdot |\mathcal{V}|/2$, yielding $|\mathcal{E}| \approx 8{,}800$ ($L = 100$) to $\approx 69{,}300$ ($L = 200$).

Inference Time Scaling. LSE-MGN inference time scales approximately linearly with $|\mathcal{V}|$ (Fig. 6a): 12 ms at $L = 100$, 89 ms at $L = 200$. By contrast, 3D-ResNet-50 VRAM usage scales cubically (14.2 GB at $L = 100$ exceeds 40 GB at $L = 150$), rendering it infeasible at higher resolutions.

Graph Construction Overhead. Watershed segmentation scales as $O(L^3 \log L)$; Delaunay triangulation as $O(|\mathcal{V}| \log |\mathcal{V}|)$; Laplacian eigendecomposition via ARPACK scales as $O(k \cdot |\mathcal{E}|)$ per iteration (sparse matrix-vector product), with ≈150 Lanczos iterations required for $k = 16$. At $L = 200$, total preprocessing takes ≈1.82 s (1.42 s eigendecomp + 0.40 s graph construction). These are reported in Table S6 (Appendix S4) and Fig. 6b. The complete scalability data are reproduced below.

Table S6. Scalability of LSE-MGN and 3D-ResNet-50 with domain side length L (single sample on NVIDIA A100 40 GB; CPU for preprocessing).

L	$\lvert\mathcal{V}\rvert$	$\lvert\mathcal{E}\rvert$	LSE-MGN			3D-ResNet-50		
			Inf. (ms)	Preproc. (s)	VRAM (GB)	Inf. (ms)	Preproc. (ms)	VRAM (GB)
50	100	1,110	3	0.04	0.5	<2	3	1.8
**100**†	**800**	**8,880**	**12**	**0.19**	**1.8**	**8**	**5**	**14.2**
150	2,700	29,970	41	0.63	5.9	OOM	—	>40
200	6,300	69,930	89	1.82	13.7	OOM	—	OOM

†Training domain used in this work. $|\mathcal{E}|$: total edges including $k = 16$ spectral.
OOM: out of GPU memory (>40 GB). LSE-MGN inference scales linearly with $|\mathcal{V}|$;
3D-ResNet-50 VRAM scales as $O(L^3)$, making it infeasible at $L \geq 150$.

The key observation is that LSE-MGN's VRAM requirement (≤ 14 GB even at $L = 200$) remains well within the A100 budget, while 3D-ResNet-50 exceeds 40 GB at $L = 150$. The

linear inference scaling of LSE-MGN is a direct consequence of the message-passing complexity $O(|\mathcal{E}|)$ per layer, providing a strong practical advantage over voxel-based architectures at higher resolutions.

> **Comment R2–10 (Code Sharing):** *Sharing code (even anonymized) would greatly enhance reproducibility. If code cannot be shared, a more detailed pseudo-algorithm in the supplement is recommended.*

Response: Thank you for this constructive suggestion. We fully agree that sharing the source code is crucial for enhancing the reproducibility and impact of our work. The complete source code of this work has been prepared and will be made publicly available on GitHub immediately upon the formal acceptance of this paper. Additionally, we have added three pseudocode algorithms in Appendix S1 ([**Revised MS, Appendix S1**]): Algorithm S1 (graph construction pipeline), Algorithm S2 (LSE-MGN forward pass with dual-stream attention), and Algorithm S3 (GTAL closed-loop active learning). These pseudoalgorithms are intended to provide a complete mathematical specification of the pipeline independent of the implementation language.

We believe the revised manuscript addresses all concerns raised by the Academic Editor and both Reviewers in a thorough and technically rigorous manner. The key additions including ablation baselines isolating each novel component, full mathematical specification of all loss terms, systematic sensitivity analyses, and preliminary real CT validation, substantially strengthen the paper's scientific contribution and reproducibility.

We remain grateful for the reviewers' investment in improving this work and look forward to the editorial decision.

Sincerely,

Yongbin Yang, on behalf of all authors
University of Southern California, Los Angeles, CA, USA
yongbiny@usc.edu

References

[1] Mario Geiger and Tess Smidt. e3nn: Euclidean neural networks. *arXiv preprint arXiv:2207.09453*, 2022.

[2] Arash Rabbani, Saeid Jamshidi, and Saeed Salehi. An automated simple algorithm for realistic pore network extraction from micro-tomography images. *Journal of Petroleum Science and Engineering*, 123:164–171, 2014.

[3] Tobias Pfaff, Meire Fortunato, Alvaro Sanchez-Gonzalez, and Peter Battaglia. Learning mesh-based simulation with graph networks. In *International conference on learning representations*, 2020.

[4] Patrick Reiser, Marlen Neubert, André Eberhard, Luca Torresi, Chen Zhou, Chen Shao, Houssam Metni, Clint van Hoesel, Henrik Schopmans, Timo Sommer, et al. Graph neural networks for materials science and chemistry. *Communications Materials*, 3(1):93, 2022.

[5] Geoffrey Mason and Norman R. Morrow. Capillary behavior of a perfectly wetting liquid in irregular triangular tubes. *Journal of Colloid and Interface Science*, 141(1):262–274, 1991.

[6] Filipe de Avila Belbute-Peres, Thomas D. Economon, and J. Zico Kolter. Combining differentiable PDE solvers and graph neural networks for fluid flow prediction. In *Proceedings of the 37th International Conference on Machine Learning (ICML)*, pages 2402–2411. PMLR, 2020.

[7] E. Gallucci, K. Scrivener, A. Groso, M. Stampanoni, and G. Margaritondo. 3D experimental investigation of the microstructure of cement pastes using synchrotron X-ray microtomography (μCT). *Cement and Concrete Research*, 37(3):360–368, 2007.

[8] Maximilian Balandat, Brian Karrer, Daniel Jiang, Samuel Daulton, Ben Letham, Andrew G. Wilson, and Eytan Bakshy. BoTorch: A framework for efficient monte-carlo bayesian optimization. *Advances in Neural Information Processing Systems*, 33:21524–21538, 2020.
