# Supplementary material for: Graph-theoretic active learning for the closed-loop discovery of stochastic heterogeneous composites
Source: PLoS One. 2026 Aug 18;21(8):e0353692. doi: 10.1371/journal.pone.0353692 (PMC13485134; doi:10.1371/journal.pone.0353692)
Supplement: Supplementary file 9 [file pone.0353692.s011.pdf]

Response to Reviewers

Journal: PLOS ONE • Manuscript ID: PONE-D-25-64550R1

Title: Graph-Theoretic Active Learning for the Closed-Loop Discovery of Stochastic Heterogeneous Composites

Dear Dr. Mohammad Reza Maghami and the Editorial Board of PLOS ONE,

We thank the Academic Editor and the reviewers for their continued evaluation of our manuscript. We are encouraged that Reviewer #4 and Reviewer #5 now find the work technically sound, statistically appropriate, the data fully available, and the language clear, and that Reviewer #5 recommends acceptance in its present form. We have used this round to (i) resolve the remaining quantitative-claim and terminology issues raised by Reviewer #4, (ii) provide the additional methodological justification, uncertainty discussion, and mechanistic verification requested by Reviewer #3, and (iii) sharpen, throughout, the distinction between what our framework demonstrates computationally and what remains for experimental confirmation.

All new second-round changes are highlighted in **dark red** in the revised manuscript, to distinguish them from the first-round changes, which remain in **blue**. For quick orientation, the most significant second-round revisions are:

- **Acceleration claim reconciled** (Reviewer #4.1): the abstract now reports both the end-to-end speedup (≈1.1×10^5-fold, inference + preprocessing) and the inference-only speedup (≈1.8×10^6-fold), and states explicitly that the end-to-end figure supersedes the inference-only number in the original submission metadata.
- **"Experimental" terminology corrected** (Reviewer #4.2,3): "experimental validation" has been replaced by "computational validation against high-fidelity numerical solvers" in the abstract, introduction, and throughout; the single μCT result is now framed explicitly as a preliminary, statistically limited proof-of-concept.
- **Optimization scope made explicit** (Reviewer #4.5,6; #3.5): a new "Scope of the optimization objective" paragraph clarifies that we optimize processing survival and the transport state of the host matrix, not the post-fracture healing cascade itself.
- **Mechanistic verification of the optimum** (Reviewer #3.5; #4.4): a new Appendix S7 derives the competing projected-shear-area and crack-interception cross-section analytically and shows the joint desirability is independently maximized at $\phi \approx 45°$, $\rho_c \approx 2.5$, in agreement with the data-driven optimum.
- **Material realism, uncertainty, soft-vs-hard constraints, and spectral scaling** (Reviewer #3.1-4; #4.6,7): DEM capsule rupture/bonding parameters are now stated; the residual-GP vs. GNN-native uncertainty choice is justified; the soft-penalty rationale under noisy DEM labels is given; and eigendecomposition scaling and the static-vs-dynamic-topology limitation are discussed.

We address every comment individually below. Reviewer comments are quoted in italics; manuscript locations are flagged in **red [Revised MS, ...]** tags.

Academic Editor: Journal Requirements

> **Editor:** *If the reviewer comments include a recommendation to cite specific previously published works, please review and evaluate these publications to determine whether they are relevant and should be cited. There is no requirement to cite these works unless the editor has indicated otherwise.*

Response: We thank the Editor for this guidance and have applied it carefully. The only explicit citation suggestions in this round appear in the comments labelled "Reviewer #2," which recommend three works on dual-active-bridge converters, hybrid modulation, and wind-energy systems (https://doi.org/10.1016/j.jestch.2019.08.006; https://doi.org/10.1016/j.susoc.2024.06.004; https://doi.org/10.3390/wind5010004). We reviewed all three. They concern power-electronics modulation and renewable-energy conversion and bear no methodological or topical relationship to graph-theoretic surrogate modelling of cementitious microstructure. Following the Editor's explicit instruction that there is no requirement to cite recommended works unless they are relevant, and to preserve the integrity of our reference list, we have **not** added these citations. We explain the apparent reviewer-manuscript mismatch under Reviewer #2 below. We did, in the previous round, substantially broaden the reference list with the equivariant-GNN, Pore-GNN, MeshGraphNets, and CT-validation literature directly relevant to this work; all references have been verified against CrossRef and Semantic Scholar.

Response to Reviewer #2

We thank Reviewer #2 for the time invested in a detailed review. We must, however, respectfully and with no criticism of the reviewer bring a likely administrative mismatch to the Editor's attention.

> **Reviewer #2 (excerpt):** *The manuscript titled "Design and Performance Analysis of a Simplified Hybrid Modulation Method for Dual Active Bridge Powering Inverters" proposes a simplified hybrid modulation strategy that integrates Single Phase Shift (SPS) and Triple Phase Shift (TPS) techniques for operating Dual Active Bridge (DAB) converters … validated through small-signal modeling, simulations, and experimental results using a 500 W DAB-inverter prototype.*

Response: The manuscript described in this review (a power-electronics study of a 500 W dual-active-bridge inverter using SPS/TPS hybrid modulation, a single PI controller, small-signal modeling, and a hardware prototype) is **not the manuscript under consideration here**. Our paper (PONE-D-25-64550R1) presents a graph-neural-network surrogate and Bayesian-optimization framework for the inverse design of self-healing cementitious composites. It contains no converter, no modulation scheme, no PI controller, no Eq. (6)/(7) of the kind referenced, and no 30%-rated-power switching threshold. The specific comments (switching-loss quantification in TPS vs. SPS, the 30% TPS→SPS threshold sensitivity, the ϕ symbol consistency in Eqs. 6-7, the "this pose a significant challenge" grammar instance, and the three power-electronics references) all pertain to that other manuscript. We therefore believe this review was filed against our submission in error, and we kindly ask the editorial office to confirm whether it was intended for a different paper.

Response: So that this round is nonetheless productive, we have acted on the two comments from this review that *could* apply generically to any manuscript:

- **Reference recency and breadth.** Although the three suggested DOIs are not topically relevant (see the Editor response above), we re-audited our bibliography for currency in our actual field. The reference list already includes work published from 2023 to 2025 on equivariant GNNs,

graph-based pore-flow surrogates, MeshGraphNets-style fracture learning, diffusion-based material generation, and Bayesian optimization for materials; no power-electronics references were added. In addition, this verification pass surfaced one mis-keyed citation, which we have corrected: the two mentions of "Pore-GNN" in the Contributions list and in the "Positioning Relative to Graph-Based Pore Surrogates" subsection now cite Alzahrani et al. (2023), the actual Pore-GNN paper (previously they pointed to Rabbani et al. 2014, a watershed-based pore-network extraction study). Rabbani et al. (2014) remains in the reference list and is now cited where it belongs, for the marker-controlled watershed pore-network extraction in the Methods [Revised MS, Sec. 1, Contributions; Sec. 2.6; Sec. 3.1]. The reference list itself is unchanged in content; only the citation keys and order of first appearance were corrected.

- **Language and grammar.** We performed a full proofreading pass of the revised manuscript. The specific error quoted by the reviewer ("this pose" → "this poses") does not occur in our text; we nonetheless corrected minor phrasing throughout for clarity, consistent with Reviewer #4's and Reviewer #5's assessment that the language is already clear and correct.

Response: We are happy to respond in full to any comments the editor confirms were intended for our manuscript.

Response to Reviewer #3

We thank Reviewer #3 for an exceptionally thorough and constructive assessment, and for recognizing the framework's innovation and computational performance. The reviewer raised six substantive points; we address each below, and we have added new text and a new Appendix (S7) in response.

Comment R3-1 (Sim-to-real gap and synthetic-data realism)

> **Reviewer #3:** *The reliance on entirely synthetic training data … introduces a substantial sim-to-real gap that is only superficially addressed through a single synchrotron microtomography validation (R^2 = 0.88) … the synthetic dataset completely neglects realistic capsule size distributions, curing-induced microstructural evolution, and the complex porosity gradients inherent to actual cement hydration.*

Response: We agree this is the central limitation and have addressed it on three fronts without overclaiming. (a) We added an explicit paragraph in the Dataset section **[Revised MS, Sec. 4.1, Dataset Generation]** stating exactly which real-microstructure statistics the synthetic generator reproduces (phase-volume fractions, two-point correlation length, aggregate-size distribution; porosity envelope 15% to 35% bracketing OPC capillary porosity at w/c ≈ 0.35 to 0.55) and which it does *not* (C-S-H gel pore evolution, chemically graded ITZ, capsule polydispersity beyond the sampled range, and segmentation/partial-volume artifacts). (b) We retained and reframed the synthetic-noise robustness study (Appendix S3), which quantifies how segmentation noise propagates through the graph-construction pipeline, directly the mechanism the reviewer identifies. (c) We reframed the μCT result (see R3-1 cont. and R4-9) as a preliminary consistency check rather than a generalization claim, and we elevated multi-specimen experimental validation to the first item of future work **[Revised MS, Sec. 5]**. We respectfully note that a full multi-chemistry experimental campaign or a domain-adaptation study, while clearly valuable, constitutes a separate experimental program; we have therefore been careful to state our claims at the level the present evidence supports rather than manufacturing additional results.

Comment R3-2 (Loss weighting; soft vs. hard constraints; interaction with DEM label noise)

> **Reviewer #3:** *The physics-informed loss functions … lack a deeper theoretical justification for their weighting scheme … the manuscript does not explore how these soft constraints interact with the stochastic noise inherent in DEM labeling or whether hard constraint enforcement via projection layers might yield more thermodynamically consistent predictions.*

Response: We added a dedicated discussion after the loss-weight specification **[Revised MS, Sec. 3.4, Physics-Informed Objective]**. The argument is twofold. First, on soft vs. hard constraints: because the DEM survival labels are themselves stochastic (sensitive to the packing realization), a hard projection onto the strain-energy-compatible manifold would force the network to fit physically meaningless per-capsule label noise; in preliminary trials a projection-layer variant reduced R^2(surv) by ≈ 0.02 while roughly doubling training time. The soft penalty instead behaves as a Tikhonov-type regularizer whose strength is matched to the label signal-to-noise ratio, which is precisely what the λ sensitivity sweep (Appendix S5) calibrates. Second, the small optimal magnitudes ($\lambda^* < 0.1$) are consistent with this interpretation: the residual terms shape the loss landscape and prune non-physical minima rather than overriding the empirical fit in the presence of noisy labels. We believe this provides the theoretical grounding the reviewer requested while remaining faithful to the experiments we actually ran.

Comment R3-3 (GNN epistemic uncertainty vs. GP-on-residuals; kernel conditioning)

> **Reviewer #3:** *The Bayesian optimization loop treats the GNN as a deterministic surrogate and models residuals using a Gaussian process … integrating ensemble-based or Monte Carlo dropout uncertainty directly into the acquisition function would provide a more principled exploration–exploitation balance … the anisotropic Matérn-5/2 kernel length scales may become poorly conditioned in higher-dimensional synthesis spaces.*

Response: We added a paragraph in the Bayesian-optimization section **[Revised MS, Sec. 3.5]** giving both the rationale and its limit. In our low-dimensional setting ($d = 5$ continuous parameters) a residual GP is the standard, well-conditioned choice: it gives closed-form posterior variance and analytic Expected Improvement, and it cleanly separates exploration in the 5-D parameter space from the much higher-dimensional graph-feature space in which GNN-native uncertainty would live. We verified the residual GP is not the bottleneck by comparing against a 5-member deep-ensemble acquisition on the warm-start pool: the two strategies selected overlapping candidates and reached the same optimum within the run-to-run variance of Fig. 4, so we retained the simpler GP. On conditioning: with $d = 5$ the fitted covariance had condition number $< 10^4$ throughout and the anisotropic length scales remained well separated, so we did not observe premature convergence. We agree that for substantially higher-dimensional spaces ($d \geq 20$) a GNN-native uncertainty integrated into the acquisition function would be the more principled choice, and we now flag this in Sec. 5.

Comment R3-4 (Spectral preprocessing scaling; dynamic topology during crack propagation)

> **Reviewer #3:** *The spectral long-range edge augmentation … introduces a preprocessing overhead of ≈180 ms per graph due to eigendecomposition, and the manuscript does not adequately discuss how this scales to industrial-resolution domains exceeding 500^3 voxels or how dynamic topological*

> *changes during crack propagation would be handled in a time-dependent setting.*

Response: We added two scaling caveats to the Scalability Analysis **[Revised MS, Sec. 4.7]**. (a) Spectral preprocessing is the least-favorably-scaling component: the partial eigendecomposition (ARPACK, k = 16) grows roughly as O(|E|·k), reaching 1.82 s at L = 200. For domains beyond 500^3 voxels we identify the established remedy, a hierarchical or locally-windowed spectral construction (multiscale or Nyström approximation of the eigenbasis), which preserves long-range connectivity at near-linear preprocessing cost; we are explicit that we have not yet implemented it. Because eigendecomposition is one-time preprocessing and is excluded from the inference-time speedup, it does not affect the reported ≈ 1.8×10^6-fold inference acceleration, but it does bound practical domain size in the current implementation. (b) On dynamic topology: the present surrogate models static-equilibrium permeability and processing survival of a fixed graph; capturing the time-dependent diffusive-reactive healing process (topology changing as cracks open and seal) requires a temporal/autoregressive extension with edges added/removed between message-passing steps, analogous to dynamic-graph MeshGraphNet variants. We treat this as a distinct, substantial extension (Sec. 5) rather than a parameter change.

Comment R3-5 (Mechanistic validation of the discovered optimum)

> **Reviewer #3:** *The claim that a 45-degree orientation minimizes projected cross-sectional area during mixing while simultaneously maximizing crack interception probability must be explicitly quantified through probabilistic fracture mechanics analysis or discrete crack tracking … to confirm that the identified optimum genuinely resolves the conflicting constraints rather than exploiting a statistical artifact.*

Response: This is an excellent point and we have addressed it directly with a new **[Revised MS, Appendix S7]** and a pointer in the Results **[Revised MS, Sec. 4.4]**. Appendix S7 derives the two competing geometric quantities analytically for a prolate spheroid under an iso-volume constraint: the orientation-dependent projected shear area $A_\perp(\phi, \rho c)$ governing survival, and the orientation-averaged crack-interception caliper $\langle \ell int \rangle(\phi, \rho c)$ governing healing. Crucially, neither objective alone has an interior optimum (survival drives $\phi \to 0$; interception drives large aligned ρc), but their product $S = [1 - A_\perp/Amax]\cdot\langle \ell int \rangle$ does, because 45° uniquely balances the competing sinϕ (area growth) and cosϕ (interception) dependencies while the $\rho c^{-2/3}$ iso-volume penalty caps elongation. Numerically maximizing over the design box gives $\phi^* \approx$ 43-47° and $\rho c^* \approx$ 2.2 to 2.8 (Table S8), coinciding with the optimum the GTAL loop discovered from data. This provides independent mechanistic support that the discovered configuration resolves rather than circumvents the conflict. We are careful to label this a reduced geometric/mechanical argument and to retain capsule fabrication and crack-interception testing as the experimental confirmation in Sec. 5.

Comment R3-6 (Reproducibility: MHA hyperparameters, ω_ij formulation, boundary conditions; equivariant baseline)

> **Reviewer #3:** *The manuscript … omits critical details regarding hyperparameter selection for the multi-head attention mechanism, the exact formulation of the interface weakness metric ω_ij, and the boundary condition implementations … the comparison baselines should include at least one state-of-the-art equivariant graph network trained on identical heterogeneous inputs.*

Response: We have consolidated the reproducibility details. (a) **MHA / training configuration:** a new

paragraph **[Revised MS, Sec. 3.3, Dual-Stream Message Passing]** fixes hidden dimension d = 128, L_mp = 6 layers, 4 attention heads (head dim 32), attention dropout 0.1, LayerNorm residual fusion, AdamW (lr 10^{-3}, weight decay 10^{-5}), batch size 16, cosine annealing, and early stopping (patience 20). These are also given step-by-step in Algorithm S2. (b) **ω_ij:** the exact discrete formulation (Sobel-gradient line integral along the Dijkstra solid-phase path with z-score normalization) is in the Methods and Algorithm S1. (c) **Boundary conditions:** the inlet/outlet pressure and bottom/top displacement implementations for L_cont and L_energy are specified in Appendix S1 and illustrated in Fig. S1. (d) **Equivariant baseline:** as requested, an SE(3)-equivariant E3NN network trained on identical inputs is included in Tables 1 and 2 (R^2_perm = 0.872, R^2_surv = 0.823), underperforming LSE-MGN; we explain this by the system's lack of global isotropy (preferred shear/gravity/casting directions), which makes hard global-symmetry enforcement suboptimal **[Revised MS, Sec. 4.2]**.

Response to Reviewer #4

We thank Reviewer #4 for confirming the manuscript is technically sound, statistically appropriate, the data fully available, and the language clear, and for nine precise comments that have measurably improved the calibration of our claims. Several comments share a common theme, the distinction between computational and experimental validation, which we have addressed consistently throughout.

Comment R4-1 (Acceleration-claim inconsistency)

> **Reviewer #4:** *The abstract contains an inconsistency in acceleration claims. The submission metadata mentions 500-fold acceleration, while the manuscript abstract reports approximately 1.1 × 10^5-fold acceleration. These claims must be made consistent.*

Response: We have made the claims fully consistent and self-explaining in the abstract **[Revised MS, Abstract]**, and in Sec. 4.6 and Table 2. The two numbers refer to different denominators: the **inference-only** speedup (GNN forward pass vs. LBM+DEM, excluding one-time graph construction) is ≈ 1.8×10^6-fold, while the **end-to-end** speedup (inference + 190 ms preprocessing) is ≈ 1.1×10^5-fold. The abstract now reports both and states explicitly that the end-to-end figure supersedes the inference-only number that appeared in the original submission metadata. We report the more conservative end-to-end figure as the headline claim everywhere in the paper.

Comment R4-2 ("Experimental validation" overstated)

> **Reviewer #4:** *The term "experimental validation" is overstated. Most validation appears to be based on synthetic microstructures and high-fidelity numerical simulations. Only one real synchrotron μCT specimen is mentioned, so the word "experimental" should be used cautiously.*

Response: Agreed. We replaced "experimental validation" with "**computational validation against high-fidelity numerical solvers**" in the abstract and the Introduction roadmap **[Revised MS, Abstract; Sec. 1]**, and we reserve "experimental" strictly for the single real μCT scan, which we now label a preliminary proof-of-concept (see R4-9). The Results section headers refer to computational/numerical validation throughout. During a final consistency check of the compiled manuscript we located and corrected three residual instances that had escaped the earlier pass: the Results overview now reads "computational evaluation campaign" (previously "experimental

campaign") and "computationally validated composite design" (previously "physically validated composite design"), and the convergence-figure caption now refers to "optimization runs" rather than "experimental runs" [Revised MS, Sec. 4, opening paragraphs; Fig 4 caption].

Comment R4-3 (Study is simulation-driven, not experimentally verified)

> **Reviewer #4:** *The study is mainly simulation-driven, not experimentally verified. No physical fabrication, mechanical testing, permeability testing, or self-healing performance test of the optimized capsule configuration is presented.*

Response: We accept this characterization and have made it explicit rather than implicit. The new “Scope of the optimization objective” paragraph **[Revised MS, Sec. 4.1]** and the expanded limitations **[Revised MS, Sec. 5]** state plainly that the framework is validated against numerical oracles (LBM/DEM) plus one real μCT scan, and that physical fabrication, mechanical/permeability testing, and direct self-healing performance tests of the optimized configuration are future work. We have not added any claim of physical testing.

Comment R4-4 (Optimized design claim needs physical confirmation)

> **Reviewer #4:** *The conclusion that anisotropic microcapsules with aspect ratio around 2.5 and 45° orientation are optimal should be validated using additional DEM/LBM cases and, ideally, experimental capsule survival / crack-interception tests.*

Response: We have strengthened the support for the optimum in two ways that do not overstate the evidence. (a) New Appendix S7 provides an independent analytical confirmation that the survival×interception desirability is maximized at $\phi \approx 45°$, $\rho c \approx 2.2$ to 2.8 (Table S8), derived from first-principles geometry rather than the surrogate. (b) We explicitly retain experimental capsule-survival / crack-interception testing as a priority future experiment **[Revised MS, Sec. 5]**. We have phrased the Results and Abstract so that the 45°/2.5 configuration is described as a model-predicted optimum corroborated analytically, not an experimentally confirmed one. (c) For full consistency with the recalibrated abstract, the Conclusion now likewise states that the discovered configuration is "predicted to resolve" the conflicting objectives, citing the analytical corroboration of Appendix S7 explicitly [Revised MS, Sec. 5].

Comment R4-5 (Healing-performance objective is indirect)

> **Reviewer #4:** *The framework optimizes permeability and microcapsule survival rate, but actual healing efficiency, crack closure, healing-agent release, transport, polymerization, or strength recovery is not directly modeled.*

Response: Correct, and we now state this directly in the new “Scope of the optimization objective” paragraph **[Revised MS, Sec. 4.1]**: permeability quantifies transport resistance of the uncracked matrix and survival quantifies mechanical robustness through mixing; the post-fracture healing cascade (rupture, release, capillary transport, polymerization, strength/permeability recovery) is a time-dependent diffusive-reactive process outside the static-equilibrium surrogate. We connect this to the dynamic-graph extension proposed in response to R3-4 and listed in Sec. 5, where healing efficiency itself, rather than its survival/transport proxies, becomes the objective.

Comment R4-6 (Self-healing mechanism needs material realism)

> **Reviewer #4:** *Microcapsule shell material, healing-agent viscosity, capsule rupture threshold, shell thickness, capsule–matrix bonding, and post-crack release behavior should be defined more clearly.*

Response: We added the DEM material parameterization to the Dataset section **[Revised MS, Sec. 4.1, Ground-truth labeling]**: a maximum von-Mises rupture threshold σ_{rup} = 4.0 MPa (representative of urea-formaldehyde shells), a cohesive capsule-matrix contact with normal/tangential stiffness k_n = 1.0×10^8 N m^{-1}, and a shell-to-diameter thickness ratio of 0.08. We are explicit that these are nominal literature-derived values held fixed across the dataset (not measurements on a specific cement chemistry), so survival is a mechanical-robustness proxy under a single representative parameter set. Post-crack release behavior is part of the healing cascade we do not model (R4-5) and is deferred to the dynamic extension in Sec. 5.

Comment R4-7 (Synthetic dataset generation needs stronger justification)

> **Reviewer #4:** *The 5,000 microstructures are generated using random sequential packing and Diamond-Square pore synthesis, but it is unclear how closely these synthetic structures represent real cement paste or concrete microstructures.*

Response: Addressed by the new realism paragraph **[Revised MS, Sec. 4.1]** described under R3-1: it states which statistics the generator reproduces (phase fractions, two-point correlation length, aggregate-size distribution; porosity 15% to 35% bracketing OPC capillary porosity at w/c ≈ 0.35 to 0.55) and which it omits (gel-pore evolution, graded ITZ, capsule polydispersity, segmentation artifacts), and frames the synthetic corpus as a controlled, physics-labeled training set rather than a substitute for real microstructures.

Comment R4-8 (Sim-to-real gap remains a major limitation)

> **Reviewer #4:** *Validation on one real μCT scan is not sufficient to claim robust generalization … More real datasets with different w/c ratios, curing ages, resolutions, and segmentation noise are needed.*

Response: We agree and have removed any language implying robust real-data generalization. The single-scan result is now explicitly a qualitative consistency check **[Revised MS, Sec. 4.8 and Appendix S6]**, and a multi-specimen campaign spanning w/c ratios, curing ages, resolutions, and segmentation-noise levels is now the first listed priority in Sec. 5. The segmentation-noise robustness study (Appendix S3) provides a controlled, synthetic-side proxy for one axis of that gap in the interim.

Comment R4-9 (Real μCT validation is statistically weak)

> **Reviewer #4:** *The manuscript reports R^2 for only three orthogonal permeability values from one specimen. Such a small validation set cannot support strong claims about real-data generalization.*

Response: We fully accept this and have rewritten the relevant text **[Revised MS, Sec. 4.8]** to state that R^2 = 0.88 over three directional permeabilities from one specimen is a qualitative consistency check, showing correct anisotropy ordering (X > Y > Z) and ≈10% directional error, and explicitly **not** a statistically powered validation. We no longer use it to support any generalization claim; it is presented solely as proof-of-concept that the synthetic-to-real gap is tractable for permeability, with the multi-specimen study (R4-8) identified as the necessary follow-up.

Response to Reviewer #5

> **Reviewer #5:** *Accept in the present form. The authors have satisfactorily addressed all the comments and therefore recommended for acceptance. (All comments have been addressed; manuscript technically sound; statistics appropriate; data available; language clear.)*

Response: We sincerely thank Reviewer #5 for the positive assessment and the recommendation to accept. We are grateful for the reviewer's careful evaluation across the previous rounds, which materially improved the manuscript. No further changes were requested; the second-round revisions above (made in response to Reviewers #3 and #4) further strengthen the claim calibration and reproducibility while leaving the core results and conclusions unchanged.

Summary

We have reconciled the acceleration claims, corrected the "experimental" terminology, made the optimization scope explicit, added an analytical mechanistic verification of the discovered optimum (Appendix S7), specified the DEM material parameters, justified the loss-weighting and uncertainty-modeling choices, discussed spectral-preprocessing scaling and the static-vs-dynamic-topology limitation, and reframed the single-specimen μCT result honestly as a preliminary consistency check. We have not added any unsupported experimental results; where a request requires a new experimental campaign, we have calibrated our claims accordingly and identified the work as a clearly scoped future direction. We also flagged the apparent reviewer-manuscript mismatch in the Reviewer #2 comments for the editorial office's attention. Finally, in the same proofreading pass we updated the Fig 5 caption to describe panels (b)-(c) (the attention-stress-concentration scatter and the attention-stability histogram added in the first revision), so that the caption matches the figure content and the quantitative analysis reported in Sec. 4.5 and Appendix S5.

We thank the Editor and all reviewers again for their time, and we hope the revised manuscript is now suitable for publication in PLOS ONE.

Sincerely,

Yongbin Yang, on behalf of all co-authors (L. Qiu, S. Zhang, M. Wang)
Corresponding author: yongbiny@usc.edu
