# Supplementary material for: Graph-theoretic active learning for the closed-loop discovery of stochastic heterogeneous composites
Source: PLoS One. 2026 Aug 18;21(8):e0353692. doi: 10.1371/journal.pone.0353692 (PMC13485134; doi:10.1371/journal.pone.0353692)
Supplement: Peer review history

# Peer review: Decision Letter 0

**DOI:** 10.1371/journal.pone.0353692.r001

**Authors:** Mohammad Maghami

4 Feb 2026

Dear Dr. Yang,

Thank you for submitting your manuscript to PLOS ONE. After careful consideration, we feel that it has merit but does not fully meet PLOS ONE’s publication criteria as it currently stands. Therefore, we invite you to submit a revised version of the manuscript that addresses the points raised during the review process.

Please submit your revised manuscript by Mar 21 2026 11:59PM. If you will need more time than this to complete your revisions, please reply to this message or contact the journal office at plosone@plos.org. When you're ready to submit your revision, log on to https://www.editorialmanager.com/pone/ and select the 'Submissions Needing Revision' folder to locate your manuscript file.

- A letter that responds to each point raised by the academic editor and reviewer(s). You should upload this letter as a separate file labeled 'Response to Reviewers'.
- A marked-up copy of your manuscript that highlights changes made to the original version. You should upload this as a separate file labeled 'Revised Manuscript with Track Changes'.
- An unmarked version of your revised paper without tracked changes. You should upload this as a separate file labeled 'Manuscript'.

If you would like to make changes to your financial disclosure, please include your updated statement in your cover letter. Guidelines for resubmitting your figure files are available below the reviewer comments at the end of this letter.

If applicable, we recommend that you deposit your laboratory protocols in protocols.io to enhance the reproducibility of your results. Protocols.io assigns your protocol its own identifier (DOI) so that it can be cited independently in the future. For instructions see: https://journals.plos.org/plosone/s/submission-guidelines#loc-laboratory-protocols. Additionally, PLOS ONE offers an option for publishing peer-reviewed Lab Protocol articles, which describe protocols hosted on protocols.io. Read more information on sharing protocols at https://plos.org/protocols?utm_medium=editorial-email&utm_source=authorletters&utm_campaign=protocols.

We look forward to receiving your revised manuscript.

Kind regards,

Mohammad Reza Maghami

Academic Editor

PLOS One

Journal Requirements:

When submitting your revision, we need you to address these additional requirements.

1. Please ensure that your manuscript meets PLOS ONE's style requirements, including those for file naming. The PLOS ONE style templates can be found at

https://journals.plos.org/plosone/s/file?id=wjVg/PLOSOne_formatting_sample_main_body.pdf and

https://journals.plos.org/plosone/s/file?id=ba62/PLOSOne_formatting_sample_title_authors_affiliations.pdf

2. Please note that PLOS One has specific guidelines on code sharing for submissions in which author-generated code underpins the findings in the manuscript. In these cases, we expect all author-generated code to be made available without restrictions upon publication of the work. Please review our guidelines at https://journals.plos.org/plosone/s/materials-and-software-sharing#loc-sharing-code and ensure that your code is shared in a way that follows best practice and facilitates reproducibility and reuse.

3. We note that your Data Availability Statement is currently as follows: [All relevant data are within the manuscript and its Supporting Information files.]

Please confirm at this time whether or not your submission contains all raw data required to replicate the results of your study. Authors must share the “minimal data set” for their submission. PLOS defines the minimal data set to consist of the data required to replicate all study findings reported in the article, as well as related metadata and methods (https://journals.plos.org/plosone/s/data-availability#loc-minimal-data-set-definition).

For example, authors should submit the following data:

- The values behind the means, standard deviations and other measures reported;

- The values used to build graphs;

- The points extracted from images for analysis.

Authors do not need to submit their entire data set if only a portion of the data was used in the reported study.

If your submission does not contain these data, please either upload them as Supporting Information files or deposit them to a stable, public repository and provide us with the relevant URLs, DOIs, or accession numbers. For a list of recommended repositories, please see https://journals.plos.org/plosone/s/recommended-repositories.

If there are ethical or legal restrictions on sharing a de-identified data set, please explain them in detail (e.g., data contain potentially sensitive information, data are owned by a third-party organization, etc.) and who has imposed them (e.g., an ethics committee). Please also provide contact information for a data access committee, ethics committee, or other institutional body to which data requests may be sent. If data are owned by a third party, please indicate how others may request data access.

4. If the reviewer comments include a recommendation to cite specific previously published works, please review and evaluate these publications to determine whether they are relevant and should be cited. There is no requirement to cite these works unless the editor has indicated otherwise.

[Note: HTML markup is below. Please do not edit.]

Reviewers' comments:

Reviewer's Responses to Questions

**Comments to the Author**

1. Is the manuscript technically sound, and do the data support the conclusions?

Reviewer #1: Partly

Reviewer #2: Yes

**********

2. Has the statistical analysis been performed appropriately and rigorously?

Reviewer #1: Yes

Reviewer #2: No

**********

3. Have the authors made all data underlying the findings in their manuscript fully available?

The PLOS Data policy

Reviewer #1: Yes

Reviewer #2: Yes

**********

4. Is the manuscript presented in an intelligible fashion and written in standard English?

Reviewer #1: Yes

Reviewer #2: No

**********

Reviewer #1: The manuscript proposes a coherent graph-based, closed-loop active-learning framework (GTAL) for inverse design of stochastic self-healing composites and reports strong surrogate accuracy and large speedups; in its current form it appears potentially publishable after moderate revision, mainly to tighten methodological clarity, ensure fair/complete benchmarking, and strengthen reproducibility and physical-validity reporting after answering to the following questions.

1. The abstract reports “500-fold acceleration” and “R² > 0.94” and identifies a specific optimum (ρc≈2.5 at 45°) within 30 iterations. Can you clarify exactly how speedup was measured (hardware, runtime definition, I/O excluded or not), how R² was computed (per-task/per-component, test split), and whether the “30 iterations” required new oracle (LBM/DEM) evaluations or was performed on a pre-generated labeled pool?

2. You motivate graphs partly by rotational invariance limitations of CNNs and mention equivariant models. Why were equivariant voxel/point baselines (or equivariant GNN variants) not included, and what is the main novelty beyond combining known components (graph representation + GNN surrogate + BO) in this application setting? Please delineate what is fundamentally new (representation, architecture, loss, or loop design) relative to prior graph-flow surrogates and MeshGraphNets-style fracture learning.

3. The pipeline relies on watershed segmentation and conversion to a heterogeneous multigraph with hydraulic (Maximal Ball) and mechanical (weighted Delaunay) edges. How sensitive are downstream predictions to segmentation artifacts, marker selection, and graph-construction hyperparameters (e.g., connectivity thresholds, pore-throat detection parameters)? Please provide robustness/sensitivity results or at least a protocol for parameter selection.

4. (Eq. 2–3): For hydraulic edges, Eq. (2) uses a Hagen–Poiseuille-style conductance with a shape factor κij. How is κij computed from the CT-derived geometry, and under what flow assumptions is this mapping valid (e.g., laminar, throat length ≈ dij)? For mechanical edges, Eq. (3) defines an ITZ weakness metric ωij via line integration of ∥∇I(x)∥². How is this integral approximated discretely, how is the path γij chosen, and is ωij calibrated/validated against any independent ITZ or fracture measurements (even in-silico)?

5. The LSE-MGN adds spectral edges using the k lowest-frequency Laplacian eigenvectors. What values of k were used, how many long-range edges per node were added, and how sensitive are results to these choices? What is the computational overhead of eigen-decomposition for graphs at your problem scale, and does this overhead affect the claimed overall efficiency in the closed-loop setting?

6. (Eq. 6–7): Eq. (6) includes Lenergy, but its explicit mathematical form is not provided. Please give the full expression for Lenergy, define all symbols (σ, u, etc.), and explain how these quantities are produced by the surrogate (direct outputs vs derived). For Lcont (Eq. 7), how are fluxes qij computed from predicted states, and how are boundary conditions enforced?

7. Numerical Results :

o Table 1: Please define NRMSE normalization precisely and report variability (mean±std over multiple seeds or splits). Were baseline hyperparameters comparably tuned, and were all models trained on the same information budget (data size, augmentation, early stopping)?

o Table 2: VRAM/time per sample depends strongly on hardware, implementation, and batch size. Please specify GPU model, framework versions, and measurement protocol. Also clarify whether preprocessing (graph build, Laplacian eigenvectors) is included in the per-sample time for graph methods.

8. Conclusion You acknowledge reliance on synthetic data and a potential sim-to-real gap for noisy experimental CT and segmentation artifacts, and propose domain adaptation as future work. Can you provide (i) any preliminary evidence on real CT volumes or noise/segmentation perturbation tests, and (ii) a concrete reproducibility plan (release of code, synthetic dataset generator, simulation parameter files, and trained weights) consistent with your data-availability statement?

Reviewer #2: This manuscript presents a novel graph-theoretic active learning (GTAL) framework for the inverse design of stochastic heterogeneous composites, particularly self-healing cementitious materials. The work addresses a significant challenge in materials science by shifting from Euclidean to graph-based representations, enabling efficient and accurate modeling of complex multiphysics behaviors. The integration of geometric deep learning with Bayesian optimization is promising and well-motivated. The manuscript is technically rigorous, methodologically sound, and clearly presented. The results are compelling, demonstrating substantial acceleration and robust performance. However, several aspects require clarification and further validation to strengthen the manuscript for publication.

Several important issues should be addressed to improve the quality and clarity of the manuscript:

Major Comments:

• The authors position their work as a departure from Euclidean deep learning and emphasize the novelty of their graph-based approach. However, the literature review, while comprehensive, could more explicitly delineate the specific advancements over existing graph-based methods (e.g., Pore-GNN, MeshGraphNets). What exactly does the proposed “Long-Short-Edge MeshGraphNet” (LSE-MGN) offer that hybrid or augmented graph networks in prior studies do not? A clearer comparative table or discussion in the introduction would help contextualize the incremental contribution.

• The methodology for converting volumetric tomography into a “permutation-invariant heterogeneous multigraph” is a core contribution. However, the description of the semantic segmentation and graph discretization pipeline (Fig. 1) lacks sufficient technical detail for reproducibility. Please provide more specifics on the marker-controlled watershed transform parameters, handling of segmentation artifacts, and how nodes/edges are pruned or validated. Additionally, how is the “interface weakness metric” ωij normalized or calibrated against physical ITZ strength measurements?

• The introduction of spectral edges via graph Laplacian eigenvectors is innovative. However, the choice of the number of eigenvectors k and the threshold for creating “long-range” edges is not justified. An ablation study varying k would strengthen the argument that this mechanism is essential and optimally configured. Furthermore, how does the spectral edge formulation ensure physical interpretability, or could it introduce non-physical connections that distort mechanical predictions?

• The inclusion of Lcont and Lenergy is commendable and aligns with current trends in physics-informed machine learning. However, the weighting coefficients λcont and λenergy are not discussed. Were these determined via hyperparameter optimization? A sensitivity analysis on these weights would help readers understand their influence on model performance and physical consistency.

• The use of 5,000 synthetically generated microstructures is appropriate for method validation but raises concerns about generalizability to real materials. The authors acknowledge this under “Limitations,” but the manuscript would benefit from a preliminary test on at least one experimental CT scan (even if from a different study) to demonstrate the pipeline’s robustness to noise, partial volume effects, and segmentation errors.

• The Bayesian optimization component is well-described, but some details are missing: How is the GP kernel’s length scale initialized? Is the acquisition function optimized globally or with local methods? How many high-fidelity simulations (LBM/DEM) were actually run during the 30-iteration loop, and what was the total computational cost compared to a full factorial search? A breakdown of the active learning overhead would be insightful.

• The reported R2>0.94 and 500× acceleration are impressive. However, the comparison to “standard GAT” and “PointNet+” seems somewhat mismatched, these architectures are not inherently designed for coupled multiphysics. Including a more relevant baseline, such as a graph network trained without spectral edges or without physics-informed losses, would better isolate the contribution of each novel component.

• The attention visualization in Fig. 5 is a nice addition, but the analysis is qualitative. Quantifying the correlation between attention weights and known physical metrics (e.g., stress concentration factors, ITZ thickness) would strengthen the claim that the model “autonomously learned” physically meaningful features. Additionally, how stable are these attention patterns across different microstructures?

• The manuscript highlights the efficiency of the graph representation over voxel grids. However, how does the node/edge count scale with domain size or resolution? What is the computational overhead of graph construction compared to CNN inference? A brief complexity analysis (time/memory vs. number of entities) would help readers assess scalability for larger or higher-resolution volumes.

• The authors state that “all relevant data are within the manuscript and its Supporting Information files,” but given the complexity of the pipeline, sharing code (even in a anonymized repository) would greatly enhance reproducibility. If code cannot be shared, a more detailed pseudo-algorithm in the supplement for key steps (graph construction, LSE-MGN architecture, active learning loop) is recommended.

•**********

what does this mean?). If published, this will include your full peer review and any attached files.

If you choose “no”, your identity will remain anonymous but your review may still be made public.

**Do you want your identity to be public for this peer review?**  For information about this choice, including consent withdrawal, please see our Privacy Policy

Reviewer #1: No

Reviewer #2: No

**********

[NOTE: If reviewer comments were submitted as an attachment file, they will be attached to this email and accessible via the submission site. Please log into your account, locate the manuscript record, and check for the action link "View Attachments". If this link does not appear, there are no attachment files.]

To ensure your figures meet our technical requirements, please review our figure guidelines: https://journals.plos.org/plosone/s/figures

You may also use PLOS’s free figure tool, NAAS, to help you prepare publication quality figures: https://journals.plos.org/plosone/s/figures#loc-tools-for-figure-preparation.

NAAS will assess whether your figures meet our technical requirements by comparing each figure against our figure specifications.

# Author comment: Author response to Decision Letter 1

**DOI:** 10.1371/journal.pone.0353692.r002

20 Mar 2026

Please see the attached.

Attachment Submitted filename: response-letter.pdf

# Peer review: Decision Letter 1

**DOI:** 10.1371/journal.pone.0353692.r003

**Authors:** Mohammad Maghami

2 Jun 2026

Dear Dr. Yang,

Thank you for submitting your manuscript to PLOS ONE. After careful consideration, we feel that it has merit but does not fully meet PLOS ONE’s publication criteria as it currently stands. Therefore, we invite you to submit a revised version of the manuscript that addresses the points raised during the review process.

Please submit your revised manuscript by Jul 16 2026 11:59PM. If you will need more time than this to complete your revisions, please reply to this message or contact the journal office at plosone@plos.org. When you're ready to submit your revision, log on to https://www.editorialmanager.com/pone/ and select the 'Submissions Needing Revision' folder to locate your manuscript file.

- A letter that responds to each point raised by the academic editor and reviewer(s). You should upload this letter as a separate file labeled 'Response to Reviewers'.
- A marked-up copy of your manuscript that highlights changes made to the original version. You should upload this as a separate file labeled 'Revised Manuscript with Track Changes'.
- An unmarked version of your revised paper without tracked changes. You should upload this as a separate file labeled 'Manuscript'.

If you would like to make changes to your financial disclosure, please include your updated statement in your cover letter. Guidelines for resubmitting your figure files are available below the reviewer comments at the end of this letter.

If applicable, we recommend that you deposit your laboratory protocols in protocols.io to enhance the reproducibility of your results. Protocols.io assigns your protocol its own identifier (DOI) so that it can be cited independently in the future. For instructions see: https://journals.plos.org/plosone/s/submission-guidelines#loc-laboratory-protocols. Additionally, PLOS ONE offers an option for publishing peer-reviewed Lab Protocol articles, which describe protocols hosted on protocols.io. Read more information on sharing protocols at https://plos.org/protocols?utm_medium=editorial-email&utm_source=authorletters&utm_campaign=protocols.

As the corresponding author, your ORCID iD is verified in the submission system and will appear in the published article. PLOS supports the use of ORCID, and we encourage all coauthors to register for an ORCID iD and use it as well. Please encourage your coauthors to verify their ORCID iD within the submission system before final acceptance, as unverified ORCID iDs will not appear in the published article. *Only*  the individual author can complete the verification step; PLOS staff *cannot*  verify ORCID iDs on behalf of authors.

We look forward to receiving your revised manuscript.

Kind regards,

Mohammad Reza Maghami

Academic Editor

PLOS One

Journal Requirements:

If the reviewer comments include a recommendation to cite specific previously published works, please review and evaluate these publications to determine whether they are relevant and should be cited. There is no requirement to cite these works unless the editor has indicated otherwise.

[Note: HTML markup is below. Please do not edit.]

Reviewers' comments:

Reviewer's Responses to Questions

**Comments to the Author**

Reviewer #2: (No Response)

Reviewer #3: (No Response)

Reviewer #4: (No Response)

Reviewer #5: All comments have been addressed

**********

2. Is the manuscript technically sound, and do the data support the conclusions?

Reviewer #2: (No Response)

Reviewer #3: (No Response)

Reviewer #4: Yes

Reviewer #5: Yes

**********

3. Has the statistical analysis been performed appropriately and rigorously?

Reviewer #2: (No Response)

Reviewer #3: (No Response)

Reviewer #4: N/A

Reviewer #5: Yes

**********

4. Have the authors made all data underlying the findings in their manuscript fully available?

The PLOS Data policy

Reviewer #2: (No Response)

Reviewer #3: (No Response)

Reviewer #4: Yes

Reviewer #5: Yes

**********

5. Is the manuscript presented in an intelligible fashion and written in standard English?

Reviewer #2: (No Response)

Reviewer #3: (No Response)

Reviewer #4: Yes

Reviewer #5: Yes

**********

Reviewer #2: The comments have not been addressed accurately and comprehensively.

The manuscript titled "Design and Performance Analysis of a Simplified Hybrid Modulation Method for Dual Active Bridge Powering Inverters" proposes a simplified hybrid modulation strategy that integrates Single Phase Shift (SPS) and Triple Phase Shift (TPS) techniques for operating Dual Active Bridge (DAB) converters. The hybrid method aims to optimize efficiency at low power using TPS while maintaining a simpler control structure using SPS at higher power levels. A single PI controller and a hysteresis-based controller are used to manage power regulation and modulation transitions, respectively. The proposed method is validated through small-signal modeling, simulations, and experimental results using a 500 W DAB-inverter prototype. The study highlights a trade-off between efficiency and dynamic response, favoring efficiency.

Several important issues should be addressed to improve the quality and clarity of the manuscript:

Major Comments:

• The hybrid approach combining SPS and a specific TPS operating mode is well-conceived and addresses the challenge of improving low-power efficiency without significantly increasing control complexity. This contribution is both relevant and practical for real-time applications.

• The paper is well-organized, and each section logically flows into the next. The control strategy is explained clearly with supporting figures (block diagrams and flowcharts), making the concept accessible to readers familiar with power electronics.

• The small-signal modeling for both SPS and TPS modes is a strong point of the paper. However, the derivation steps could be more detailed in the main body rather than being briefly stated with equations. Additional discussion on the limitations of linearization assumptions would be valuable.

• The simulation results match the experimental data well and effectively demonstrate both steady-state and transient performance under different load conditions. Still, quantifying switching losses and core losses in TPS vs SPS operation would enhance the comparative analysis.

• The use of a single PI controller for both modes simplifies implementation. However, the authors acknowledge and demonstrate that this comes at the cost of a slower dynamic response. Exploring adaptive control or gain scheduling could be mentioned as future work.

• Overall, figures are informative and well-annotated. It would be beneficial to highlight key transition points in the transient waveforms and clearly mark switching instances in the control diagrams.

• The decision to switch between TPS and SPS at 30% rated power is justified, but it lacks sensitivity analysis. It would be insightful to evaluate how the system responds to varying this threshold (e.g., 25%, 40%) and the impact on efficiency and stability.

• While the paper cites many relevant works, the reference list is relatively limited and somewhat weak in recent advancements and broader perspectives. The authors are encouraged to include and discuss the following relevant and more recent works to enrich the discussion and demonstrate awareness of the latest research trends:

� https://doi.org/10.1016/j.jestch.2019.08.006

� https://doi.org/10.1016/j.susoc.2024.06.004

� https://doi.org/10.3390/wind5010004

• The proposed control strategy is tested on a 500 W prototype. Comments on scalability to higher power levels (e.g., kilowatt range) and the potential challenges (thermal, control latency, etc.) would strengthen the paper.

• Some equations (especially Eq. 6 and Eq. 7) would benefit from clearer notations and variable descriptions. Also, ensure consistency in symbols (e.g., ϕ vs. ).

• Language is mostly clear, but a few grammar improvements and edits would enhance readability (e.g., “this pose a significant challenge” → “this poses a significant challenge”).

This is a solid and well-structured article with clear practical relevance in the field of power electronics and DAB converter control. With minor revisions, especially in reference strengthening and clarity of technical presentation, it is suitable for publication. The proposed method is valuable for applications involving renewable energy and inverter-based systems.

Reviewer #3: The manuscrip presents a compelling integration of geometric deep learning, multiphysics surrogate modeling, and Bayesian optimization to address the inverse design of self-healing cementitious materials, and while the work demonstrates considerable innovation and strong computational performance, I recommend a major revision prior to publication due to several substantive methodological and validation gaps that must be resolved to fully substantiate the authors’ claims. The proposed framework’s core strength lies in its departure from traditional Euclidean voxel-based architectures in favor of a permutation-invariant heterogeneous multigraph that simultaneously encodes hydraulic conductivity, mechanical force chains, and spectral long-range connectivity, thereby enabling the Long-Short-Edge MeshGraphNet to capture both local interfacial transition zone dynamics and global stress redistribution with remarkable efficiency. The reported acceleration of approximately one hundred thousand fold relative to coupled Lattice Boltzmann and Discrete Element Method solvers, coupled with R-squared values exceeding zero point nine four across both permeability and microcapsule survival tasks, represents a significant advancement in computational materials science, and the successful autonomous discovery of a non-intuitive microcapsule geometry featuring an aspect ratio of two point five oriented at forty-five degrees relative to the shear plane convincingly illustrates the practical utility of the closed-loop active learning paradigm. However, several critical limitations undermine the robustness and generalizability of the findings as currently presented. First, the reliance on entirely synthetic training data generated via random sequential packing and fractal pore algorithms introduces a substantial sim-to-real gap that is only superficially addressed through a single synchrotron microtomography validation yielding an R-squared of zero point eight eight, which is insufficient to claim broad experimental applicability; the manuscript must include either a multi-specimen experimental campaign spanning diverse cement chemistries and water-to-cement ratios or a rigorous domain adaptation protocol that quantifies how segmentation artifacts, noise, and real-world interfacial transition zone heterogeneity propagate through the graph construction pipeline and affect surrogate predictions, particularly given that the synthetic dataset completely neglects realistic capsule size distributions, curing-induced microstructural evolution, and the complex porosity gradients inherent to actual cement hydration. Second, the physics-informed loss functions, while conceptually sound in enforcing mass continuity and strain-energy compatibility, lack a deeper theoretical justification for their weighting scheme, as the current sensitivity analysis reveals that removing the energy constraint degrades survival rate prediction by zero point zero six zero, yet the manuscript does not explore how these soft constraints interact with the stochastic noise inherent in DEM labeling or whether hard constraint enforcement via projection layers might yield more thermodynamically consistent predictions. Third, the Bayesian optimization loop treats the graph neural network as a deterministic surrogate and models residuals using a Gaussian process, which is computationally convenient but fails to leverage the intrinsic epistemic uncertainty of the GNN itself; integrating ensemble-based or Monte Carlo dropout uncertainty directly into the acquisition function would provide a more principled exploration-exploitation balance and should be benchmarked against the current Expected Improvement strategy, especially considering that the anisotropic Matérn-5/2 kernel length scales may become poorly conditioned in higher-dimensional synthesis spaces, potentially leading to premature convergence or overconfident acquisition decisions. Fourth, the spectral long-range edge augmentation, though effective at mitigating oversmoothing, introduces a preprocessing overhead of approximately one hundred eighty milliseconds per graph due to eigendecomposition, and the manuscript does not adequately discuss how this scales to industrial-resolution domains exceeding five hundred cubed voxels or how dynamic topological changes during crack propagation would be handled in a time-dependent setting, which is crucial for modeling the actual healing process that involves time-dependent diffusive-reactive kinetics rather than static equilibrium states. Fifth, the physical interpretation of the discovered optimal configuration requires more rigorous mechanistic validation beyond geometric intuition; the claim that a forty-five degree orientation minimizes projected cross-sectional area during mixing while simultaneously maximizing crack interception probability must be explicitly quantified through probabilistic fracture mechanics analysis or discrete crack tracking simulations to confirm that the identified optimum genuinely resolves the conflicting constraints rather than exploiting a statistical artifact of the synthetic dataset, and the authors should additionally demonstrate how the predicted permeability evolves post-fracture and healing to validate the full functional cycle. Additionally, the reproducibility framework requires substantial enhancement, as the manuscript references supplementary algorithms and sensitivity analyses but omits critical details regarding hyperparameter selection for the multi-head attention mechanism, the exact formulation of the interface weakness metric omega ij, and the boundary condition implementations for the physics-informed losses, all of which are essential for independent replication. Finally, the computational efficiency analysis should be expanded to include energy consumption metrics or floating-point operation counts, and the comparison baselines should include at least one state-of-the-art equivariant graph network trained on identical heterogeneous inputs to ensure the performance gains are not merely attributable to architectural tuning differences. Addressing these concerns through comprehensive experimental validation, theoretical refinement of the loss weighting and uncertainty quantification mechanisms, mechanistic verification of the discovered microstructural optimum, and complete documentation of the preprocessing and training protocols will significantly elevate the manuscript’s impact and ensure its findings are robust, reproducible, and broadly applicable to the materials discovery community, and I therefore strongly encourage the authors to undertake these major revisions so that this otherwise highly innovative framework can reach its full publication potential.

Reviewer #4: 1. The abstract contains an inconsistency in acceleration claims. The submission metadata mentions 500-fold acceleration, while the manuscript abstract reports approximately 1.1 × 10⁵-fold acceleration. These claims must be made consistent.

2. The term “experimental validation” is overstated. Most validation appears to be based on synthetic microstructures and high-fidelity numerical simulations. Only one real synchrotron µCT specimen is mentioned as preliminary validation, so the word “experimental” should be used cautiously.

3. The study is mainly simulation-driven, not experimentally verified. No physical fabrication, mechanical testing, permeability testing, or self-healing performance test of the optimized capsule configuration is presented.

4. The optimized design claim needs physical confirmation. The conclusion that anisotropic microcapsules with aspect ratio around 2.5 and 45° orientation are optimal should be validated using additional DEM/LBM cases and, ideally, experimental capsule survival/crack-interception tests.

5. The healing performance objective is indirect. The framework optimizes permeability and microcapsule survival rate, but actual healing efficiency, crack closure, healing-agent release, transport, polymerization, or strength recovery is not directly modeled.

6. The self-healing mechanism needs stronger material realism. Microcapsule shell material, healing-agent viscosity, capsule rupture threshold, shell thickness, capsule–matrix bonding, and post-crack release behavior should be defined more clearly.

7. The synthetic dataset generation requires stronger justification. The 5,000 microstructures are generated using random sequential packing and Diamond-Square pore synthesis, but it is unclear how closely these synthetic structures represent real cement paste or concrete microstructures.

8. The sim-to-real gap remains a major limitation. Validation on one real µCT scan is not sufficient to claim robust generalization to experimental cementitious composites. More real datasets with different w/c ratios, curing ages, resolutions, and segmentation noise are needed.

9. The real µCT validation is statistically weak. The manuscript reports R² for only three orthogonal permeability values from one specimen. Such a small validation set cannot support strong claims about real-data generalization.

Reviewer #5: Accept in the present form, the authors have satisfactorily addressed all the comments and therefore recommended for acceptance.

**********

what does this mean?). If published, this will include your full peer review and any attached files.

If you choose “no”, your identity will remain anonymous but your review may still be made public.

**Do you want your identity to be public for this peer review?**  For information about this choice, including consent withdrawal, please see our Privacy Policy

Reviewer #2: No

Reviewer #3: No

Reviewer #4: No

Reviewer #5: No

**********

[NOTE: If reviewer comments were submitted as an attachment file, they will be attached to this email and accessible via the submission site. Please log into your account, locate the manuscript record, and check for the action link "View Attachments". If this link does not appear, there are no attachment files.]

To ensure your figures meet our technical requirements, please review our figure guidelines: https://journals.plos.org/plosone/s/figures

You may also use PLOS’s free figure tool, NAAS, to help you prepare publication quality figures: https://journals.plos.org/plosone/s/figures#loc-tools-for-figure-preparation.

NAAS will assess whether your figures meet our technical requirements by comparing each figure against our figure specifications.

# Author comment: Author response to Decision Letter 2

**DOI:** 10.1371/journal.pone.0353692.r004

15 Jun 2026

Kindly find uploaded response letter.

Attachment Submitted filename: Response_to_Reviewers_R2.pdf

# Peer review: Decision Letter 2

**DOI:** 10.1371/journal.pone.0353692.r005

**Authors:** Mohammad Maghami

28 Jun 2026

Graph-Theoretic Active Learning for the Closed-Loop Discovery of Stochastic Heterogeneous Composites

PONE-D-25-64550R2

Dear Dr. Yang,

We’re pleased to inform you that your manuscript has been judged scientifically suitable for publication and will be formally accepted for publication once it meets all outstanding technical requirements.

Within one week, you’ll receive an e-mail detailing the required amendments. When these have been addressed, you’ll receive a formal acceptance letter and your manuscript will be scheduled for publication.

An invoice will be generated when your article is formally accepted. Please note, if your institution has a publishing partnership with PLOS and your article meets the relevant criteria, all or part of your publication costs will be covered. Please make sure your user information is up-to-date by logging into Editorial Manager at Editorial Manager® and clicking the ‘Update My Information' link at the top of the page. For questions related to billing, please contact billing support.

If your institution or institutions have a press office, please notify them about your upcoming paper to help maximize its impact. If they’ll be preparing press materials, please inform our press team as soon as possible -- no later than 48 hours after receiving the formal acceptance. Your manuscript will remain under strict press embargo until 2 pm Eastern Time on the date of publication. For more information, please contact onepress@plos.org.

Kind regards,

Mohammad Reza Maghami

Academic Editor

PLOS One

Additional Editor Comments (optional):

Reviewers' comments:

Reviewer's Responses to Questions

**Comments to the Author**

Reviewer #2: (No Response)

Reviewer #3: (No Response)

Reviewer #4: All comments have been addressed

**********

2. Is the manuscript technically sound, and do the data support the conclusions?

Reviewer #2: Yes

Reviewer #3: (No Response)

Reviewer #4: Yes

**********

3. Has the statistical analysis been performed appropriately and rigorously?

Reviewer #2: Yes

Reviewer #3: (No Response)

Reviewer #4: N/A

**********

4. Have the authors made all data underlying the findings in their manuscript fully available?

The PLOS Data policy

Reviewer #2: Yes

Reviewer #3: (No Response)

Reviewer #4: Yes

**********

5. Is the manuscript presented in an intelligible fashion and written in standard English?

Reviewer #2: Yes

Reviewer #3: (No Response)

Reviewer #4: Yes

**********

Reviewer #2: All comments have been thoroughly addressed in the revised manuscript.

We believe these revisions have strengthened the clarity and scientific rigor of our work.

Reviewer #3: no further comments to the author no further comments to the author no further comments to the author

Reviewer #4: The authors have addressed all the comments and the manuscript can be accepted in its current form...

**********

what does this mean?). If published, this will include your full peer review and any attached files.

If you choose “no”, your identity will remain anonymous but your review may still be made public.

**Do you want your identity to be public for this peer review?**  For information about this choice, including consent withdrawal, please see our Privacy Policy

Reviewer #2: No

Reviewer #3: No

Reviewer #4: No

**********

# Editor report: Acceptance letter

**DOI:** 10.1371/journal.pone.0353692.r006

**Authors:** Mohammad Maghami

PONE-D-25-64550R2

PLOS One

Dear Dr. Yang,

I'm pleased to inform you that your manuscript has been deemed suitable for publication in PLOS One. Congratulations! Your manuscript is now being handed over to our production team.

At this stage, our production department will prepare your paper for publication. This includes ensuring the following:

* All references, tables, and figures are properly cited

* All relevant supporting information is included in the manuscript submission,

* There are no issues that prevent the paper from being properly typeset

You will receive further instructions from the production team, including instructions on how to review your proof when it is ready. Please keep in mind that we are working through a large volume of accepted articles, so please give us a few days to review your paper and let you know the next and final steps.

Lastly, if your institution or institutions have a press office, please let them know about your upcoming paper now to help maximize its impact. If they'll be preparing press materials, please inform our press team within the next 48 hours. Your manuscript will remain under strict press embargo until 2 pm Eastern Time on the date of publication. For more information, please contact onepress@plos.org.

You will receive an invoice from PLOS for your publication fee after your manuscript has reached the completed accept phase. If you receive an email requesting payment before acceptance or for any other service, this may be a phishing scheme. Learn how to identify phishing emails and protect your accounts at https://explore.plos.org/phishing.

If we can help with anything else, please email us at customercare@plos.org.

Thank you for submitting your work to PLOS ONE and supporting open access.

Kind regards,

PLOS ONE Editorial Office Staff

on behalf of

Dr. Mohammad Reza Maghami

Academic Editor

PLOS One
